# Quantum Chemistry as a Tool in Asymmetric Biocatalysis: Limonene Epoxide Hydrolase Test Case**

**DOI:** 10.1002/anie.201300594

**Published:** 2013-03-19

**Authors:** Maria E S Lind, Fahmi Himo

**Affiliations:** Department of Organic Chemistry, Arrhenius Laboratory, Stockholm University10691 Stockholm (Sweden) E-mail: himo@organ.su.se

**Keywords:** biocatalysis, enantioselectivity, enzymes, quantum chemistry, transition states

Quantum chemical models of enzyme active sites have in recent years proven to be a very powerful tool in the elucidation of enzymatic reaction mechanisms.^1^ In the so-called cluster approach, a limited part of the enzyme around the active site is cut out and treated using relatively accurate electronic structure methods, typically hybrid density functional theory (DFT). The missing enzyme surrounding is approximated by a homogeneous polarizable continuum model with some assumed dielectric constant.^2^ A large variety of enzymatic systems have been investigated quite successfully using this approach and a wealth of mechanistic insight has been gained.^3^ A few years ago, active site models consisted typically of less than 100 atoms. However, with today’s computers and using the same computational protocol, it is possible to treat more than 250 atoms quite routinely. These developments have paved the way for wider applications of the cluster approach, beyond the pure mechanistic investigations.

To investigate and explain sources of various kinds of selectivities, in particular enantioselectivity, one has typically to reproduce relative transition-state energies on the order of 1 kcal mol^−1^. The accuracy of modern DFT methods has been proven to be sufficiently high to achieve this. In particular, these methods have in recent years been applied very successfully to a multitude of problems in the field of asymmetric homogenous catalysis.^4^ Modeling enzymatic enantioselectivity with the cluster approach has remained somewhat out of reach because larger active-site models are in general required to create the chiral environment provided by the enzyme. Herein, we will demonstrate that this kind of modeling approach is indeed able to reproduce and rationalize enantioselectivity in enzymes and has thus the potential to become a valuable tool also in the field of asymmetric biocatalysis.

Today enzymes are increasingly used in synthetic chemistry for the production of base and fine chemicals.^5^ Indeed, biocatalytic processes are starting to replace transition-metal-catalyzed reactions for large-scale production of drug compounds.^6^ One of the most attractive features of enzymes in this respect is their high levels of selectivity. Various engineering techniques have been developed to manipulate the properties of enzymes to achieve the desired function. These techniques range from pure combinatorial approaches to semi-rational and fully rational designs, which are based on the detailed knowledge of the structure and mechanism of the enzymes. Theoretical methodology in asymmetric biocatalysis has relied mainly on substrate docking or molecular dynamics simulations of the enzyme–substrate (ES) complexes in the ground state.^7^, ^8^ To some extent, this has been successful in qualitatively guiding the experimental work as to which parts of the active site to manipulate and also to provide some rationalization for observed trends. These approaches are, however, inherently deficient since they do not consider the transition states and can thus not be fully quantitative. In recent years, the quantum mechanical/molecular mechanical (QM/MM) approach^9^ has been employed in this field to obtain a more quantitative description.^10^ In particular, the empirical valence bond (EVB) method has been shown to yield very promising results for the case of *Candida antarctica* lipase A.^11^

To examine the capabilities of the cluster approach in terms of reproducing enantioselectivity and mutational effects we have chosen to focus on the enzyme limonene epoxide hydrolase (LEH) from *rhodoccoccus erythropolis* as a test case. The natural substrate for this enzyme is limonene-1,2-epoxide, but the enzyme can catalyze the hydrolysis of a range of other epoxides to their corresponding vicinal diols.^12^ The reaction mechanism of LEH is quite well understood and consists of a single concerted step in which an aspartate (Asp132) abstracts a proton from the nucleophilic water molecule which attacks the epoxide, while another Asp residue (Asp101) protonates the oxirane ring of the substrate. Arg99 positions the carboxylate groups of the two aspartates, while the water molecule is properly positioned in the active site by hydrogen bonds to the Tyr53, Asn55, and Asp132 residues.

The potential for the enzyme to be useful in biocatalysis is limited by the poor enantioselectivity it displays for substrates other than the natural limonene epoxide. For example, cyclopentene oxide (Scheme 1) is hydrolyzed with an enantiomeric excess (*ee*) of only 14 % in favor of the *R*,*R*-configured product.^13^ Recently, Zheng and Reetz employed iterative saturation mutagenesis techniques to engineer LEH mutants which were able to catalyze the desymmetrization of *meso*-cyclopentene oxide to produce either of the *R*,*R*- or *S*,*S*-configured diols with high enantioselectivities (Figure 1).^13^ The ability to generate *R*- or *S*-selective mutants using directed evolution techniques is indeed an impressive success of the experimental procedures. These well-defined experimental results constitute an interesting case to assess the usefulness of the cluster approach. It should be pointed out that as the substrate is a *meso* compound it makes the task of the investigation of the enantioselectivity somewhat easier, since one in fact does not need to consider the differential binding energies of enantiomeric substrates. The latter case is of course a more common scenario in asymmetric biocatalytic applications. Nevertheless, we have in the current work chosen to study LEH-catalyzed desymmetrization of cyclopentene oxide to separate the effects of the mutations on the chemical step from those on the binding step, which will help to better analyze possible sources of errors associated with the cluster approach.

**Figure 1:**
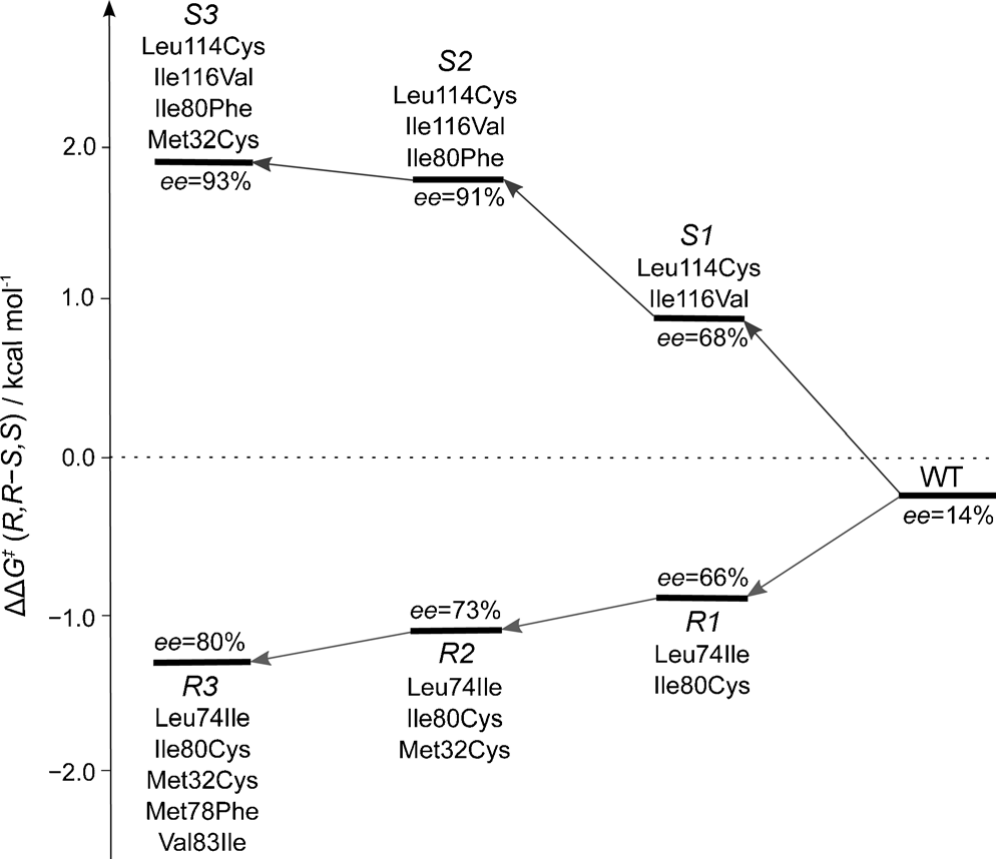
Experimental results of iterative saturation mutagenesis experiments.^13^

**Scheme 1:**
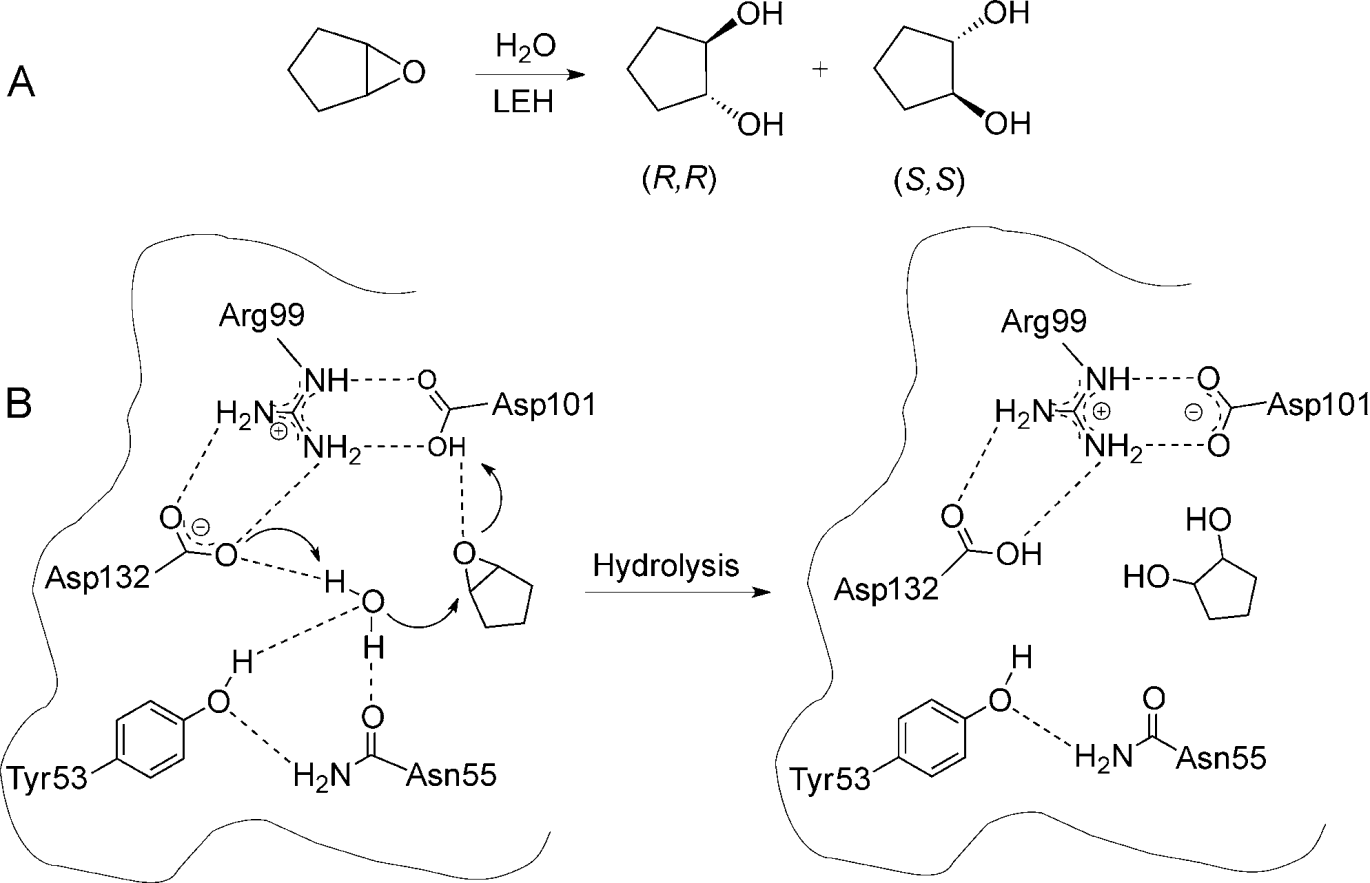
A) LEH-catalyzed desymmetrization reaction considered in the present work. B) Reaction mechanism of LEH.

Previous quantum chemical calculations using a relatively small model of the active site (80 atoms) confirmed the reaction mechanism shown in Scheme 1 and resolved some issues regarding the stereoselectivity of limonene hydrolysis, issues which were shown to be inherent to the limonene substrate itself.^14^ QM/MM calculations have also been performed recently, reaching similar conclusions regarding the reaction mechanism.^15^

Here, a large active-site model of LEH was designed based on the X-ray crystal structure of the wild-type (WT) enzyme crystallized with heptanamide as a ligand (PDB 1NWW).12d It consists of 259 atoms and includes the following groups (Figure 2): the Asp132-Arg99-Asp101 catalytic triad, the nucleophilic water and the two residues hydrogen-bonding to it, Tyr53 and Asn55, as well as other groups that define the active-site cavity, namely Met78, Leu74, Ile80, Leu35, Leu103, Met32, Val83, Leu114, and Ile116. Hydrogen atoms were added manually and the ligand in the active site was replaced by the substrate with the epoxide oxygen atom positioned within hydrogen-bonding distance to Asp101. As shown in Figure 2, the various amino acids were truncated to reduce the size of the model. The truncation points (asterisks in Figure 2) were kept fixed during the geometry optimizations to maintain the overall structure of the active site (see the Supporting Information for a list of locked centers in all models). This coordinate-locking scheme is a very common, and in many cases necessary, procedure in the cluster approach and has over the years been shown to yield very good results, in particular when the model is large enough.^1^ The geometries were optimized at the B3LYP/6-31G(d,p) level of theory (see Computational Details in the Supporting Information). Large models of the size used here suffer very commonly from multiple-minima problems which can be quite severe, and can lead to unreliable energies. In the present work, we have very carefully, by visual inspection and by overlaying the optimized structures of the stationary points, made sure that groups which are not directly participating in the reaction are in the same local minima throughout the reaction.

**Figure 2:**
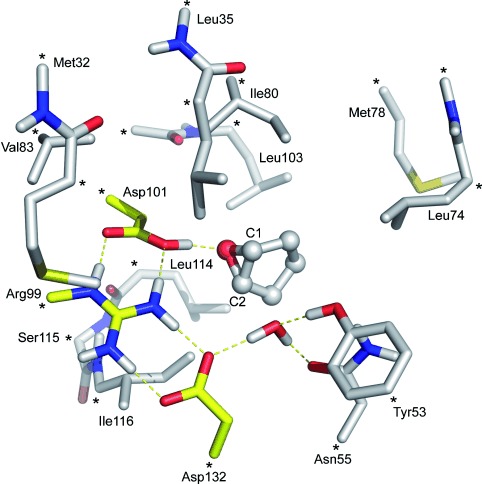
Optimized structure of the active-site model of LEH. The catalytically active residues are shown in yellow. Asterisks indicate positions fixed to their crystallographic coordinates. For clarity, only selected hydrogen atoms are shown.

The optimized structure of the ES complex is displayed in Figure 2. Cyclopentene oxide is somewhat smaller than the natural limonene epoxide substrate and therefore fits in the active-site cavity without major conformational changes of the side chains as compared to the crystal structure. As expected, the substrate is positioned through a hydrogen bond to Asp101 while the water molecule forms hydrogen bonds to Tyr53 and Asn55, as well as to Asp132, which will act as the general base. Next, we optimized the transition states (TSs) for the opening of the oxirane ring at either of the two carbon centers which we will refer to as C1 and C2, respectively, thus leading to either the *S*,*S*- or *R*,*R*-configured products (see Figure 2 for labeling). As demonstrated in the previous quantum chemical study,^14^ the reaction is calculated to take place in one concerted step in which the nucleophilic attack and ring-opening take place at the same time as the activation of the water molecule by Asp132 and the protonation of the epoxide oxygen atom by Asp101 (Figure 3).

**Figure 3:**
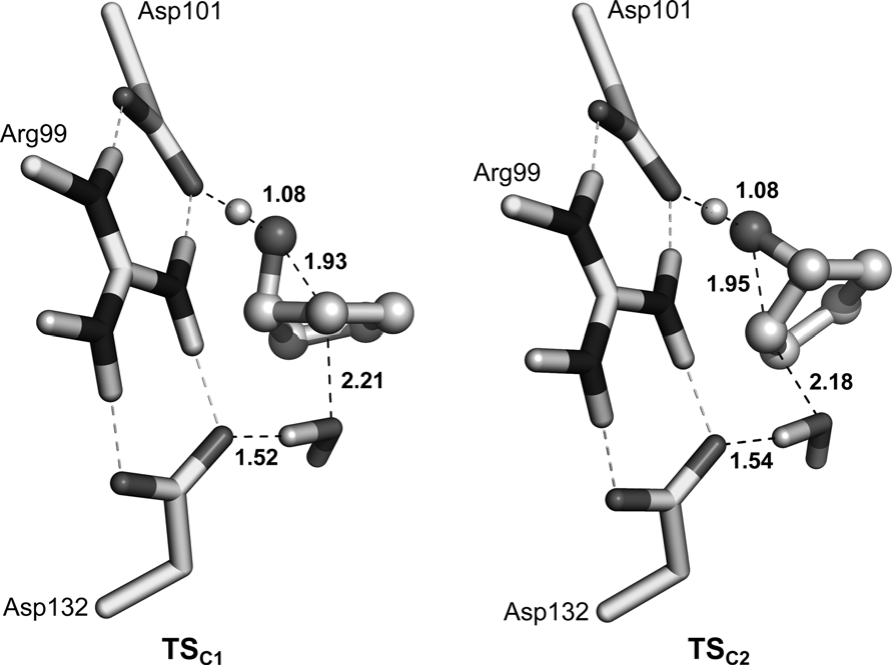
Optimized transition-state structures, for the WT, of TS_C1_ and TS_C2_, which result in the *S*,*S*- or *R*,*R*-configured products, respectively. Selected distances are given in Angstroms. Note that for clarity the figures show only a small part of the active-site model.

The energy barriers were calculated at the B3LYP/6-311+G(2d,2p) level of theory and include corrections for the zero-point, solvation, and dispersion effects (see the Supporting Information). For the WT structure, the computed barriers were found to be almost identical, 15.7 and 15.6 kcal mol^−1^, for obtaining the *S*,*S*- and *R*,*R*-configured products, respectively. This result is in a very good agreement with the experimental observation of a small 14 % *ee* obtained in favor of the *R*,*R*-configured product, and corresponds to an energy difference of 0.2 kcal mol^−1^. As mentioned above, the active-site cavity of the WT is somewhat too large for the cyclopentene oxide substrate and can therefore accommodate attacks at C1 and C2 equally well because the substrate can be displaced in one direction or the other (Figure 3) with the same energetic penalty. This scenario results in very similar barriers and hence very poor selectivity.

The results so far show that the active-site model can reproduce and rationalize the enantioselectivity of the WT enzyme. Next, the cluster model was altered according to the experimental mutations (see Figure 1 for definitions) and all TSs for attacks on C1 and C2 were re-optimized. The calculated barriers are presented in Table 1 and the barrier differences are compared to the experimentally determined enantioselectivities in Figure 4.

**Figure 4:**
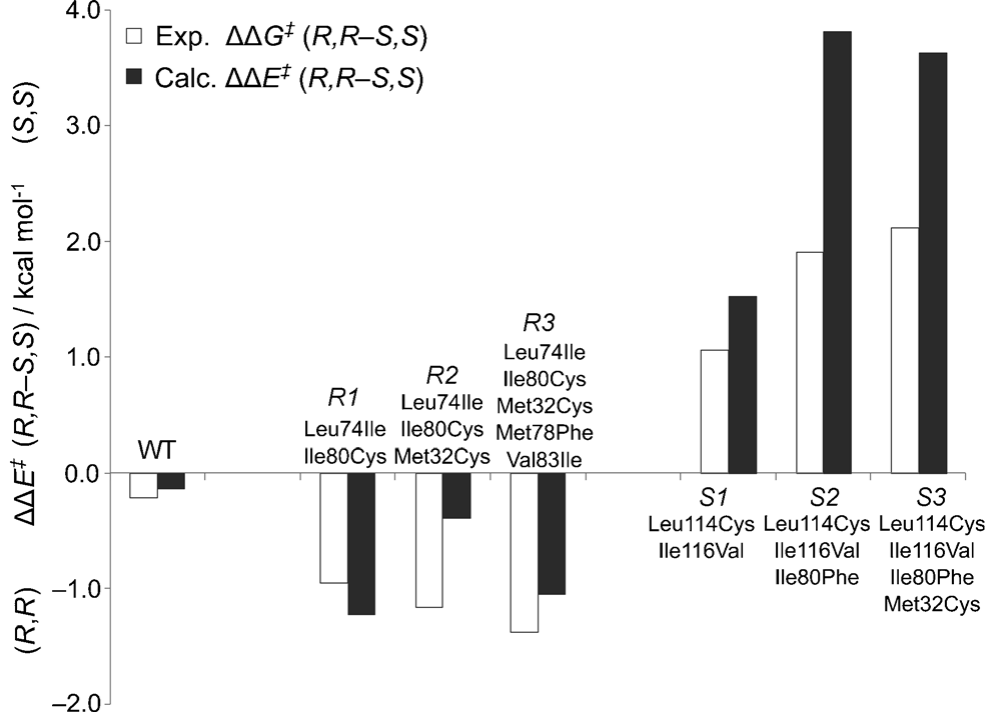
Comparison between experimental and calculated differences in activation barriers for WT and mutants.

**Table 1:** Calculated absolute and relative activation barriers (in kcal mol^−1^) for WT and all mutants

	TS_C1_	TS_C2_	ΔΔ*E*^≠^_calc._	ΔΔ*G*^≠^_expt_[a]
WT	15.7	15.6	−0.1	−0.2
Mutant *R1*	14.3	13.1	−1.2	−0.9
Mutant *R2*	14.0	13.6	−0.4	−1.1
Mutant *R3*	15.3	14.3	−1.0	−1.3
Mutant *S1*	14.7	16.1	+1.4	+1.0
Mutant *S2*	13.9	17.5	+3.6	+1.8
Mutant *S3*	13.2	16.6	+3.4	+2.0

[a] Energies as converted from the experimental enantiomeric excesses.

The comparison shows that the cluster model yields very good agreement with the experimental observations. That is, the mutants that experimentally result in improved *R*,*R* selectivity are indeed calculated to have lower barriers for opening at C2, while the mutants that show *S*,*S* selectivity have lower barriers for opening at C1. Taken together, these results must be regarded as outstanding indeed, especially considering that some of the variants contain up to five point mutations.

The energetic preference for the *S*,*S*- or *R*,*R-*configured products can be rationalized by scrutinizing the optimized transition-state structures of the mutants. It turns out that, to a large extent, the mutational effects can be explained by how much steric hindrance they introduce or relieve to prevent or allow the substrate from moving to accommodate attacks on either of the two carbon centers. For example, both the Leu74Ile and Ile80Cys mutations in the double-mutant *R1* variant introduce smaller side chains and make one side of the active site slightly less crowded, and in turn makes the attack on C2 somewhat less hindered because the substrate now can be displaced more easily in that direction. Overall, this double mutation leads to lower barriers for both attacks compared to the WT, but the C2 attack is more favorable (13.1 and 14.3 kcal mol^−1^ for attacks at C2 and C1, respectively), thus yielding a higher selectivity for the *R*,*R*-configured product.

Conversely, in the *S1* mutant both the Leu114Cys and Ile116Val mutations are located on the other side of the substrate and result in decreased bulk there. Therefore, these mutations will now lower the barrier for attack on C1 compared to C2 (14.7 and 16.1 kcal mol^−1^ for attacks at C1 and C2, respectively), thus leading to the *S*,*S*-configured product. The additional mutation introduced in *S2*, Ile80Phe, is located on the other side compared to the two mutations of *S1* and introduces additional bulk there. This mutation results in a higher barrier for attack at C2 and thus the more favorable C1 attack (13.9 and 17.5 kcal mol^−1^ for attacks at C1 and C2, respectively) leads to an increased *S*,*S* selectivity. The calculated effect of the Ile80Phe mutation is however somewhat overestimated compared to experiments (Figure 4). One reason for this could be that the locking scheme makes that residue too rigid in the cluster model.

Interestingly, the Met32Cys mutation appears experimentally in both branches of mutations, that is, it helps in improving both the *R*,*R* and *S*,*S* selectivities (*R1*→*R2* and *S2*→*S3*). The improvement is quite small (66 to 73 % *ee* for *R1*→*R2* and 91 to 93 % *ee* for *S2*→*S3*). Its role is unclear and it is evident that the calculations cannot reproduce the trends correctly (Figure 4). One reason for this discrepancy could be that the Met32Cys introduces a hydrogen-bonding thiol group at the periphery of the cluster model. In the absence of other residues outside, this group turns inward and forms somewhat artificial interactions which result in this disagreement.

Finally, it is interesting to monitor some geometric parameters which can be used as indicators of the amount of steric hindrance put on the substrate during the attack. For example, in the case of the WT enzyme the nucleophilic ∡O-C-O angles are 150.1° and 150.3° for attacks on C1 and C2, respectively, thus showing that the two TSs are very similar (see Figure 3 and the Supporting Information for other geometric parameters). In the *R1* variant, these angles are 148.9° and 150.5°, respectively, showing that the substrate in the C2 attack which leads to the *R*,*R*-configured product is slightly less constrained. In the *S1* variant, the opposite trend is observed; the angles are namely 150.2° and 147.5°, respectively.

To conclude, the calculations presented herein provide convincing results showing that the quantum chemical cluster methodology for modeling enzyme active sites can reproduce and rationalize enantioselectivity quite well. However, although the absolute *R*,*R* or *S*,*S* enantioselectivities are well reproduced by the model, the trends within each branch are not accurately captured. One source of error could be that the mutations introduce larger conformational changes of the active site, changes which are not properly represented in our calculations because we use the WT crystal structure as a starting point for the mutant calculations. Other sources of error could be the usual limitations associated with the cluster approach, such as the use of homogenous solvation instead of the specific field provided by the enzyme surrounding, the coordinate-locking scheme, and the use of enthalpy rather than free energies.

It should be emphasized that the current calculations have followed the standard cluster approach described in many previous reports, with the aim of investigating how well it performs in this kind of situation. That is, the aim has not been to reproduce the particular experimental results at hand, as might be achieved with enough alterations of active-site models or the underlying quantum chemical methods. In that respect, the results are indeed very promising and show that the cluster approach can be a very economic alternative to the more elaborate schemes currently available in the computational chemistry toolbox and can thus be valuable in the field of biocatalysis. More test cases have of course to be investigated to properly evaluate the strengths and limitations. Future studies on the LEH enzyme include assessment of the role of the starting structure on the selectivity. For example, molecular dynamics simulations can be performed first to equilibrate the mutant structures before the cluster model is cut out. These studies are currently underway in our laboratory as well as investigations of more complicated enzymes with enantiomeric substrates which bind differently to the active site.

## Supplementary material

As a service to our authors and readers, this journal provides supporting information supplied by the authors. Such materials are peer reviewed and may be re-organized for online delivery, but are not copy-edited or typeset. Technical support issues arising from supporting information (other than missing files) should be addressed to the authors.
