# Supplementary material for: Quantum Chemistry as a Tool in Asymmetric Biocatalysis: Limonene Epoxide Hydrolase Test Case
Source: Angew Chem Int Ed Engl. 2013 Mar 19;52(17):4563–7. doi: 10.1002/anie.201300594 (PMC3734700; doi:10.1002/anie.201300594)
Supplement: Supplementary file 1 [file anie0052-4563-SD1.pdf]

Angewandte Chemie
Eine Zeitschrift der Gesellschaft Deutscher Chemiker

Supporting Information

© Wiley-VCH 2013

69451 Weinheim, Germany

Quantum Chemistry as a Tool in Asymmetric Biocatalysis: Limonene Epoxide Hydrolase Test Case**

*Maria E. S. Lind and Fahmi Himo**

anie_201300594_sm_miscellaneous_information.pdf

1. Computational Details

All calculations were performed using the hybrid density functional B3LYP[1] as implemented in the Gaussian03 program package.[2] Geometry optimizations were carried out employing the 6-31G(d,p) basis set. More accurate energies were obtained by single-point calculations on the optimized geometries using the larger 6-311+G(2d,2p) basis set. The polarization effects of the enzyme environment not explicitly included in the model were taken into account by single-point calculations using the conductor-like polarizable continuum model (CPCM) [3] with a dielectric constant of 4 and the UA0 radii. Analytical frequencies were calculated at the same level of theory as the geometry optimizations to confirm the nature of the stationary points and also to obtain zero-point energies (ZPE). Fixing coordinates introduces a few small imaginary frequencies, in this case all below 50*i* cm^{-1}. These small frequencies have little impact on the ZPE and can therefore be disregarded. All energies reported in the paper include also the dispersion effect according to the B3LYP-D2 method of Grimme. [4]

[1] a) A. D. Becke, *J. Chem. Phys.* **1993**, *98*, 5648; b) C. Lee, W. Yang, R. G. Parr, *Phys. Rev. B* **1988**, *37*, 785.

[2] Gaussian 03, Revision D.01 and E.01, M. J. Frisch, G. W. Trucks, H. B. Schlegel, G. E. Scuseria, M. A. Robb, J. R. Cheeseman, J. A. Montgomery, Jr., T. Vreven, K. N. Kudin, J. C. Burant, J. M. Millam, S. S. Iyengar, J. Tomasi, V. Barone, B. Mennucci, M. Cossi, G. Scalmani, N. Rega, G. A. Petersson, H. Nakatsuji, M. Hada, M. Ehara, K. Toyota, R. Fukuda, J. Hasegawa, M. Ishida, T. Nakajima, Y. Honda, O. Kitao, H. Nakai, M. Klene, X. Li, J. E. Knox, H. P. Hratchian, J. B. Cross, V. Bakken, C. Adamo, J. Jaramillo, R. Gomperts, R. E. Stratmann, O. Yazyev, A. J. Austin, R. Cammi, C. Pomelli, J. W. Ochterski, P. Y. Ayala, K. Morokuma, G. A. Voth, P. Salvador, J. J. Dannenberg, V. G. Zakrzewski, S. Dapprich, A. D. Daniels, M. C. Strain, O. Farkas, D. K. Malick, A. D. Rabuck, K. Raghavachari, J. B. Foresman, J. V. Ortiz, Q. Cui, A. G. Baboul, S. Clifford, J. Cioslowski, B. B. Stefanov, G. Liu, A. Liashenko, P. Piskorz, I. Komaromi, R. L. Martin, D. J. Fox, T. Keith, M. A. Al-Laham, C. Y. Peng, A. Nanayakkara, M. Challacombe, P. M. W. Gill, B. Johnson, W. Chen, M. W. Wong, C. Gonzalez, and J. A. Pople, Gaussian, Inc., Wallingford CT, 2004.

[3] a) M. Cossi, N. Rega, G. Scalmani, V. Barone, *J. Comput. Chem.* **2003**, *24*, 669; b) V. Barone, M. Cossi, *J. Phys. Chem. A* **1998**, *102*, 1995; c) J. Andzelm, C. Kölmel, A. Klamt, *J. Chem. Phys.* **1995**, *103*, 9312; d) A. Klamt, G. Schüürmann, *J. Chem. Soc., Perkin Trans. 2 (1972–1999)* **1993**, 799.

[4] S. Grimme, *J. Comput. Chem.* **2006**, 27, 1787.

2. Table of Calculated Geometric Parameters

Table S1. Calculated geometric parameters (in Angstrom and degrees) for WT and all mutants.

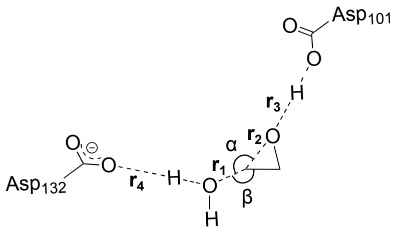

	TS$_{C1}$						TS$_{C2}$					
	r_1	r_2	r_3	r_4	α	β	r_1	r_2	r_3	r_4	α	β
**WT**	2.21	1.93	1.08	1.52	150.1	103.7	2.18	1.95	1.08	1.54	150.3	104.3
**Mutant *R1***	2.23	1.94	1.08	1.52	148.9	102.9	2.19	1.93	1.07	1.53	150.5	103.8
**Mutant *R2***	2.22	1.93	1.08	1.52	149.5	103.3	2.18	1.94	1.07	1.53	150.8	104.3
**Mutant *R3***	2.22	1.93	1.08	1.52	149.2	102.7	2.16	1.95	1.07	1.52	150.6	104.6
**Mutant *S1***	2.21	1.93	1.11	1.56	150.2	103.9	2.20	1.97	1.09	1.56	147.5	102.5
**Mutant *S2***	2.24	1.93	1.11	1.57	150.9	104.6	2.28	1.97	1.09	1.59	142.7	97.9
**Mutant *S3***	2.25	1.94	1.10	1.57	151.1	105.1	2.25	1.99	1.08	1.58	142.9	98.4

3. Absolute Energies and Energy Corrections of Stationary Points

In all calculations a total charge of 0 and multiplicity of 1 were used. All energies are reported in Hartree.

	**Geometry optimization: B3LYP/ 6-31(d,p)**	**Single-point CPCM: B3LYP/ 6-31(d,p)**	**Single-point: B3LYP/ 6-311+(2d,2p)**	**ZPE**	**Dispersion correction**
**WT**					
Reactant	-5350.713212	-5350.764494	-5352.066201	2.305257	-0.383362
TS C1	-5350.680687	-5350.732258	-5352.034041	2.303219	-0.388152
TS C2	-5350.680149	-5350.732280	-5352.032265	2.303185	-0.389536
**Mutant *R*1**					
Reactant	-5630.952658	-5631.006927	-5632.308633	2.220656	-0.364716
TS C1	-5630.922326	-5630.977186	-5632.277735	2.218758	-0.370319
TS C2	-5630.922361	-5630.977959	-5632.278090	2.218781	-0.371113
**Mutant *R2***					
Reactant	-5552.322256	-5552.377093	-5553.661197	2.161323	-0.350955
TS C1	-5552.291744	-5552.347526	-5553.629992	2.159074	-0.356711
TS C2	-5552.291983	-5552.348127	-5553.630015	2.159473	-0.357319
**Mutant *R3***					
Reactant	-5345.873215	-5345.928434	-5347.229891	2.212919	-0.368870
TS C1	-5345.844220	-5345.898403	-5347.200457	2.211500	-0.373477
TS C2	-5345.845585	-5345.900428	-5347.201414	2.212254	-0.374197
**Mutant *S1***					
Reactant	-5591.635316	-5591.688940	-5592.981980	2.191484	-0.358899
TS C1	-5591.604365	-5591.658828	-5592.949852	2.188943	-0.364272
TS C2	-5591.601664	-5591.656538	-5592.947659	2.189574	-0.364392
**Mutant *S2***					
Reactant	-5704.745473	-5704.799298	-5706.119302	2.189209	-0.365000
TS C1	-5704.714435	-5704.769108	-5706.086686	2.186865	-0.372214
TS C2	-5704.708387	-5704.763738	-5706.082363	2.186623	-0.369879
**Mutant *S3***					
Reactant	-5626.113484	-5626.167961	-5627.469365	2.130548	-0.354397
TS C1	-5626.083287	-5626.138274	-5627.438301	2.127491	-0.360930
TS C2	-5626.076742	-5626.132566	-5627.433713	2.127474	-0.359202

4. Cartesian coordinates of stationary points

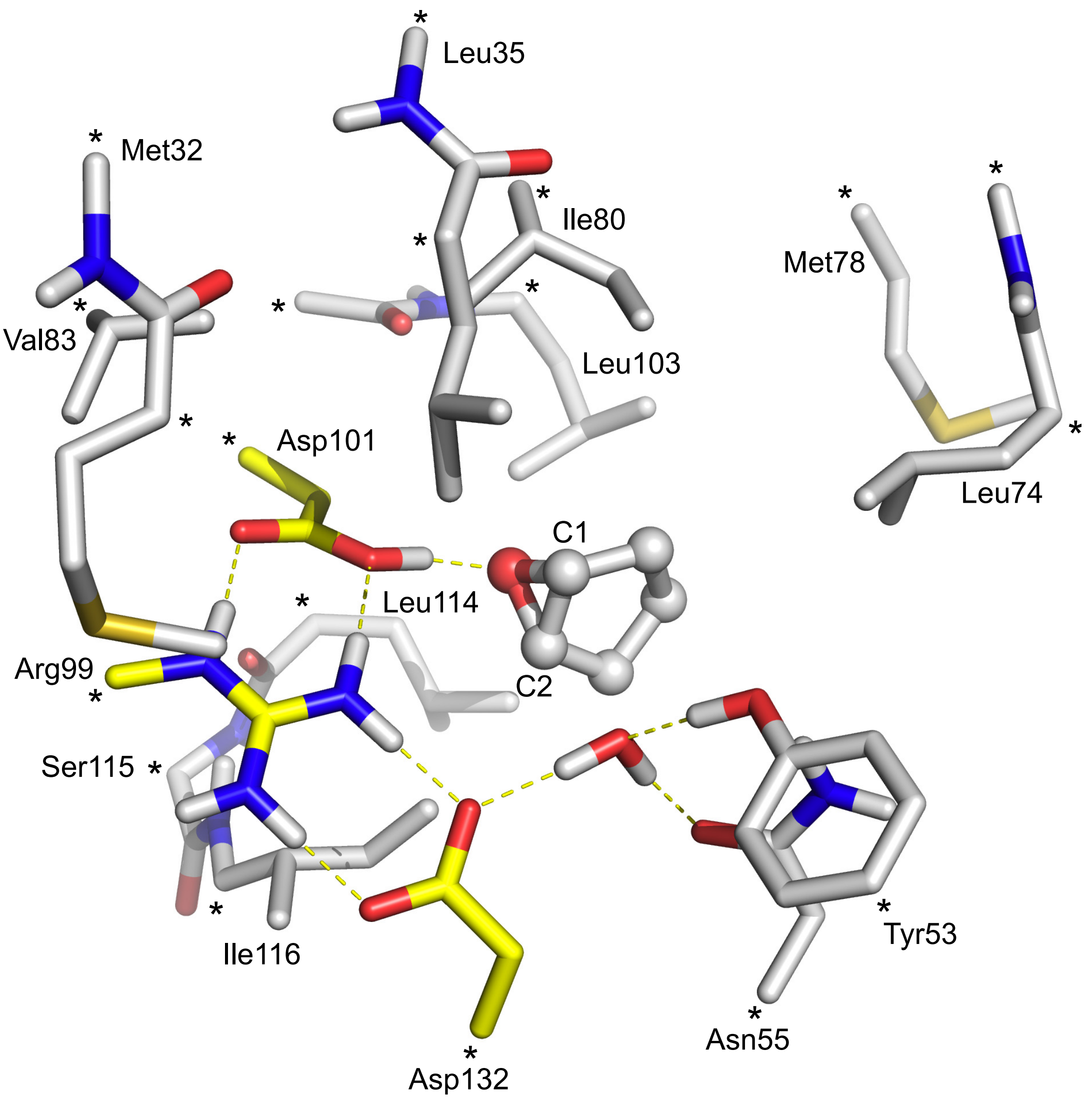

Asterisks indicate positions fixed to their crystallographic coordinates during the geometry optimizations. These apply to all structures except the variants containing the Leu114Cys mutation (*S1*, *S2* and *S3*) for which the fixing of the alpha carbon of Cys114 was released in order to grant flexibility to that side chain.
Lists of the fixed centers are given below along with the Cartesian coordinates.

4.1 Wild-type LEH

The following atoms were kept fixed in the geometry optimizations of reactant and transition state structures: 1, 9, 10, 18, 19, 26, 31, 39, 40, 45, 50, 54, 59, 64, 68, 73, 81, 85, 90.

Reactant:

C1	-6.209395	5.841235	0.951198
C2	-5.779370	6.770890	-0.172372
O3	-4.672634	6.652216	-0.700513
C4	-7.668097	5.864608	1.418069
C5	-7.957838	4.942917	2.612433
S6	-7.693121	3.141240	2.331207
C7	-6.038739	2.878250	3.070398
N8	-6.664569	7.735633	-0.535153
C9	-6.356473	8.718150	-1.549019
C10	-1.164015	6.921811	-0.384290
C11	-0.336914	7.128244	-1.644705
O12	0.880444	6.951471	-1.671559
C13	-0.997002	5.486688	0.148153
C14	-1.871058	5.148196	1.369476
C15	-1.749920	3.654262	1.708726
C16	-1.529321	6.007002	2.596217
N17	-1.043827	7.527615	-2.743663
C18	-0.416760	7.710332	-4.038680
C19	2.574957	1.070906	9.137398
C20	1.510002	0.396181	8.536035
C21	3.575919	1.615848	8.329447
C22	1.443450	0.252751	7.149879
C23	3.521589	1.482886	6.943122
C24	2.455029	0.797236	6.344606
O25	2.466406	0.655598	4.989101
C26	2.562819	-4.802401	6.846202
C27	3.635447	-4.314623	5.862188
C28	3.142574	-3.089782	5.107454
N29	3.886798	-1.966463	5.206758
O30	2.098355	-3.151028	4.443502
C31	9.033309	2.049470	1.721550
C32	9.142052	2.815591	0.425170
O33	9.691860	2.340970	-0.570387
C34	7.796028	2.278034	2.607248
C35	6.438827	1.907004	1.975784
C36	6.345671	0.409348	1.648916
C37	5.287792	2.333387	2.897897
N38	8.654952	4.080362	0.461718
C39	8.731458	4.950420	-0.679021
C40	8.355975	1.390059	-4.313563
C41	9.025879	0.125070	-3.763448
C42	8.470636	-0.248241	-2.387287
S43	9.276887	-1.685142	-1.570403
C44	10.912368	-0.960412	-1.175052
C45	1.943681	3.162884	-4.816808
C46	1.704442	3.542551	-3.348266
C47	3.021441	3.489329	-2.548493
C48	0.609687	2.654823	-2.736106
C49	2.919811	4.004458	-1.107634
C50	-5.488690	2.247291	-4.777059
C51	-5.664581	3.329032	-3.701981
C52	-6.530717	2.806834	-2.546965
C53	-4.306381	3.826862	-3.187454
C54	-6.780645	-0.492661	0.496621
N55	-5.337027	-0.430955	0.648310
C56	-4.669895	-0.773079	1.768377
N57	-5.323113	-1.144209	2.884658
N58	-3.341496	-0.693147	1.796677
C59	-2.962368	-1.371784	-4.328729
C60	-2.056813	-0.807419	-3.231754
C61	-2.804674	-0.378347	-1.985517
O62	-4.017658	-0.229838	-1.934625
O63	-2.075212	-0.135081	-0.887516
C64	-0.877495	-1.145320	-7.519621
C65	0.325420	-0.798054	-6.664255
O66	0.281780	0.031610	-5.758841
N67	1.452960	-1.484928	-7.002344
C68	2.769776	-1.198895	-6.460663
C69	3.427286	-2.387308	-5.720676
C70	3.288044	-2.387963	-4.181875
C71	4.098153	-1.247784	-3.544628
C72	1.824985	-2.361769	-3.716652
C73	-1.562657	-6.432758	-4.513316
C74	-2.837210	-6.408259	-3.681542
O75	-3.337710	-5.358826	-3.268998
C76	-0.342012	-5.946684	-3.707450
C77	0.089064	-6.863337	-2.544907
C78	0.590489	-8.232262	-3.029308
C79	1.162798	-6.170017	-1.693762
N80	-3.399295	-7.619821	-3.427209
C81	-4.736512	-7.747616	-2.871660
C82	-4.864455	-7.779011	-1.340233
O83	-5.202047	-8.805598	-0.758878
N84	-4.647429	-6.586086	-0.730741
C85	-4.970129	-6.379787	0.675526
C86	-4.063485	-5.355284	1.365461
C87	-2.603397	-5.844524	1.441775
C88	-4.648814	-5.035570	2.746813
C89	-1.580245	-4.715567	1.598502
C90	-2.338554	-4.513584	6.202729
C91	-1.775168	-3.131487	5.836846
C92	-2.594648	-2.357515	4.789139
O93	-3.845611	-2.480545	4.795971
O94	-1.947587	-1.613388	3.973492
O95	0.636962	-0.870166	3.966301
H96	-7.951706	6.878746	1.738335
H97	-8.330313	5.591848	0.587379
H98	-7.361157	5.235165	3.483093
H99	-9.009050	5.042997	2.897482

H100	-6.011961	3.258368	4.094641
H101	-5.878263	1.798179	3.100826
H102	-5.243712	3.337673	2.479901
H103	-5.945257	4.831641	0.621786
H104	-7.160186	8.780092	-2.289819
H105	-1.249906	4.787883	-0.659931
H106	0.060975	5.318225	0.383125
H107	-2.913362	5.351405	1.085212
H108	-2.399165	3.379161	2.547588
H109	-2.022199	3.025233	0.853601
H110	-0.721586	3.402275	1.996979
H111	-2.153863	5.733022	3.454035
H112	-1.680360	7.074510	2.407182
H113	-0.482831	5.865368	2.892956
H114	-0.797831	7.643206	0.355490
H115	-0.638064	6.881691	-4.723059
H116	0.720883	-0.029593	9.150098
H117	4.407191	2.151155	8.780728
H118	0.624613	-0.282651	6.679561
H119	4.290668	1.910227	6.306844
H120	1.637450	0.189121	4.661641
H121	3.836656	-5.094141	5.118679
H122	4.578407	-4.108594	6.380581
H123	1.630869	-5.003833	6.313767
H124	7.761724	3.322803	2.950929
H125	7.929310	1.688942	3.524588
H126	6.339486	2.466022	1.033905
H127	5.371241	0.172879	1.211911
H128	6.453874	-0.194320	2.558948
H129	7.110408	0.083025	0.937853
H130	5.328497	3.404516	3.126481
H131	4.314637	2.127623	2.442837
H132	5.320403	1.787180	3.847898
H133	9.125513	0.992580	1.460231
H134	7.743476	5.141854	-1.115296
H135	1.352070	4.583754	-3.315097
H136	0.350046	2.968963	-1.720598
H137	-0.304568	2.694798	-3.338955
H138	0.931468	1.606371	-2.695727
H139	3.401004	2.456089	-2.550995
H140	3.771447	4.087217	-3.083895
H141	2.486316	5.009740	-1.081272
H142	2.292831	3.355223	-0.486657
H143	3.908640	4.044704	-0.636329
H144	2.264038	2.118494	-4.906306
H145	-6.186816	4.182307	-4.160522
H146	-6.694510	3.577551	-1.785684
H147	-7.512242	2.471372	-2.901287
H148	-6.038774	1.955867	-2.060898
H149	-4.421331	4.621325	-2.442430
H150	-3.691326	4.217774	-4.006330
H151	-3.749329	3.006289	-2.719027
H152	-4.994628	1.366424	-4.350646
H153	-1.494246	0.058837	-3.603910
H154	-1.294197	-1.538398	-2.937778
H155	-3.737924	-0.654929	-4.607469
H156	-1.055528	-2.225798	-7.543298
H157	3.014466	-3.321165	-6.125304
H158	4.496784	-2.398427	-5.965532
H159	3.733951	-3.332222	-3.837099
H160	4.082646	-1.321986	-2.452732
H161	3.692662	-0.263805	-3.806078
H162	5.145830	-1.276334	-3.865475
H163	1.760366	-2.403061	-2.624506
H164	1.320122	-1.445941	-4.039451
H165	1.267015	-3.214293	-4.120659
H166	2.647438	-0.337096	-5.802527
H167	-0.582295	-4.953074	-3.312733
H168	0.505031	-5.817466	-4.394360
H169	-0.787657	-7.030712	-1.902408
H170	0.889028	-8.860007	-2.183614
H171	-0.171518	-8.785312	-3.588918
H172	1.463835	-8.118767	-3.683403
H173	1.459851	-6.794388	-0.844779
H174	0.799586	-5.215847	-1.298013
H175	2.062812	-5.965106	-2.286347
H176	-1.385622	-7.426281	-4.938559
H177	-5.335728	-6.915990	-3.258140
H178	-4.082155	-4.432503	0.762358
H179	-5.672665	-4.652457	2.661365
H180	-4.071678	-4.288110	3.292645
H181	-4.691624	-5.942185	3.363167
H182	-2.367942	-6.409731	0.531531
H183	-2.513028	-6.555323	2.274775
H184	-1.578656	-4.084447	0.701628
H185	-0.567653	-5.112030	1.726365
H186	-1.793228	-4.068561	2.454672
H187	-4.896771	-7.355431	1.164716
H188	-1.749212	-2.500760	6.737066
H189	-0.742502	-3.207938	5.482626
H190	-2.304436	-5.193843	5.345556
H191	8.758377	1.658211	-5.296175
H192	10.105543	0.297233	-3.685588
H193	10.786774	0.028144	-0.726568
H194	11.392861	-1.631510	-0.459282
H195	-7.164150	-1.522161	0.519842
H196	-7.289840	0.093343	1.269403
H197	-7.035180	-0.057837	-0.470134
H198	-2.812000	-0.995529	2.648169
C199	0.396745	-1.565229	0.772407
C200	0.795326	-0.160414	0.743097
O201	0.405241	-0.831281	-0.508292
C202	2.291395	-0.059281	0.921689
C203	1.619348	-2.434403	0.951447
C204	2.802741	-1.490397	0.617475
H205	0.132367	0.645139	1.045685
H206	2.746838	0.703712	0.282727
H207	3.701219	-1.739820	1.187064
H208	2.469190	0.216378	1.967042
H209	3.050621	-1.570833	-0.443290
H210	1.653039	-2.760718	1.995641
H211	1.599074	-3.328440	0.319674
H212	-0.586081	-1.884226	1.104458
H213	1.027759	3.262691	-5.408982
H214	8.519179	2.237136	-3.639055
H215	8.892078	-0.714614	-4.456163
H216	9.182110	5.912700	-0.411945
H217	9.355268	4.461993	-1.427284
H218	9.935109	2.290308	2.302411
H219	2.619818	1.176774	10.216892

H220	2.885661	-5.719942	7.346382
H221	2.360644	-4.049707	7.614690
H222	-1.761541	-4.966848	7.015358
H223	-3.381651	-4.426893	6.515998
H224	-6.018544	-6.059019	0.767631
H225	-5.166152	-8.686252	-3.224751
H226	-1.732389	-5.748918	-5.351855
H227	3.418895	-0.879692	-7.285990
H228	-0.720631	-0.810939	-8.551356
H229	-3.449165	-2.295921	-4.003595
H230	-2.852543	-0.377633	0.966486
H231	-4.790852	-0.263153	-0.201182
H232	-0.748902	8.645398	-4.501708
H233	0.660571	7.748891	-3.876430
H234	-6.202043	9.713904	-1.116302
H235	-5.437304	8.405150	-2.043449
H236	-5.542572	6.064197	1.795129
H237	7.273562	1.254557	-4.423001
H238	11.545348	-0.884823	-2.062525
H239	8.548842	0.605199	-1.708410
H240	7.413450	-0.527088	-2.462573
H241	-2.224620	7.131883	-0.558205
H242	-1.758561	-0.646106	-7.121474
H243	-6.454313	1.924184	-5.182473
H244	-4.877255	2.607593	-5.611983
H245	-2.364694	-1.598324	-5.211843
H246	2.713874	3.796066	-5.272493
H247	4.640917	-1.923160	5.873801
H248	3.502931	-1.076766	4.887457
H249	8.184712	4.399190	1.293451
H250	1.398040	-2.123407	-7.782453
H251	-2.050220	7.552558	-2.675707
H252	-7.549133	7.793061	-0.057065
H253	-4.765734	-1.594842	3.654013
H254	-6.286718	-1.425706	2.796102
H255	-4.394614	-5.798594	-1.319749
H256	-2.939935	-8.453395	-3.758968
H257	-1.102444	-0.392272	-0.935286
H258	-0.330237	-1.072203	4.053569
H259	1.095888	-1.727708	4.094198

TS attack at C1:

C1	-5.967270	6.114222	0.820263
C2	-5.529263	6.990567	-0.342367
O3	-4.429200	6.830262	-0.871366
C4	-7.421683	6.176057	1.297960
C5	-7.705055	5.334995	2.551984
S6	-7.453392	3.517006	2.393276
C7	-5.770400	3.300134	3.081089
N8	-6.397589	7.962122	-0.730053
C9	-6.081842	8.901657	-1.780974
C10	-0.922786	6.982156	-0.665305
C11	-0.122352	7.070872	-1.957018
O12	1.078965	6.810107	-2.008137
C13	-0.761890	5.608689	0.005247
C14	-1.567137	5.435069	1.306725
C15	-1.489916	3.977913	1.787079
C16	-1.108164	6.391556	2.417694
N17	-0.833424	7.488589	-3.046490
C18	-0.236187	7.612869	-4.361993
C19	2.850501	1.363386	8.981833
C20	1.693415	0.772466	8.468058
C21	3.872949	1.727515	8.102075
C22	1.555793	0.531995	7.099018
C23	3.748613	1.496198	6.733363
C24	2.590395	0.892402	6.227144
O25	2.546327	0.647504	4.880681
C26	2.600465	-4.585825	6.910642
C27	3.566326	-4.298631	5.748840
C28	3.138285	-3.043975	5.008237
N29	3.943140	-1.965407	5.108640
O30	2.078037	-3.033797	4.363064
C31	9.162441	1.862935	1.394174
C32	9.254241	2.581908	0.070770
O33	9.736506	2.041227	-0.926249
C34	8.080677	2.286574	2.401258
C35	6.620449	2.057975	1.960484
C36	6.312292	0.568462	1.751459
C37	5.658982	2.675272	2.986067
N38	8.838591	3.871470	0.078503
C39	8.896285	4.682992	-1.104987
C40	8.324712	1.006921	-4.595955
C41	8.667069	-0.310178	-3.888988
C42	8.020448	-0.379815	-2.504123
S43	8.479247	-1.839973	-1.483759
C44	10.250373	-1.481701	-1.177725
C45	1.961378	2.966338	-5.022978
C46	1.636270	3.359671	-3.574661
C47	2.764867	2.915845	-2.623107
C48	0.268231	2.791866	-3.167874
C49	2.625809	3.432737	-1.186267
C50	-5.493179	2.293165	-4.785407
C51	-5.563763	3.168784	-3.526185
C52	-6.408108	2.486240	-2.440414
C53	-4.160054	3.499756	-2.998779
C54	-6.747815	-0.210267	0.613380
N55	-5.304923	-0.322115	0.708713
C56	-4.624467	-0.535761	1.843525
N57	-5.250667	-0.616408	3.043499
N58	-3.298361	-0.620226	1.804993
C59	-3.072179	-1.386889	-4.259130
C60	-2.269127	-1.656042	-2.983011
C61	-2.815006	-0.976670	-1.727598
O62	-3.984144	-0.546721	-1.714155
O63	-2.042941	-0.940946	-0.686351
C64	-1.055893	-1.343905	-7.501432
C65	0.188783	-0.979219	-6.718703
O66	0.223244	-0.033370	-5.937462
N67	1.269126	-1.767127	-6.991046
C68	2.611501	-1.476670	-6.521922
C69	3.249014	-2.615628	-5.693817
C70	3.089724	-2.517197	-4.160235
C71	3.915823	-1.357159	-3.583379
C72	1.623474	-2.428172	-3.715077
C73	-1.836971	-6.493768	-4.286910
C74	-3.065370	-6.409524	-3.392625
O75	-3.485751	-5.338830	-2.947552

C76	-0.554629	-6.055279	-3.552986	H136	-0.021003	3.099141	-2.158229
C77	-0.116594	-6.964206	-2.387211	H137	-0.515363	3.133964	-3.852603
C78	0.271976	-8.375582	-2.853181	H138	0.272725	1.696048	-3.204074
C79	1.042853	-6.315096	-1.617360	H139	2.824156	1.817222	-2.620979
N80	-3.687607	-7.589522	-3.127879	H140	3.720649	3.270574	-3.032755
C81	-5.011616	-7.644992	-2.528059	H141	2.509633	4.521847	-1.173699
C82	-5.093728	-7.621971	-0.992253	H142	1.750348	3.005943	-0.684518
O83	-5.440592	-8.619564	-0.366656	H143	3.507642	3.176249	-0.586730
N84	-4.841206	-6.411847	-0.434094	H144	1.939484	1.878573	-5.150600
C85	-5.119652	-6.142376	0.970303	H145	-6.056409	4.114601	-3.798637
C86	-4.157126	-5.127199	1.592881	H146	-6.483850	3.105190	-1.538958
C87	-2.720115	-5.681431	1.651988	H147	-7.425951	2.283355	-2.794124
C88	-4.689637	-4.714284	2.971261	H148	-5.950292	1.531856	-2.154797
C89	-1.648884	-4.590736	1.695078	H149	-4.201652	4.208470	-2.164215
C90	-2.303025	-4.162524	6.365177	H150	-3.537090	3.947828	-3.781437
C91	-1.632584	-2.853613	5.922427	H151	-3.657142	2.589365	-2.651818
C92	-2.441514	-2.041011	4.896396	H152	-5.012115	1.334396	-4.559870
O93	-3.689865	-2.068258	4.951659	H153	-1.217379	-1.378058	-3.109291
O94	-1.777747	-1.355120	4.034286	H154	-2.283588	-2.733240	-2.764212
O95	0.676304	-0.821514	3.628210	H155	-2.938501	-0.355759	-4.599673
H96	-7.696004	7.210335	1.556408	H156	-1.124190	-2.415378	-7.714451
H97	-8.093650	5.855601	0.492633	H157	2.833621	-3.571367	-6.041673
H98	-7.100374	5.681792	3.396911	H158	4.321566	-2.651521	-5.923595
H99	-8.753580	5.458934	2.838007	H159	3.511900	-3.447295	-3.751995
H100	-5.705479	3.740454	4.079403	H160	3.870288	-1.356314	-2.489223
H101	-5.604570	2.223409	3.165609	H161	3.544284	-0.383832	-3.922406
H102	-5.001574	3.726140	2.433797	H162	4.969822	-1.436439	-3.873470
H103	-5.717256	5.088592	0.532289	H163	1.545103	-2.411024	-2.622941
H104	-6.843038	8.885653	-2.568454	H164	1.149671	-1.515575	-4.089826
H105	-1.084772	4.837618	-0.705768	H165	1.043778	-3.282193	-4.082793
H106	0.303315	5.429826	0.194478	H166	2.540686	-0.552215	-5.946529
H107	-2.616935	5.660432	1.069509	H167	-0.718764	-5.040252	-3.174951
H108	-2.083287	3.823542	2.695468	H168	0.262875	-5.990915	-4.283572
H109	-1.858792	3.284663	1.023397	H169	-0.965106	-7.057154	-1.693806
H110	-0.454503	3.699226	2.020550	H170	0.591661	-8.992160	-2.006851
H111	-1.684060	6.232137	3.336419	H171	-0.557625	-8.899995	-3.339211
H112	-1.226891	7.442232	2.134812	H172	1.102921	-8.336046	-3.568392
H113	-0.050365	6.229234	2.659254	H173	1.346184	-6.931471	-0.764521
H114	-0.534046	7.768491	-0.006888	H174	0.761948	-5.327555	-1.236665
H115	-0.528536	6.789985	-5.026058	H175	1.920360	-6.186685	-2.263018
H116	0.885373	0.489451	9.136833	H176	-1.725003	-7.497063	-4.711599
H117	4.775796	2.197696	8.482036	H177	-5.592124	-6.803617	-2.922867
H118	0.657472	0.064705	6.704939	H178	-4.150715	-4.233770	0.948043
H119	4.533782	1.783123	6.040961	H179	-5.695592	-4.286028	2.891489
H120	1.692273	0.237400	4.585132	H180	-4.063923	-3.968023	3.463398
H121	3.538528	-5.125074	5.031729	H181	-4.757270	-5.586235	3.633703
H122	4.595847	-4.209908	6.112542	H182	-2.544575	-6.308952	0.769790
H123	1.578926	-4.684446	6.536425	H183	-2.627513	-6.343567	2.523865
H124	8.217422	3.341039	2.684536	H184	-1.701010	-3.987963	0.780802
H125	8.253482	1.727000	3.330529	H185	-0.642748	-5.019206	1.761416
H126	6.465874	2.572028	1.000777	H186	-1.778865	-3.911395	2.543907
H127	5.272639	0.431730	1.440191	H187	-5.068743	-7.101015	1.495015
H128	6.453531	0.008489	2.685064	H188	-1.494311	-2.201398	6.796952
H129	6.943321	0.106908	0.986227	H189	-0.633532	-3.039983	5.514451
H130	5.819566	3.753930	3.093446	H190	-2.380495	-4.867165	5.531249
H131	4.613668	2.519352	2.702515	H191	8.790056	1.059635	-5.585865
H132	5.802640	2.219336	3.973484	H192	9.755589	-0.388626	-3.788005
H133	9.068526	0.799966	1.157857	H193	10.377624	-0.430146	-0.908445
H134	7.901051	4.859904	-1.531240	H194	10.566068	-2.116136	-0.346184
H135	1.579615	4.456113	-3.514285	H195	-7.262408	-1.111790	0.974834

H196	-7.133454	0.658858	1.159929
H197	-7.001083	-0.082650	-0.439225
H198	-2.773761	-0.854912	2.664257
C199	0.569282	-1.849175	0.885381
C200	0.702870	-0.496861	1.440279
O201	0.374056	-1.138044	-0.349694
C202	2.118514	-0.024442	1.317668
C203	1.952491	-2.475235	0.927878
C204	2.921376	-1.266822	0.831239
H205	-0.151305	0.145173	1.605486
H206	2.176236	0.817219	0.620723
H207	3.820274	-1.427645	1.429995
H208	2.446413	0.351342	2.290286
H209	3.238884	-1.113270	-0.201801
H210	2.070120	-3.004719	1.878386
H211	2.088376	-3.194052	0.115089
H212	-0.278733	-2.461100	1.183054
H213	1.221395	3.378075	-5.718407
H214	8.679395	1.861192	-4.009835
H215	8.345958	-1.168247	-4.491890
H216	9.357436	5.653479	-0.892635
H217	9.500992	4.155067	-1.841908
H218	10.148777	1.970143	1.866852
H219	2.948652	1.544544	10.047313
H220	2.875709	-5.514845	7.417443
H221	2.618163	-3.778019	7.648936
H222	-1.729982	-4.645287	7.163567
H223	-3.314827	-3.964580	6.726018
H224	-6.152632	-5.778857	1.075987
H225	-5.486712	-8.577072	-2.836914
H226	-2.021087	-5.809373	-5.122111
H227	3.246719	-1.263425	-7.391995
H228	-1.052855	-0.807631	-8.457305
H229	-4.136713	-1.551009	-4.078654
H230	-2.833340	-0.654275	0.886799
H231	-4.762099	-0.319097	-0.177629
H232	-0.523453	8.560356	-4.829948
H233	0.846132	7.586339	-4.232576
H234	-5.999173	9.924654	-1.395365
H235	-5.123337	8.606793	-2.207193
H236	-5.292080	6.364265	1.649964
H237	7.242728	1.119532	-4.730685
H238	10.865501	-1.716863	-2.049480
H239	8.271879	0.515853	-1.930572
H240	6.929639	-0.433942	-2.591859
H241	-1.984767	7.189751	-0.833634
H242	-1.934918	-1.029304	-6.940879
H243	-6.493199	2.079969	-5.180800
H244	-4.915193	2.779830	-5.579062
H245	-2.748049	-2.058316	-5.059320
H246	2.948320	3.335124	-5.327195
H247	4.729471	-1.979245	5.738682
H248	3.599028	-1.054092	4.807408
H249	8.408056	4.243203	0.909422
H250	1.160453	-2.499505	-7.676761
H251	-1.834107	7.575473	-2.951006
H252	-7.286375	8.038054	-0.262738
H253	-4.717560	-1.073510	3.807457
H254	-6.246179	-0.775138	3.036915
H255	-4.563293	-5.658481	-1.056397
H256	-3.297816	-8.442188	-3.497691
H257	-0.684006	-1.076704	-0.575914
H258	-0.296137	-1.043858	3.872307
H259	1.180157	-1.652548	3.814824

TS attack at C2:

C1	6.090142	-6.016298	0.687406
C2	5.644962	-6.881880	-0.480922
O3	4.547195	-6.709424	-1.011058
C4	7.551144	-6.073973	1.145721
C5	7.846887	-5.242375	2.403112
S6	7.583234	-3.424574	2.264087
C7	5.909771	-3.224137	2.979402
N8	6.506697	-7.857531	-0.873553
C9	6.184521	-8.791932	-1.927226
C10	1.027218	-6.936256	-0.700945
C11	0.199653	-7.057388	-1.972507
O12	-1.013328	-6.851354	-1.992158
C13	0.874297	-5.543965	-0.065874
C14	1.710110	-5.329510	1.209888
C15	1.637330	-3.859591	1.650403
C16	1.283356	-6.256139	2.358419
N17	0.900177	-7.432358	-3.083900
C18	0.274147	-7.560207	-4.385818
C19	-2.616454	-1.399376	9.042884
C20	-1.537095	-0.709048	8.484658
C21	-3.590808	-1.927864	8.191463
C22	-1.433070	-0.527949	7.102634
C23	-3.498612	-1.758399	6.810692
C24	-2.421827	-1.050450	6.260305
O25	-2.426543	-0.863166	4.902037
C26	-2.475732	4.560451	6.992007
C27	-3.646093	3.877688	6.281524
C28	-3.166411	2.706189	5.441285
N29	-3.995730	1.651106	5.308713
O30	-2.062311	2.761071	4.876877
C31	-9.072602	-1.941430	1.580509
C32	-9.158828	-2.641943	0.245199
O33	-9.593423	-2.081229	-0.761797
C34	-7.891776	-2.285327	2.506662
C35	-6.482536	-2.044475	1.927958
C36	-6.267255	-0.584447	1.504794
C37	-5.414815	-2.481047	2.941224
N38	-8.786383	-3.945864	0.256715
C39	-8.824269	-4.748345	-0.935227
C40	-8.364524	-1.052184	-4.421496
C41	-9.023573	0.202963	-3.837063
C42	-8.405997	0.583273	-2.489517
S43	-9.191946	2.006553	-1.630502
C44	-10.813291	1.272844	-1.196189
C45	-1.989050	-2.936656	-4.983445
C46	-1.733996	-3.365623	-3.531135
C47	-2.999119	-3.170365	-2.671799
C48	-0.517948	-2.618296	-2.964209
C49	-2.902068	-3.743515	-1.252723
C50	5.460599	-2.178819	-4.891681
C51	5.552344	-3.040691	-3.624395

C52	6.452463	-2.366777	-2.578820
C53	4.159972	-3.324455	-3.041504
C54	6.794193	0.317511	0.491446
N55	5.351018	0.380253	0.627352
C56	4.694551	0.563159	1.781182
N57	5.348593	0.664751	2.965022
N58	3.364982	0.591653	1.780562
C59	3.008769	1.471066	-4.301881
C60	2.272654	1.747952	-2.987294
C61	2.851128	1.042225	-1.760747
O62	4.016156	0.600215	-1.786937
O63	2.110181	1.000573	-0.697629
C64	0.928736	1.417731	-7.503504
C65	-0.282028	1.074547	-6.661037
O66	-0.259285	0.193559	-5.806887
N67	-1.394362	1.807219	-6.959805
C68	-2.719636	1.504476	-6.450548
C69	-3.351501	2.627548	-5.593832
C70	-3.191822	2.493531	-4.062548
C71	-4.012612	1.317602	-3.509975
C72	-1.724760	2.401252	-3.621798
C73	1.715070	6.563487	-4.283689
C74	2.972504	6.489212	-3.428497
O75	3.430450	5.421255	-3.015823
C76	0.500513	5.922130	-3.589550
C77	0.008638	6.645445	-2.319089
C78	-0.538426	8.050225	-2.615123
C79	-1.050569	5.796058	-1.601588
N80	3.583699	7.677264	-3.173985
C81	4.910876	7.744150	-2.583662
C82	4.976033	7.719620	-1.048780
O83	5.136095	8.750926	-0.402788
N84	4.907724	6.475148	-0.514210
C85	5.105913	6.229065	0.905541
C86	4.195970	5.129937	1.469687
C87	2.712743	5.522363	1.345349
C88	4.625095	4.834769	2.913428
C89	1.732045	4.368412	1.564502
C90	2.420393	4.195678	6.347155
C91	1.756660	2.876960	5.919965
C92	2.541518	2.068336	4.870714
O93	3.790471	2.119063	4.879881
O94	1.859719	1.353735	4.045241
O95	-0.614184	0.846958	3.657296
H96	7.835106	-7.108707	1.392096
H97	8.210448	-5.743631	0.334074
H98	7.255377	-5.599707	3.252950
H99	8.899779	-5.363400	2.673996
H100	5.862551	-3.674508	3.974250
H101	5.739224	-2.149300	3.076842
H102	5.133740	-3.648445	2.339578
H103	5.829747	-4.989291	0.414050
H104	6.980288	-8.825892	-2.678597
H105	1.174878	-4.791149	-0.805846
H106	-0.187389	-5.366975	0.144690
H107	2.754740	-5.556795	0.952595
H108	2.251349	-3.676403	2.539408
H109	1.985236	-3.186913	0.859040
H110	0.606648	-3.578285	1.900960
H111	1.882117	-6.069267	3.257142
H112	1.398529	-7.314115	2.102278
H113	0.231630	-6.090150	2.622795
H114	0.654597	-7.704892	-0.013592
H115	0.508797	-6.711663	-5.040527
H116	-0.763954	-0.298577	9.128181
H117	-4.431411	-2.479613	8.603280
H118	-0.596991	0.020237	6.677227
H119	-4.247455	-2.172856	6.142897
H120	-1.607644	-0.423966	4.570686
H121	-4.137796	4.584807	5.600437
H122	-4.410011	3.549390	6.995335
H123	-1.720495	4.867085	6.266763
H124	-7.965689	-3.329084	2.846964
H125	-8.003122	-1.685435	3.420181
H126	-6.370854	-2.672024	1.032175
H127	-5.252511	-0.447977	1.118904
H128	-6.393502	0.093587	2.358749
H129	-6.961270	-0.267130	0.721427
H130	-5.538425	-3.530585	3.231483
H131	-4.405425	-2.365121	2.535328
H132	-5.471195	-1.874363	3.853434
H133	-9.087746	-0.870655	1.363951
H134	-7.823388	-4.915397	-1.352402
H135	-1.505993	-4.441354	-3.521564
H136	-0.273541	-2.942894	-1.948221
H137	0.368507	-2.783745	-3.585672
H138	-0.699189	-1.536837	-2.945912
H139	-3.242422	-2.097649	-2.627350
H140	-3.843123	-3.648919	-3.187079
H141	-2.612746	-4.799490	-1.278009
H142	-2.157547	-3.213004	-0.649217
H143	-3.862098	-3.661834	-0.729315
H144	-2.155575	-1.855876	-5.052476
H145	6.008366	-4.003410	-3.901066
H146	6.526473	-2.964597	-1.663135
H147	7.468123	-2.213753	-2.962702
H148	6.039628	-1.387468	-2.309769
H149	4.211855	-4.044106	-2.216927
H150	3.488331	-3.740465	-3.801324
H151	3.708778	-2.399249	-2.664078
H152	5.006388	-1.207738	-4.663041
H153	1.209183	1.500065	-3.069449
H154	2.327720	2.821814	-2.758879
H155	2.808052	0.457097	-4.660131
H156	0.919893	2.450384	-7.866237
H157	-2.931369	3.588589	-5.919440
H158	-4.424170	2.672811	-5.821914
H159	-3.617643	3.412995	-3.633931
H160	-3.963739	1.289530	-2.416149
H161	-3.638360	0.353760	-3.872906
H162	-5.068117	1.397233	-3.795228
H163	-1.640332	2.361370	-2.530933
H164	-1.246107	1.501964	-4.020999
H165	-1.151599	3.266809	-3.971619
H166	-2.624515	0.576298	-5.884236
H167	0.769936	4.891986	-3.333590
H168	-0.325815	5.863506	-4.310629
H169	0.865955	6.751096	-1.638307
H170	-0.885415	8.536175	-1.697311
H171	0.214474	8.707502	-3.062818

H172	-1.388825	7.999918	-3.306425
H173	-1.387960	6.284095	-0.680635
H174	-0.658230	4.807848	-1.340281
H175	-1.930828	5.646370	-2.238686
H176	1.501299	7.596149	-4.579854
H177	5.496892	6.912167	-2.987468
H178	4.357990	4.219776	0.869836
H179	5.704823	4.650057	2.964947
H180	4.138324	3.958253	3.344381
H181	4.413434	5.693577	3.562570
H182	2.544519	5.940394	0.345552
H183	2.502755	6.333652	2.056624
H184	1.900624	3.569949	0.832490
H185	0.698545	4.714859	1.452007
H186	1.830540	3.933311	2.564009
H187	4.934198	7.179870	1.419201
H188	1.653169	2.222101	6.797472
H189	0.741655	3.049971	5.547232
H190	2.463555	4.903728	5.513677
H191	-8.810358	-1.323246	-5.384331
H192	-10.095404	0.015201	-3.706035
H193	-10.673917	0.272078	-0.779986
H194	-11.267061	1.924105	-0.445470
H195	7.288689	1.231674	0.849098
H196	7.222604	-0.544260	1.017366
H197	7.021585	0.209954	-0.569252
H198	2.856226	0.810212	2.651830
C199	-0.504699	1.612998	1.614806
C200	-0.649120	0.417502	0.769998
O201	-0.288940	1.308281	-0.299260
C202	-2.135770	0.106456	0.728285
C203	-1.773237	2.412804	1.574635
C204	-2.815914	1.493404	0.875230
H205	0.039682	-0.418268	0.898318
H206	-2.412563	-0.414217	-0.192003
H207	-3.743927	1.432002	1.448249
H208	-2.373603	-0.544375	1.576867
H209	-3.064476	1.898748	-0.107451
H210	-2.058848	2.707725	2.586266
H211	-1.596897	3.340434	1.021724
H212	0.462983	2.001851	1.898733
H213	-1.125949	-3.160625	-5.619548
H214	-8.485636	-1.901774	-3.741353
H215	-8.935808	1.045399	-4.533729
H216	-9.281247	-5.722864	-0.734158
H217	-9.423904	-4.219085	-1.675415
H218	-10.011454	-2.151702	2.111439
H219	-2.689461	-1.532166	10.117377
H220	-2.819881	5.443372	7.537729
H221	-2.000014	3.882647	7.706679
H222	1.862061	4.666509	7.162636
H223	3.445005	4.012696	6.678697
H224	6.155642	5.955348	1.085745
H225	5.372536	8.684045	-2.890479
H226	1.937834	6.008775	-5.202555
H227	-3.374524	1.291922	-7.305424
H228	0.958932	0.752807	-8.374152
H229	4.086764	1.578966	-4.162260
H230	2.879239	0.636820	0.873849
H231	4.789679	0.367277	-0.248147
H232	0.594304	-8.483357	-4.880144
H233	-0.804252	-7.588826	-4.227799
H234	6.027948	-9.804969	-1.537400
H235	5.262950	-8.451291	-2.398659
H236	5.427256	-6.280412	1.522790
H237	-7.290516	-0.902871	-4.582593
H238	-11.477956	1.220683	-2.061893
H239	-8.434227	-0.273419	-1.810552
H240	-7.359049	0.880713	-2.617755
H241	2.086238	-7.144371	-0.887694
H242	1.833899	1.246819	-6.922470
H243	6.451740	-1.991676	-5.321239
H244	4.847611	-2.662759	-5.660404
H245	2.686402	2.178450	-5.071205
H246	-2.862192	-3.448299	-5.405570
H247	-4.807458	1.571125	5.901018
H248	-3.629717	0.793151	4.898245
H249	-8.389072	-4.333210	1.097483
H250	-1.322688	2.491735	-7.697568
H251	1.905925	-7.476586	-3.015249
H252	7.389530	-7.949422	-0.397978
H253	4.819900	1.113894	3.736204
H254	6.337045	0.860698	2.931561
H255	4.693060	5.707474	-1.142186
H256	3.154558	8.532373	-3.490360
H257	0.754912	1.212256	-0.543834
H258	0.359440	1.019776	3.921154
H259	-1.131331	1.560682	4.104655

4.2 Mutant *R1*

The following atoms were kept fixed in the geometry optimizations of reactant and transition state structures: 1, 9, 10, 18, 19, 26, 31, 37, 38, 43, 45, 49, 54, 59, 63, 68, 76, 80, 85.

Reactant:

C1	-6.258881	5.777116	0.974202
C2	-5.858379	6.691173	-0.173524
O3	-4.766554	6.566087	-0.730519
C4	-7.726234	5.736003	1.412951
C5	-7.991970	4.838092	2.630877
S6	-7.641100	3.042288	2.413747
C7	-5.986213	2.877706	3.180421
N8	-6.749385	7.659169	-0.514080
C9	-6.470399	8.638736	-1.538913
C10	-1.244338	6.898296	-0.441546
C11	-0.439849	7.109309	-1.712996
O12	0.785688	6.981962	-1.747177
C13	-1.001308	5.495567	0.144679
C14	-1.833403	5.180809	1.401636
C15	-1.670194	3.703261	1.789642
C16	-1.477395	6.090428	2.587313
N17	-1.167916	7.460676	-2.811604
C18	-0.558638	7.673823	-4.110754
C19	2.690561	1.135573	9.054937
C20	1.644449	0.390278	8.505117
C21	3.607784	1.749244	8.198663
C22	1.517522	0.241692	7.123342
C23	3.491582	1.612851	6.816412
C24	2.448143	0.852345	6.268991
O25	2.408640	0.703645	4.915312
C26	2.700993	-4.750069	6.795608
C27	3.756253	-4.223213	5.812985
C28	3.213775	-3.030217	5.039692
N29	3.906757	-1.873394	5.133455
O30	2.183486	-3.147720	4.362045
C31	9.029198	2.134820	1.539226
C32	9.116879	2.872465	0.222033
O33	9.505637	2.348181	-0.822140
C34	7.593900	1.942823	2.083767
C35	6.709304	1.207172	1.068386
N36	8.735690	4.176272	0.294171
C37	8.664171	5.019548	-0.872096
C38	8.269321	1.435883	-4.481593
C39	8.949350	0.160076	-3.970574
C40	8.354887	-0.295400	-2.636163
S41	9.181083	-1.741893	-1.857090
C42	10.786492	-0.986272	-1.401155
C43	1.833590	3.144834	-4.899389
C44	1.521995	4.264649	-3.911771
C45	-5.588261	2.158807	-4.744791
C46	-5.704243	3.160331	-3.586829
C47	-6.642749	2.624894	-2.496338
C48	-4.326064	3.502994	-3.003042
C49	-6.775981	-0.564327	0.562000
N50	-5.330876	-0.565079	0.709518
C51	-4.670490	-0.821459	1.854228
N52	-5.329084	-1.097368	2.997304
N53	-3.341968	-0.754300	1.881025
C54	-3.021001	-1.433554	-4.314542
C55	-2.082737	-0.873454	-3.240531
C56	-2.787105	-0.570566	-1.933034
O57	-3.998574	-0.426787	-1.837938
O58	-2.032062	-0.436123	-0.837110
C59	-0.985665	-1.204783	-7.537094
C60	0.235907	-0.801662	-6.738093
O61	0.192692	0.031277	-5.834796
N62	1.366802	-1.464613	-7.102386
C63	2.677169	-1.217843	-6.531940
C64	3.274536	-2.414045	-5.749728
C65	3.162195	-2.342111	-4.210339
C66	4.031294	-1.214057	-3.633027
C67	1.709448	-2.229421	-3.726599
C68	-1.575673	-6.481922	-4.492750
C69	-2.851518	-6.463310	-3.662472
O70	-3.379440	-5.414524	-3.284180
C71	-0.391301	-5.844889	-3.742100
C72	0.126560	-6.642327	-2.527666
C73	0.756859	-7.983752	-2.931659
C74	1.129152	-5.799664	-1.726139
N75	-3.388350	-7.680221	-3.380606
C76	-4.712220	-7.817883	-2.797312
C77	-4.780703	-7.842221	-1.263830
O78	-4.935119	-8.894028	-0.650807
N79	-4.708942	-6.618279	-0.684652
C80	-4.906630	-6.432893	0.745578
C81	-4.106396	-5.256560	1.319623
C82	-2.588419	-5.478011	1.176010
C83	-4.536977	-5.032482	2.775037
C84	-1.751308	-4.204541	1.330974
C85	-2.211880	-4.511432	6.223184
C86	-1.747971	-3.071028	5.956446
C87	-2.578824	-2.318197	4.903142
O88	-3.827619	-2.458683	4.908669
O89	-1.941200	-1.571988	4.082333
O90	0.633001	-0.879797	3.928969
H91	-8.070995	6.741805	1.697824
H92	-8.356003	5.404694	0.578634
H93	-7.423640	5.184207	3.500720
H94	-9.051187	4.898731	2.896921
H95	-5.990150	3.291458	4.191899
H96	-5.779352	1.807096	3.247191
H97	-5.204605	3.353329	2.584618
H98	-5.933518	4.773316	0.685596
H99	-7.256246	8.647028	-2.301733
H100	-1.241916	4.750854	-0.625106
H101	0.067478	5.379738	0.361456
H102	-2.888313	5.350855	1.141914
H103	-2.281648	3.448024	2.662266
H104	-1.964342	3.040386	0.968289

H105	-0.627161	3.478735	2.044147
H106	-2.074754	5.836036	3.469978
H107	-1.653857	7.147811	2.365525
H108	-0.421299	5.978582	2.861631
H109	-0.912664	7.664411	0.268991
H110	-0.714333	6.821214	-4.783358
H111	0.918118	-0.088223	9.156784
H112	4.421743	2.341575	8.608549
H113	0.712489	-0.347523	6.695258
H114	4.197244	2.089592	6.144370
H115	1.588214	0.196204	4.615918
H116	3.999836	-5.000636	5.080018
H117	4.684357	-3.967550	6.336206
H118	1.781695	-4.996738	6.260007
H119	7.154021	2.937633	2.258635
H120	6.651185	1.750053	0.120224
H121	9.495797	1.157040	1.388636
H122	7.631718	5.167053	-1.212531
H123	1.785003	5.237847	-4.333435
H124	1.528726	2.166161	-4.518988
H125	-6.142122	4.087570	-3.986155
H126	-6.756348	3.341332	-1.675212
H127	-7.640657	2.409415	-2.895278
H128	-6.241734	1.695806	-2.073540
H129	-4.398879	4.273863	-2.228396
H130	-3.646370	3.871571	-3.780056
H131	-3.868077	2.612752	-2.555268
H132	-5.173143	1.207400	-4.391856
H133	-1.613984	0.056304	-3.588798
H134	-1.251685	-1.558704	-3.039738
H135	-3.913390	-0.811134	-4.406762
H136	-1.365206	-2.172097	-7.190311
H137	2.796368	-3.336087	-6.106793
H138	4.336738	-2.502914	-6.009353
H139	3.571459	-3.290619	-3.832923
H140	4.041714	-1.253374	-2.539381
H141	3.656521	-0.223705	-3.914610
H142	5.068395	-1.293529	-3.978926
H143	1.662775	-2.193656	-2.633501
H144	1.236948	-1.316865	-4.101977
H145	1.112969	-3.084240	-4.065587
H146	2.571300	-0.336880	-5.896582
H147	-0.710088	-4.850036	-3.411299
H148	0.435348	-5.694162	-4.449222
H149	-0.730145	-6.853600	-1.870847
H150	1.113319	-8.528070	-2.051230
H151	0.052440	-8.640091	-3.453770
H152	1.616336	-7.825620	-3.594809
H153	1.483974	-6.341949	-0.843619
H154	0.678678	-4.861544	-1.386071
H155	2.005866	-5.547249	-2.335207
H156	-1.334231	-7.496753	-4.826132
H157	-5.326186	-6.995624	-3.178109
H158	-4.372831	-4.357492	0.739074
H159	-5.614232	-4.840468	2.842140
H160	-4.028913	-4.189165	3.245079
H161	-4.327114	-5.924896	3.377458
H162	-2.386387	-5.912791	0.189388
H163	-2.269069	-6.228655	1.912139
H164	-2.016396	-3.473756	0.557378
H165	-0.684786	-4.432231	1.226314
H166	-1.890495	-3.720920	2.301386
H167	-4.622030	-7.370265	1.233435
H168	-1.823100	-2.490141	6.886474
H169	-0.696861	-3.048670	5.653139
H170	-2.076048	-5.140115	5.337088
H171	8.699733	1.761771	-5.434550
H172	10.020767	0.353183	-3.843950
H173	10.622056	-0.001783	-0.955950
H174	11.256662	-1.647664	-0.669701
H175	-7.219419	-1.541389	0.796971
H176	-7.251898	0.201430	1.185485
H177	-7.003048	-0.337519	-0.479947
H178	-2.817698	-1.024444	2.745728
C179	0.755971	-1.415453	0.726814
C180	0.748681	0.045001	0.738482
O181	0.540622	-0.676620	-0.529692
C182	2.159721	0.557393	0.904681
C183	2.174365	-1.914986	0.867761
C184	3.043388	-0.668454	0.557915
H185	-0.109629	0.625026	1.064116
H186	2.370210	1.426763	0.273975
H187	3.984110	-0.675209	1.113468
H188	2.277987	0.857379	1.952646
H189	3.290179	-0.645450	-0.505855
H190	2.306033	-2.260871	1.898506
H191	2.391763	-2.758124	0.203253
H192	-0.092377	-2.010979	1.045999
H193	1.291024	3.309555	-5.836421
H194	8.389655	2.249847	-3.759204
H195	8.862024	-0.647014	-4.708112
H196	9.107758	5.999934	-0.670367
H197	9.225673	4.531390	-1.668647
H198	9.628740	2.670980	2.287174
H199	2.783059	1.243667	10.131177
H200	3.061495	-5.648007	7.305514
H201	2.462053	-3.999969	7.555948
H202	-1.642843	-4.959482	7.044093
H203	-3.273526	-4.524009	6.481742
H204	-5.976062	-6.281923	0.953415
H205	-5.138846	-8.764430	-3.133672
H206	-1.789356	-5.892417	-5.391457
H207	3.356249	-0.941344	-7.347999
H208	-0.764353	-1.296890	-8.605464
H209	-3.336369	-2.452484	-4.070926
H210	-2.842417	-0.577985	1.017328
H211	-4.779163	-0.423433	-0.141790
H212	-0.967279	8.572387	-4.583724
H213	0.512527	7.802912	-3.954281
H214	-6.380479	9.647281	-1.118573
H215	-5.524184	8.365750	-2.005155
H216	-5.623803	6.065867	1.823114
H217	7.194821	1.283120	-4.636541
H218	11.448904	-0.891523	-2.265034
H219	8.376713	0.526380	-1.915388
H220	7.311644	-0.605144	-2.764945
H221	-2.316146	7.045686	-0.612549
H222	-1.765894	-0.456760	-7.397670
H223	-6.567059	1.948557	-5.190882
H224	-4.932877	2.537373	-5.536927

H225	-2.506129	-1.445375	-5.275222
H226	2.903266	3.109955	-5.130656
H227	4.646702	-1.786706	5.812048
H228	3.483257	-1.004065	4.808917
H229	8.369456	4.522101	1.167693
H230	1.302982	-2.125123	-7.862792
H231	-2.173958	7.460380	-2.732583
H232	-7.624426	7.713412	-0.018392
H233	-4.785365	-1.551066	3.771409
H234	-6.305178	-1.338895	2.928466
H235	-4.540389	-5.819745	-1.286815
H236	-2.900137	-8.514479	-3.666015
H237	-1.037294	-0.571474	-0.938943
H238	-0.322503	-1.112269	4.066895
H239	1.132063	-1.719665	3.983281
C240	7.653575	1.227087	3.447870
H241	8.029786	0.205731	3.296994
H242	8.397383	1.734274	4.077197
H243	7.109373	0.209064	0.854819
S244	2.478981	4.046238	-2.345369
H245	2.026952	5.178330	-1.761268
H246	0.456383	4.276123	-3.667852
C247	6.320539	1.183414	4.200502
H248	5.915612	2.192183	4.340630
H249	5.562049	0.597744	3.673505
H250	6.447582	0.740561	5.193854
H251	5.687874	1.092866	1.440501

TS attack at C1:

C1	-6.229512	5.808539	1.072998
C2	-5.823541	6.746953	-0.052304
O3	-4.724809	6.637651	-0.598113
C4	-7.679528	5.815123	1.566315
C5	-7.931912	4.904246	2.777655
S6	-7.639324	3.103068	2.520641
C7	-5.958582	2.887061	3.214453
N8	-6.718755	7.713545	-0.386763
C9	-6.437490	8.710878	-1.393280
C10	-1.214669	6.935662	-0.336959
C11	-0.411537	7.164335	-1.606608
O12	0.814514	7.040957	-1.643468
C13	-0.966033	5.530156	0.238886
C14	-1.799141	5.204622	1.492507
C15	-1.622590	3.728101	1.878124
C16	-1.458003	6.115713	2.681346
N17	-1.141526	7.532288	-2.698074
C18	-0.535252	7.767793	-3.994918
C19	2.723811	1.007969	9.055933
C20	1.655470	0.278211	8.529473
C21	3.649984	1.582914	8.182552
C22	1.515685	0.104381	7.151069
C23	3.521057	1.421195	6.804750
C24	2.456501	0.674475	6.285076
O25	2.416364	0.497637	4.928180
C26	2.708979	-4.840593	6.702306
C27	3.594242	-4.495097	5.491081
C28	3.127331	-3.207206	4.832945
N29	3.876600	-2.104962	5.048465
O30	2.090301	-3.190651	4.151482
C31	9.047556	2.106840	1.541582
C32	9.128031	2.863966	0.235239
O33	9.500541	2.351563	-0.820551
C34	7.611946	1.861744	2.063955
C35	6.761815	1.116296	1.026308
N36	8.754574	4.168149	0.330024
C37	8.686394	5.031157	-0.822165
C38	8.270756	1.507315	-4.487791
C39	8.906733	0.206744	-3.982122
C40	8.288752	-0.237862	-2.654677
S41	9.061134	-1.714144	-1.876063
C42	10.692365	-1.019077	-1.415390
C43	1.839815	3.244142	-4.862143
C44	1.511903	4.330690	-3.843259
C45	-5.584913	2.280429	-4.705039
C46	-5.682249	3.165043	-3.453682
C47	-6.659099	2.559763	-2.435737
C48	-4.301631	3.384589	-2.818178
C49	-6.768931	-0.523704	0.560235
N50	-5.326172	-0.643400	0.637898
C51	-4.637705	-0.872745	1.764189
N52	-5.259599	-1.008426	2.961816
N53	-3.310020	-0.922445	1.721673
C54	-3.028714	-1.326897	-4.338923
C55	-2.089974	-0.897740	-3.203822
C56	-2.768653	-0.778418	-1.839645
O57	-3.992864	-0.551749	-1.781675
O58	-2.027322	-0.905641	-0.783714
C59	-1.000430	-1.053115	-7.562411
C60	0.245420	-0.606898	-6.823620
O61	0.263602	0.367644	-6.077201
N62	1.331745	-1.399409	-7.051539
C63	2.664763	-1.094506	-6.566666
C64	3.286747	-2.190851	-5.673926
C65	3.075971	-2.034958	-4.151943
C66	3.853843	-0.833111	-3.593807
C67	1.594916	-1.964220	-3.757557
C68	-1.600808	-6.376523	-4.601803
C69	-2.872576	-6.372981	-3.764526
O70	-3.391933	-5.332428	-3.356650
C71	-0.425053	-5.705586	-3.868990
C72	0.114866	-6.475764	-2.646653
C73	0.767490	-7.811831	-3.032893
C74	1.106043	-5.602599	-1.864036
N75	-3.416198	-7.593758	-3.509331
C76	-4.737715	-7.729136	-2.920292
C77	-4.791367	-7.783520	-1.386933
O78	-4.929493	-8.848360	-0.792197
N79	-4.730684	-6.568149	-0.788396
C80	-4.918887	-6.400643	0.644865
C81	-4.043602	-5.292538	1.240998
C82	-2.547344	-5.639015	1.123934
C83	-4.481373	-5.036629	2.688689
C84	-1.615543	-4.432649	1.258774
C85	-2.204435	-4.576457	6.145952
C86	-1.590621	-3.218365	5.774525
C87	-2.432815	-2.390212	4.788451
O88	-3.679144	-2.471349	4.839435

O89	-1.798402	-1.638077	3.960364
O90	0.626002	-0.957281	3.591023
H91	-7.972886	6.828167	1.881236
H92	-8.353052	5.522595	0.751671
H93	-7.325260	5.220380	3.633023
H94	-8.979580	4.990216	3.080084
H95	-5.915382	3.267124	4.238303
H96	-5.764199	1.811971	3.235628
H97	-5.194598	3.371459	2.603084
H98	-5.960843	4.802069	0.737592
H99	-7.206696	8.714610	-2.173030
H100	-1.202308	4.789199	-0.535533
H101	0.103166	5.417954	0.456558
H102	-2.854462	5.364202	1.228445
H103	-2.234663	3.465891	2.748442
H104	-1.906999	3.063874	1.055012
H105	-0.577184	3.515469	2.135321
H106	-2.054337	5.851398	3.561929
H107	-1.647785	7.171102	2.461727
H108	-0.400873	6.017715	2.958011
H109	-0.885757	7.697575	0.379689
H110	-0.692587	6.926731	-4.681287
H111	0.921391	-0.167531	9.194909
H112	4.480092	2.163997	8.574808
H113	0.688471	-0.471583	6.745351
H114	4.230159	1.868230	6.116314
H115	1.589092	0.044019	4.615827
H116	3.519186	-5.287070	4.740209
H117	4.644983	-4.419863	5.792262
H118	1.664452	-4.933962	6.395106
H119	7.140711	2.839828	2.250357
H120	6.695092	1.674760	0.088052
H121	9.545861	1.145906	1.383910
H122	7.653130	5.204133	-1.147280
H123	1.740986	5.321498	-4.242849
H124	1.579783	2.248065	-4.495042
H125	-6.074866	4.145862	-3.762289
H126	-6.755472	3.190853	-1.544888
H127	-7.659384	2.429897	-2.865114
H128	-6.300596	1.576303	-2.109516
H129	-4.353880	4.096303	-1.986648
H130	-3.586845	3.779340	-3.549474
H131	-3.902714	2.436862	-2.437553
H132	-5.208347	1.285314	-4.442567
H133	-1.646474	0.080556	-3.433953
H134	-1.246763	-1.588797	-3.109655
H135	-3.893692	-0.662990	-4.394446
H136	-1.356228	-2.015141	-7.179078
H137	2.898902	-3.166262	-5.998715
H138	4.366778	-2.216601	-5.867486
H139	3.505649	-2.938666	-3.694589
H140	3.784134	-0.800347	-2.501732
H141	3.459511	0.118248	-3.967283
H142	4.916027	-0.885500	-3.859647
H143	1.480478	-1.907826	-2.670533
H144	1.114077	-1.075638	-4.177205
H145	1.046843	-2.844135	-4.112613
H146	2.585726	-0.143010	-6.037867
H147	-0.762518	-4.713340	-3.549868
H148	0.394460	-5.548506	-4.583323
H149	-0.734212	-6.691796	-1.981499
H150	1.137898	-8.336318	-2.145997
H151	0.072316	-8.488416	-3.541019
H152	1.620979	-7.648820	-3.702668
H153	1.472959	-6.123219	-0.973065
H154	0.640461	-4.665917	-1.541177
H155	1.976444	-5.347475	-2.481185
H156	-1.341769	-7.390180	-4.925886
H157	-5.345114	-6.893203	-3.280807
H158	-4.225212	-4.373483	0.660735
H159	-5.544525	-4.774140	2.737869
H160	-3.929158	-4.223048	3.160981
H161	-4.339055	-5.937688	3.298291
H162	-2.369802	-6.111975	0.150320
H163	-2.298743	-6.395458	1.881497
H164	-1.810864	-3.707199	0.459905
H165	-0.567147	-4.743997	1.187634
H166	-1.742375	-3.909491	2.212089
H167	-4.698609	-7.366286	1.109978
H168	-1.481717	-2.606951	6.682214
H169	-0.584743	-3.340666	5.359053
H170	-2.252354	-5.237726	5.275218
H171	8.717675	1.825867	-5.435502
H172	9.982906	0.364693	-3.847160
H173	10.563320	-0.029431	-0.970125
H174	11.136114	-1.697863	-0.683366
H175	-7.283163	-1.430489	0.908378
H176	-7.147337	0.335369	1.127954
H177	-7.033472	-0.376578	-0.487359
H178	-2.778616	-1.162215	2.575464
C179	0.728711	-1.610976	0.736468
C180	0.597840	-0.334652	1.450129
O181	0.399679	-0.818223	-0.415167
C182	1.889541	0.419315	1.411460
C183	2.211951	-1.945520	0.738427
C184	2.907213	-0.559441	0.757789
H185	-0.365727	0.103452	1.673469
H186	1.770566	1.348309	0.844938
H187	3.854056	-0.588330	1.301858
H188	2.157689	0.703878	2.432881
H189	3.120644	-0.229825	-0.260617
H190	2.436759	-2.522358	1.641535
H191	2.487888	-2.549552	-0.130137
H192	0.026452	-2.413657	0.950964
H193	1.265000	3.402549	-5.780177
H194	8.412323	2.311761	-3.758638
H195	8.798394	-0.591844	-4.725974
H196	9.151919	5.999286	-0.610872
H197	9.228493	4.544434	-1.632806
H198	9.619990	2.649646	2.305700
H199	2.825763	1.134893	10.128929
H200	3.024442	-5.788476	7.146669
H201	2.771218	-4.065770	7.472815
H202	-1.610867	-5.076433	6.918400
H203	-3.223473	-4.441151	6.515271
H204	-5.976511	-6.181880	0.853346
H205	-5.176134	-8.664865	-3.271826
H206	-1.833638	-5.802400	-5.505898
H207	3.318204	-0.925488	-7.432909
H208	-0.806395	-1.171629	-8.634152

H209	-3.395347	-2.345554	-4.174860
H210	-2.840948	-0.879235	0.805688
H211	-4.785976	-0.569695	-0.248942
H212	-0.945305	8.674275	-4.451336
H213	0.536248	7.894415	-3.838766
H214	-6.375465	9.715430	-0.958555
H215	-5.476789	8.461083	-1.842729
H216	-5.551183	6.036030	1.906636
H217	7.193136	1.389834	-4.650873
H218	11.359820	-0.948368	-2.277642
H219	8.330411	0.579242	-1.929599
H220	7.237415	-0.514316	-2.792912
H221	-2.286850	7.082011	-0.506004
H222	-1.782630	-0.308111	-7.421667
H223	-6.563666	2.152079	-5.181680
H224	-4.903437	2.711357	-5.446980
H225	-2.499181	-1.300973	-5.292576
H226	2.903817	3.254583	-5.120794
H227	4.651361	-2.134296	5.692033
H228	3.508159	-1.188031	4.798928
H229	8.412616	4.508708	1.215327
H230	1.232854	-2.160755	-7.706918
H231	-2.147551	7.525565	-2.618434
H232	-7.602708	7.747746	0.094571
H233	-4.711386	-1.470727	3.712853
H234	-6.247921	-1.207332	2.948404
H235	-4.562097	-5.760284	-1.378665
H236	-2.935620	-8.425910	-3.812939
H237	-0.652391	-0.867906	-0.647293
H238	-0.333875	-1.240988	3.816803
H239	1.159245	-1.785398	3.687061
C240	7.681709	1.123829	3.415976
H241	8.095259	0.119352	3.251045
H242	8.399822	1.645122	4.063194
H243	7.195352	0.135729	0.797300
S244	2.498501	4.106456	-2.293951
H245	2.052487	5.232657	-1.693717
H246	0.450582	4.304310	-3.582879
C247	6.343835	1.018689	4.153777
H248	5.900745	2.009786	4.304977
H249	5.613933	0.414120	3.607292
H250	6.477038	0.563492	5.140835
H251	5.740428	0.962910	1.385087

TS attack at C2:

C1	-6.146921	5.785349	1.300615
C2	-5.743395	6.750069	0.196716
O3	-4.649718	6.648421	-0.360930
C4	-7.596976	5.780148	1.794391
C5	-7.850522	4.846088	2.987938
S6	-7.561127	3.049262	2.699264
C7	-5.876321	2.820135	3.378191
N8	-6.633868	7.731618	-0.104831
C9	-6.351056	8.755125	-1.084361
C10	-1.137138	6.890686	-0.144150
C11	-0.346100	7.162479	-1.412507
O12	0.880827	7.051532	-1.460514
C13	-0.923176	5.447459	0.347517
C14	-1.741278	5.077403	1.598875
C15	-1.610403	3.575202	1.892923
C16	-1.341293	5.903201	2.830770
N17	-1.087902	7.546471	-2.490368
C18	-0.494347	7.812678	-3.787152
C19	2.848467	0.666828	9.034855
C20	1.778788	-0.041989	8.481191
C21	3.755125	1.296676	8.178052
C22	1.620311	-0.143313	7.096269
C23	3.607443	1.207869	6.794670
C24	2.543459	0.479016	6.246983
O25	2.496153	0.371529	4.881195
C26	2.732615	-5.115834	6.526302
C27	3.816003	-4.538871	5.605690
C28	3.308462	-3.287881	4.910495
N29	4.058682	-2.172027	5.010864
O30	2.241334	-3.323600	4.276428
C31	9.088799	1.892418	1.470537
C32	9.148495	2.680644	0.181631
O33	9.488608	2.188077	-0.894157
C34	7.679946	1.754784	2.094047
C35	6.694081	1.124019	1.101079
N36	8.806695	3.989394	0.318235
C37	8.733078	4.883217	-0.809366
C38	8.227989	1.464236	-4.562030
C39	8.859502	0.145804	-4.098452
C40	8.242830	-0.337968	-2.784246
S41	9.009197	-1.843670	-2.057193
C42	10.648626	-1.175894	-1.585178
C43	1.814365	3.286243	-4.805812
C44	1.531761	4.386664	-3.788489
C45	-5.618913	2.406323	-4.577494
C46	-5.688907	3.341775	-3.361675
C47	-6.662436	2.792179	-2.309497
C48	-4.297718	3.562063	-2.749839
C49	-6.769689	-0.524035	0.626052
N50	-5.324260	-0.605432	0.696009
C51	-4.626361	-0.862112	1.810627
N52	-5.240616	-1.072212	3.001007
N53	-3.297310	-0.860421	1.764303
C54	-3.102250	-1.239441	-4.341259
C55	-2.177116	-0.782426	-3.204981
C56	-2.857443	-0.657498	-1.839700
O57	-4.082111	-0.433182	-1.779174
O58	-2.112290	-0.777548	-0.786612
C59	-1.112042	-0.903140	-7.582514
C60	0.171847	-0.432503	-6.927593
O61	0.274806	0.659358	-6.376972
N62	1.200676	-1.323294	-7.025148
C63	2.564771	-1.014497	-6.636205
C64	3.181410	-2.024879	-5.646995
C65	2.992691	-1.716970	-4.145215
C66	3.815032	-0.492938	-3.713276
C67	1.519239	-1.560393	-3.745502
C68	-1.739238	-6.296681	-4.757508
C69	-2.999545	-6.301132	-3.902172
O70	-3.504587	-5.266349	-3.462982
C71	-0.580660	-5.546740	-4.076415
C72	0.000733	-6.235253	-2.825149

C73	0.690308	-7.568427	-3.152621
C74	0.972973	-5.291700	-2.102156
N75	-3.556788	-7.521752	-3.678132
C76	-4.870652	-7.656860	-3.071868
C77	-4.890683	-7.751894	-1.540213
O78	-4.964099	-8.835406	-0.968362
N79	-4.870387	-6.548974	-0.914610
C80	-4.990221	-6.422545	0.529553
C81	-4.128874	-5.289666	1.106040
C82	-2.628162	-5.548852	0.871474
C83	-4.477223	-5.110542	2.589301
C84	-1.731565	-4.322248	1.071038
C85	-2.183922	-4.778902	6.041447
C86	-1.560610	-3.414404	5.708681
C87	-2.391884	-2.548467	4.744514
O88	-3.638751	-2.602041	4.806839
O89	-1.747086	-1.790788	3.928119
O90	0.705948	-1.222871	3.549365
H91	-7.891069	6.786403	2.129886
H92	-8.270051	5.504384	0.973627
H93	-7.244816	5.145747	3.849970
H94	-8.898445	4.927821	3.290674
H95	-5.827718	3.175511	4.410631
H96	-5.681233	1.745044	3.372081
H97	-5.116644	3.320036	2.774131
H98	-5.877587	4.787101	0.942202
H99	-7.136031	8.800001	-1.846743
H100	-1.199432	4.759811	-0.462204
H101	0.146025	5.289859	0.535210
H102	-2.795243	5.289255	1.368934
H103	-2.211454	3.282863	2.761280
H104	-1.939105	2.971611	1.039939
H105	-0.568004	3.310724	2.111099
H106	-1.929181	5.609513	3.707699
H107	-1.494716	6.976034	2.677287
H108	-0.283348	5.749426	3.076952
H109	-0.772696	7.600698	0.607225
H110	-0.668756	6.992847	-4.494612
H111	1.056855	-0.529724	9.130304
H112	4.585982	1.864629	8.587576
H113	0.792019	-0.705092	6.673095
H114	4.303654	1.698909	6.123020
H115	1.678029	-0.077500	4.553615
H116	4.062519	-5.266141	4.823404
H117	4.738009	-4.334906	6.160720
H118	1.823003	-5.314399	5.956181
H119	7.310676	2.761360	2.347183
H120	6.621870	1.711749	0.181174
H121	9.488663	0.897990	1.252422
H122	7.696815	5.098678	-1.097245
H123	1.755915	5.371034	-4.206933
H124	1.580261	2.296727	-4.405500
H125	-6.069528	4.316237	-3.703745
H126	-6.744499	3.464438	-1.447800
H127	-7.667716	2.652921	-2.723915
H128	-6.310428	1.821051	-1.941732
H129	-4.331949	4.288294	-1.929995
H130	-3.589343	3.935318	-3.498570
H131	-3.902312	2.617604	-2.357138
H132	-5.253242	1.418369	-4.274284
H133	-1.750270	0.202089	-3.440995
H134	-1.323514	-1.458709	-3.098872
H135	-3.981256	-0.594449	-4.406280
H136	-1.363785	-1.929520	-7.297780
H137	2.775256	-3.021844	-5.868647
H138	4.258011	-2.087253	-5.851101
H139	3.397772	-2.585903	-3.605583
H140	3.747874	-0.338960	-2.631455
H141	3.457194	0.427313	-4.187760
H142	4.874017	-0.613244	-3.969269
H143	1.419887	-1.411250	-2.665703
H144	1.064009	-0.695540	-4.237815
H145	0.936836	-2.447396	-4.019735
H146	2.540553	-0.005674	-6.220684
H147	-0.951752	-4.553390	-3.801195
H148	0.223896	-5.395775	-4.808964
H149	-0.832185	-6.443060	-2.137595
H150	1.089746	-8.034590	-2.245959
H151	0.008928	-8.291022	-3.613836
H152	1.528753	-7.414381	-3.843228
H153	1.370369	-5.756268	-1.193144
H154	0.481761	-4.355438	-1.817944
H155	1.825900	-5.039493	-2.744521
H156	-1.446386	-7.314414	-5.037073
H157	-5.474610	-6.805771	-3.400568
H158	-4.399191	-4.362172	0.575505
H159	-5.551037	-4.934529	2.722137
H160	-3.956028	-4.270117	3.049363
H161	-4.223274	-6.014720	3.156524
H162	-2.490990	-5.918271	-0.151918
H163	-2.304210	-6.361622	1.536835
H164	-1.993292	-3.525999	0.364244
H165	-0.680929	-4.586406	0.903383
H166	-1.814006	-3.908607	2.080858
H167	-4.706471	-7.387858	0.959728
H168	-1.452260	-2.830136	6.634063
H169	-0.552546	-3.533482	5.297805
H170	-2.232091	-5.416784	5.153370
H171	8.674653	1.810464	-5.500119
H172	9.936572	0.295070	-3.960864
H173	10.530427	-0.197734	-1.112337
H174	11.089749	-1.878322	-0.874153
H175	-7.257429	-1.455061	0.947244
H176	-7.169961	0.306627	1.220345
H177	-7.041286	-0.353690	-0.416433
H178	-2.756094	-1.156812	2.592239
C179	0.674422	-1.425183	1.365480
C180	0.501032	-0.045696	0.888798
O181	0.312736	-0.694396	-0.384034
C182	1.869968	0.608966	0.957154
C183	2.097211	-1.856952	1.158004
C184	2.856571	-0.566513	0.733622
H185	-0.360081	0.536536	1.217616
H186	1.975761	1.404487	0.214499
H187	3.779470	-0.436926	1.303408
H188	1.993436	1.043234	1.955239
H189	3.130229	-0.631066	-0.320976
H190	2.477038	-2.308839	2.076764
H191	2.135783	-2.638239	0.392802
H192	-0.161931	-2.095258	1.506610

H193	1.194500	3.420297	-5.697143
H194	8.374108	2.244819	-3.808302
H195	8.746362	-0.628670	-4.866679
H196	9.237466	5.829901	-0.589731
H197	9.233368	4.399691	-1.648240
H198	9.761859	2.360004	2.201943
H199	2.964685	0.736430	10.111534
H200	3.071998	-6.050433	6.981295
H201	2.485724	-4.416632	7.331209
H202	-1.596048	-5.301730	6.802704
H203	-3.203685	-4.647149	6.410265
H204	-6.042556	-6.250696	0.798201
H205	-5.325119	-8.578416	-3.440371
H206	-2.005452	-5.777709	-5.686058
H207	3.187659	-0.971645	-7.540405
H208	-1.010890	-0.872988	-8.673745
H209	-3.447955	-2.264935	-4.174320
H210	-2.848171	-0.776410	0.841719
H211	-4.798507	-0.490784	-0.194891
H212	-0.899328	8.735955	-4.213716
H213	0.580028	7.923755	-3.639194
H214	-6.258088	9.743770	-0.619509
H215	-5.405257	8.500132	-1.561610
H216	-5.468535	5.994951	2.138966
H217	7.149615	1.356563	-4.726966
H218	11.312822	-1.087156	-2.448276
H219	8.290750	0.455313	-2.033510
H220	7.189808	-0.604405	-2.928402
H221	-2.206658	7.077451	-0.287653
H222	-1.924335	-0.238037	-7.292399
H223	-6.604747	2.272321	-5.037562
H224	-4.939786	2.796091	-5.343779
H225	-2.576101	-1.205493	-5.296874
H226	2.864673	3.295471	-5.115477
H227	4.848071	-2.146168	5.636635
H228	3.664763	-1.278495	4.718769
H229	8.490827	4.314065	1.218982
H230	1.038443	-2.182682	-7.529226
H231	-2.093160	7.532717	-2.401915
H232	-7.512303	7.762066	0.386750
H233	-4.679056	-1.555083	3.728592
H234	-6.222728	-1.299470	2.979101
H235	-4.724677	-5.723865	-1.486215
H236	-3.086260	-8.352031	-4.001925
H237	-0.726931	-0.759134	-0.632234
H238	-0.263281	-1.452138	3.795412
H239	1.238162	-2.015534	3.808399
C240	7.780633	0.963190	3.413492
H241	8.079046	-0.069061	3.184195
H242	8.594084	1.387435	4.017138
H243	7.015341	0.113266	0.823726
S244	2.568197	4.164648	-2.271731
H245	2.125430	5.278145	-1.645376
H246	0.480012	4.373451	-3.490856
C247	6.498867	0.958406	4.251633
H248	6.181760	1.980634	4.488708
H249	5.667218	0.469720	3.735707
H250	6.654944	0.435213	5.201041
H251	5.687583	1.059304	1.523615

4.3 Mutant *R2*

The following atoms were kept fixed in the geometry optimizations of reactant and transition state structures: 1, 6, 7, 15, 16, 23, 28, 34, 35, 40, 42, 46, 51, 56, 60, 65, 73, 77, 82.

Reactant:

C1	-5.329037	6.750827	1.823141
C2	-4.816433	7.642417	0.691521
O3	-3.807550	7.374060	0.040240
C4	-4.887235	5.299858	1.634126
N5	-5.556024	8.765329	0.478397
C6	-5.194841	9.772029	-0.501441
C7	-0.287934	7.113626	0.131190
C8	0.455416	7.230102	-1.191560
O9	1.638260	6.908445	-1.321961
C10	0.085242	5.838662	0.898383
C11	-0.537295	5.731156	2.304058
C12	-0.334924	4.317665	2.869216
C13	0.022211	6.781635	3.275819
N14	-0.266356	7.771919	-2.213856
C15	0.304926	8.011938	-3.526169
C16	3.151893	0.142200	8.987961
C17	1.982148	-0.412938	8.463286
C18	4.091659	0.693719	8.113601
C19	1.753581	-0.436793	7.087011
C20	3.875302	0.680613	6.736890
C21	2.706625	0.108770	6.213348
O22	2.568351	0.068287	4.859008
C23	2.024903	-5.495024	6.400095
C24	3.138296	-5.056901	5.438621
C25	2.764635	-3.753467	4.747582
N26	3.644036	-2.731833	4.848428
O27	1.698788	-3.666421	4.122917
C28	9.118641	0.579573	1.122253
C29	9.208623	1.374239	-0.161294
O30	9.355288	0.858437	-1.269529
C31	7.728704	0.601253	1.802867
C32	6.642231	0.060017	0.863310
N33	9.121948	2.720218	0.018471
C34	9.111734	3.642178	-1.089028
C35	7.900598	0.428804	-4.861879
C36	8.696981	-0.853247	-4.592903
C37	8.195050	-1.578964	-3.342258
S38	9.141330	-3.084990	-2.876077
C39	10.723437	-2.325224	-2.352361
C40	1.838160	3.211732	-4.735991
C41	1.755357	4.278352	-3.648780
C42	-5.621188	3.469293	-4.126227
C43	-5.499254	4.599823	-3.095428
C44	-6.514622	4.410506	-1.960235
C45	-4.066968	4.691983	-2.550172
C46	-6.944996	0.627474	1.085276
N47	-5.499100	0.500091	1.157306
C48	-4.831929	-0.033800	2.204862
N49	-5.466822	-0.403147	3.324963
N50	-3.505906	-0.147760	2.147295

C51	-3.684685	-0.521805	-4.080538
C52	-2.623346	-0.151372	-3.039361
C53	-3.204398	0.227739	-1.690877
O54	-4.356811	0.606962	-1.529361
O55	-2.393844	0.155294	-0.628489
C56	-1.831706	-0.416839	-7.417484
C57	-0.515075	-0.282085	-6.682250
O58	-0.371008	0.462613	-5.714445
N59	0.471274	-1.074699	-7.181754
C60	1.827364	-1.109098	-6.669924
C61	2.253190	-2.475650	-6.077497
C62	2.276158	-2.565491	-4.535269
C63	3.381259	-1.682952	-3.934470
C64	0.911017	-2.260487	-3.902154
C65	-3.135909	-5.716510	-4.646770
C66	-4.337137	-5.542870	-3.727739
O67	-4.646957	-4.452103	-3.241675
C68	-1.810835	-5.396541	-3.929155
C69	-1.409504	-6.380280	-2.811091
C70	-1.094917	-7.785327	-3.347072
C71	-0.212766	-5.828013	-2.023097
N72	-5.060713	-6.668270	-3.486244
C73	-6.350241	-6.622877	-2.818162
C74	-6.333759	-6.739502	-1.287347
O75	-6.637546	-7.788087	-0.726849
N76	-6.017571	-5.587599	-0.645686
C77	-6.098170	-5.469372	0.803156
C78	-5.058731	-4.508149	1.393086
C79	-3.620141	-4.993212	1.127636
C80	-5.356068	-4.324030	2.886676
C81	-2.547913	-3.913056	1.299371
C82	-2.799890	-4.401835	6.182706
C83	-2.104244	-3.053462	5.939389
C84	-2.854856	-2.118622	4.976093
O85	-4.106111	-2.043339	5.071250
O86	-2.153926	-1.456304	4.135206
O87	0.500931	-1.181903	3.936345
H88	-5.291140	4.897389	0.702971
H89	-3.798860	5.255069	1.580705
H90	-4.927420	7.139837	2.767760
H91	-6.008781	9.937259	-1.215009
H92	-0.226533	4.969810	0.304546
H93	1.177455	5.779904	0.974429
H94	-1.620375	5.905922	2.209197
H95	-0.791798	4.213022	3.858778
H96	-0.778152	3.559259	2.214256
H97	0.731974	4.085757	2.970058
H98	-0.440794	6.685957	4.263816
H99	-0.154101	7.805002	2.929139
H100	1.104237	6.654661	3.402722
H101	-0.002786	7.998582	0.716072

H102	-0.043920	7.280723	-4.265579
H103	1.237004	-0.838551	9.130246
H104	5.002125	1.139790	8.505325
H105	0.851234	-0.878075	6.675551
H106	4.596409	1.110373	6.049361
H107	1.669563	-0.296850	4.586924
H108	3.267221	-5.812003	4.654518
H109	4.096538	-4.966854	5.962128
H110	1.077355	-5.571913	5.862739
H111	7.479150	1.646629	2.043909
H112	6.605571	0.622369	-0.074361
H113	9.389496	-0.451931	0.878476
H114	8.110480	4.048798	-1.277796
H115	2.265977	5.192650	-3.960404
H116	1.319958	2.292238	-4.448507
H117	-5.733034	5.548903	-3.600803
H118	-6.466765	5.235108	-1.239567
H119	-7.540860	4.360172	-2.341537
H120	-6.315582	3.477970	-1.416881
H121	-3.962541	5.498743	-1.817311
H122	-3.349016	4.872574	-3.358448
H123	-3.782094	3.751016	-2.064055
H124	-5.408165	2.502053	-3.655621
H125	-2.021626	0.696328	-3.393134
H126	-1.908853	-0.969458	-2.895953
H127	-4.438234	0.263792	-4.166009
H128	-2.368973	-1.305927	-7.071073
H129	1.586877	-3.252249	-6.476387
H130	3.256909	-2.719226	-6.446996
H131	2.531082	-3.608657	-4.297553
H132	3.465606	-1.847762	-2.855908
H133	3.177675	-0.616281	-4.081387
H134	4.357052	-1.904490	-4.381841
H135	0.954637	-2.358876	-2.812770
H136	0.584577	-1.239734	-4.124221
H137	0.142124	-2.947863	-4.273046
H138	1.890253	-0.312829	-5.926304
H139	-1.901408	-4.389688	-3.506466
H140	-1.005960	-5.356228	-4.675195
H141	-2.255636	-6.464213	-2.113429
H142	-0.808092	-8.458247	-2.532638
H143	-1.949212	-8.242727	-3.857399
H144	-0.261966	-7.753065	-4.060310
H145	0.067168	-6.501112	-1.206141
H146	-0.439642	-4.848721	-1.589097
H147	0.664286	-5.710569	-2.671460
H148	-3.115779	-6.719105	-5.086511
H149	-6.836560	-5.685588	-3.106643
H150	-5.184822	-3.532769	0.893961
H151	-6.380664	-3.966440	3.042698
H152	-4.690100	-3.608505	3.370014
H153	-5.260953	-5.280179	3.415890
H154	-3.562343	-5.385315	0.104790
H155	-3.406703	-5.841895	1.792224
H156	-2.718802	-3.086931	0.597981
H157	-1.551313	-4.320696	1.097850
H158	-2.531815	-3.487547	2.306778
H159	-5.973078	-6.475994	1.213653
H160	-2.018811	-2.514702	6.893504
H161	-1.086241	-3.199268	5.565897
H162	-2.831942	-5.000873	5.266716
H163	8.265820	0.942405	-5.757670
H164	9.754087	-0.595036	-4.462173
H165	10.530375	-1.457524	-1.716932
H166	11.269948	-3.082171	-1.785065
H167	-7.457123	-0.339641	1.180586
H168	-7.325628	1.311574	1.852341
H169	-7.193801	1.043575	0.108679
H170	-2.987675	-0.617580	2.926861
C171	0.257163	-1.381198	0.718660
C172	0.582277	0.042484	0.685456
O173	0.074060	-0.618278	-0.529084
C174	2.082328	0.220432	0.687634
C175	1.534514	-2.189405	0.711879
C176	2.620802	-1.172602	0.273814
H177	-0.078678	0.805818	1.086770
H178	2.415425	1.025643	0.025853
H179	3.595368	-1.391340	0.717467
H180	2.374826	0.474362	1.714026
H181	2.739461	-1.203583	-0.811461
H182	1.702341	-2.556201	1.729506
H183	1.477586	-3.060317	0.050172
H184	-0.657215	-1.764098	1.159795
H185	1.367356	3.576039	-5.655410
H186	7.986085	1.119288	-4.016421
H187	8.643680	-1.528739	-5.455369
H188	9.797713	4.476334	-0.908241
H189	9.438433	3.097931	-1.975031
H190	9.870554	0.956987	1.828088
H191	3.322683	0.154071	10.059963
H192	2.256719	-6.467391	6.844015
H193	1.898472	-4.772233	7.212278
H194	-2.271122	-4.982108	6.945752
H195	-3.829027	-4.240316	6.512307
H196	-7.105911	-5.134914	1.089548
H197	-6.952102	-7.459364	-3.177559
H198	-3.277543	-5.007857	-5.470205
H199	2.507964	-0.835984	-7.485794
H200	-1.694390	-0.509496	-8.499604
H201	-4.189283	-1.455993	-3.814752
H202	-3.039923	0.055569	1.270538
H203	-4.986181	0.626008	0.280770
H204	0.050401	9.016305	-3.879852
H205	1.387458	7.924583	-3.433341
H206	-4.946674	10.726294	-0.023209
H207	-4.319419	9.406484	-1.037480
H208	-6.421393	6.817789	1.893325
H209	6.835786	0.214853	-5.012233
H210	11.330541	-2.022217	-3.208989
H211	8.200556	-0.900504	-2.484732
H212	7.167502	-1.931411	-3.488666
H213	-1.372211	7.171114	-0.014594
H214	-2.447286	0.458525	-7.209345
H215	-6.629453	3.421278	-4.552882
H216	-4.913172	3.605448	-4.951173
H217	-3.207305	-0.652310	-5.051660
H218	2.879810	2.964227	-4.965032
H219	4.414162	-2.795356	5.495322
H220	3.356548	-1.792788	4.574589
H221	8.944214	3.071422	0.946899

H222	0.255180	-1.652624	-7.980365
H223	-1.248644	7.946457	-2.061578
H224	-6.371007	8.918962	1.052674
H225	-4.946619	-0.977151	4.036425
H226	-6.472955	-0.424827	3.333667
H227	-5.734092	-4.794792	-1.211535
H228	-4.744039	-7.549379	-3.859251
H229	-1.453099	-0.175552	-0.785412
H230	-0.477387	-1.244421	4.092164
H231	0.837894	-2.101668	3.947989
C232	7.798340	-0.170907	3.135369
H233	7.981959	-1.232692	2.919758
H234	8.674132	0.179235	3.698354
H235	6.836777	-0.989145	0.611394
S236	2.551942	3.688375	-2.087560
H237	2.389995	4.849450	-1.414441
H238	0.713215	4.528324	-3.433190
S239	-5.488679	4.294803	3.059205
H240	-4.853268	3.153465	2.726828
C241	6.556936	-0.028142	4.020829
H242	6.351528	1.025461	4.242358
H243	5.660192	-0.444125	3.553064
H244	6.699106	-0.541945	4.977233
H245	5.651252	0.121371	1.321401

TS attack at C1:

C1	-5.423478	6.627053	2.034097
C2	-4.917184	7.573162	0.947212
O3	-3.879723	7.362978	0.319865
C4	-5.106946	5.171224	1.690271
N5	-5.689654	8.676590	0.748196
C6	-5.336888	9.724365	-0.190428
C7	-0.386370	7.130662	0.366475
C8	0.357965	7.297845	-0.950600
O9	1.542076	6.986138	-1.091015
C10	0.016041	5.847364	1.105015
C11	-0.655272	5.670119	2.480361
C12	-0.398332	4.255998	3.021512
C13	-0.198539	6.725171	3.499505
N14	-0.368160	7.870335	-1.953084
C15	0.197426	8.157835	-3.258276
C16	3.156664	-0.066554	8.999121
C17	1.934024	-0.522772	8.500830
C18	4.137852	0.363576	8.102979
C19	1.692253	-0.570116	7.126233
C20	3.909046	0.326215	6.729112
C21	2.687222	-0.148412	6.236191
O22	2.544184	-0.218376	4.876216
C23	2.130706	-5.634678	6.226242
C24	2.913834	-5.369827	4.926549
C25	2.637395	-3.968844	4.406224
N26	3.552609	-3.020579	4.701322
O27	1.601506	-3.725440	3.767358
C28	9.128771	0.728850	1.165602
C29	9.200750	1.565758	-0.091154
O30	9.327482	1.084731	-1.217370
C31	7.701426	0.560619	1.740536
C32	6.754936	-0.068384	0.709398
N33	9.102878	2.903937	0.132447
C34	9.073374	3.861436	-0.944656
C35	7.923919	0.752764	-4.823050
C36	8.706881	-0.546016	-4.597531
C37	8.196894	-1.304789	-3.370078
S38	9.137521	-2.824318	-2.936491
C39	10.717514	-2.078571	-2.386466
C40	1.814589	3.426476	-4.620436
C41	1.699250	4.425854	-3.473840
C42	-5.649123	3.536954	-4.019759
C43	-5.448467	4.694012	-3.033313
C44	-6.681625	4.867205	-2.135309
C45	-4.175135	4.487789	-2.200519
C46	-6.933250	0.504420	1.093255
N47	-5.490924	0.350664	1.115656
C48	-4.800313	-0.159806	2.152455
N49	-5.387313	-0.416462	3.338474
N50	-3.491469	-0.358453	2.021308
C51	-3.644769	-0.419958	-4.099772
C52	-2.618930	-0.249444	-2.970438
C53	-3.200704	-0.072069	-1.569307
O54	-4.366034	0.345083	-1.423874
O55	-2.424782	-0.355397	-0.569285
C56	-1.788038	-0.174597	-7.427249
C57	-0.457997	0.001784	-6.723470
O58	-0.272598	0.850526	-5.855737
N59	0.499052	-0.878776	-7.132081
C60	1.881020	-0.828486	-6.694243
C61	2.372730	-2.108682	-5.980647
C62	2.288562	-2.109210	-4.438123
C63	3.285666	-1.118546	-3.816997
C64	0.867094	-1.861618	-3.915090
C65	-3.006297	-5.583317	-4.833813
C66	-4.206859	-5.467270	-3.904365
O67	-4.532749	-4.402669	-3.375451
C68	-1.689192	-5.221382	-4.122807
C69	-1.237040	-6.211199	-3.030729
C70	-0.899473	-7.599430	-3.595583
C71	-0.037567	-5.640022	-2.260530
N72	-4.914152	-6.612400	-3.705533
C73	-6.207850	-6.603482	-3.043128
C74	-6.197152	-6.771469	-1.516774
O75	-6.492684	-7.841501	-0.992790
N76	-5.891315	-5.639440	-0.836409
C77	-5.981847	-5.564666	0.614445
C78	-4.930637	-4.638020	1.235624
C79	-3.503366	-5.160844	0.984848
C80	-5.243861	-4.460158	2.726609
C81	-2.411745	-4.100078	1.145471
C82	-2.711681	-4.617283	6.033515
C83	-1.916550	-3.346694	5.699728
C84	-2.683118	-2.325077	4.841686
O85	-3.921271	-2.224645	4.980232
O86	-1.993290	-1.610921	4.024344
O87	0.476037	-1.250444	3.535202
H88	-5.619105	4.878435	0.771828
H89	-4.033504	5.060175	1.535593
H90	-4.921458	6.900934	2.971204
H91	-6.110653	9.848125	-0.955587

H92	-0.237306	4.986496	0.473335	H152	-4.562391	-3.768464	3.223567
H93	1.106535	5.831636	1.218809	H153	-5.184964	-5.423466	3.248505
H94	-1.740601	5.785864	2.340087	H154	-3.449919	-5.564845	-0.033348
H95	-0.896253	4.100104	3.984380	H155	-3.311576	-6.006216	1.660326
H96	-0.764536	3.492090	2.326894	H156	-2.566471	-3.281181	0.432516
H97	0.674876	4.084295	3.170394	H157	-1.423454	-4.534469	0.955872
H98	-0.688480	6.572225	4.467241	H158	-2.392365	-3.660918	2.147500
H99	-0.428645	7.744381	3.173153	H159	-5.877347	-6.585407	0.994714
H100	0.884608	6.664223	3.662550	H160	-1.645109	-2.830340	6.631994
H101	-0.128136	8.008647	0.973894	H161	-0.976055	-3.590716	5.194347
H102	-0.135429	7.439675	-4.017586	H162	-2.919196	-5.200104	5.130736
H103	1.156921	-0.851300	9.185229	H163	8.293828	1.292012	-5.701602
H104	5.089557	0.733531	8.474376	H164	9.766769	-0.303510	-4.459299
H105	0.743380	-0.933961	6.741558	H165	10.520038	-1.220987	-1.738689
H106	4.660299	0.664763	6.023776	H166	11.257638	-2.845764	-1.826916
H107	1.633764	-0.491830	4.589634	H167	-7.463629	-0.442020	1.271019
H108	2.595391	-6.071122	4.150652	H168	-7.276884	1.244411	1.826596
H109	3.987330	-5.515824	5.091257	H169	-7.212027	0.860692	0.100818
H110	1.057646	-5.513694	6.057171	H170	-2.966581	-0.801171	2.794480
H111	7.315608	1.560879	1.992264	C171	0.286084	-1.623026	0.637716
H112	6.707003	0.526261	-0.206898	C172	0.403360	-0.425290	1.479992
H113	9.538014	-0.255567	0.919131	O173	0.008330	-0.664208	-0.397322
H114	8.066434	4.263275	-1.111344	C174	1.791575	0.130600	1.402363
H115	2.193146	5.367401	-3.724701	C175	1.694426	-2.172753	0.483022
H116	1.281065	2.495650	-4.412283	C176	2.600207	-0.918075	0.585228
H117	-5.328666	5.620611	-3.614804	H177	-0.462890	0.124680	1.821627
H118	-6.561936	5.715739	-1.451863	H178	1.776644	1.115787	0.925363
H119	-7.587718	5.040367	-2.727212	H179	3.558480	-1.149880	1.055988
H120	-6.851062	3.967703	-1.529930	H180	2.170149	0.273627	2.417897
H121	-4.019504	5.309858	-1.493588	H181	2.807734	-0.519402	-0.409500
H122	-3.290483	4.423531	-2.843973	H182	1.884101	-2.876375	1.300415
H123	-4.232195	3.549440	-1.634936	H183	1.813903	-2.708332	-0.462710
H124	-5.739003	2.584894	-3.482685	H184	-0.520008	-2.330216	0.821090
H125	-1.989395	0.627891	-3.171824	H185	1.374941	3.850717	-5.529526
H126	-1.930676	-1.100698	-2.941922	H186	8.018313	1.412821	-3.954587
H127	-4.349459	0.414199	-4.119268	H187	8.646077	-1.192892	-5.481084
H128	-2.275780	-1.099811	-7.103227	H188	9.752723	4.695867	-0.742232
H129	1.809967	-2.965215	-6.376521	H189	9.397092	3.349715	-1.850946
H130	3.419297	-2.278481	-6.264113	H190	9.776361	1.169437	1.935007
H131	2.594293	-3.117438	-4.120966	H191	3.336244	-0.036622	10.069044
H132	3.278671	-1.194957	-2.724864	H192	2.310264	-6.654308	6.577977
H133	3.038043	-0.080822	-4.065850	H193	2.431365	-4.943332	7.019704
H134	4.309195	-1.312208	-4.158362	H194	-2.156553	-5.255963	6.728447
H135	0.839986	-1.917452	-2.822119	H195	-3.670783	-4.353715	6.484984
H136	0.505474	-0.868118	-4.196138	H196	-6.986030	-5.220817	0.902633
H137	0.162508	-2.602076	-4.310194	H197	-6.798075	-7.434673	-3.432871
H138	1.964139	0.048043	-6.049186	H198	-3.181502	-4.869173	-5.646158
H139	-1.818157	-4.228349	-3.678317	H199	2.514350	-0.640562	-7.571190
H140	-0.894416	-5.132304	-4.875660	H200	-1.664760	-0.223746	-8.514624
H141	-2.065307	-6.327885	-2.316529	H201	-4.210408	-1.349321	-3.981977
H142	-0.578539	-8.277706	-2.798263	H202	-3.086177	-0.305995	1.075188
H143	-1.753929	-8.069303	-4.094095	H203	-5.002485	0.397121	0.197017
H144	-0.083396	-7.536140	-4.326139	H204	-0.079177	9.165709	-3.584167
H145	0.274868	-6.315635	-1.457190	H205	1.281776	8.091669	-3.168193
H146	-0.278796	-4.670884	-1.812049	H206	-5.188832	10.682611	0.319606
H147	0.822967	-5.495100	-2.925491	H207	-4.404864	9.429882	-0.672078
H148	-2.951388	-6.578370	-5.288071	H208	-6.499159	6.761229	2.197344
H149	-6.703928	-5.662536	-3.301763	H209	6.856757	0.555743	-4.979093
H150	-5.021869	-3.654946	0.745877	H210	11.332585	-1.762517	-3.232573
H151	-6.258617	-4.071991	2.872512	H211	8.199662	-0.649538	-2.494784

H212	7.169357	-1.650652	-3.531249
H213	-1.471150	7.161724	0.218135
H214	-2.435318	0.665657	-7.178573
H215	-6.553836	3.673256	-4.623356
H216	-4.798761	3.448605	-4.704742
H217	-3.105587	-0.453951	-5.048010
H218	2.862652	3.189634	-4.831914
H219	4.327143	-3.233275	5.309949
H220	3.325513	-2.037211	4.558679
H221	8.954583	3.225716	1.076549
H222	0.264614	-1.520704	-7.874891
H223	-1.353018	8.025088	-1.796034
H224	-6.531328	8.778305	1.294379
H225	-4.872206	-1.017312	4.009981
H226	-6.392346	-0.405598	3.394002
H227	-5.613498	-4.825829	-1.375654
H228	-4.593764	-7.472013	-4.122795
H229	-1.051609	-0.545299	-0.544533
H230	-0.497299	-1.426514	3.810321
H231	0.901264	-2.144091	3.520842
C232	7.776687	-0.250776	3.049419
H233	8.119759	-1.268209	2.815956
H234	8.551258	0.190393	3.691119
H235	7.094297	-1.073331	0.432430
S236	2.494130	3.770883	-1.935957
H237	2.339308	4.907815	-1.222310
H238	0.649901	4.639859	-3.255054
S239	-5.652533	4.077934	3.073877
H240	-5.138317	2.935758	2.575238
C241	6.465994	-0.315653	3.838502
H242	6.086708	0.689814	4.053920
H243	5.680970	-0.852678	3.297719
H244	6.614578	-0.825945	4.795964
H245	5.737464	-0.148765	1.101385

TS attack at C2:

C1	-5.356227	6.599913	2.151046
C2	-4.866775	7.551115	1.061265
O3	-3.847638	7.334473	0.406373
C4	-5.111736	5.143150	1.754924
N5	-5.630586	8.664764	0.889748
C6	-5.289974	9.711667	-0.053937
C7	-0.345452	7.086854	0.401170
C8	0.378974	7.261800	-0.925900
O9	1.561605	6.953572	-1.085702
C10	0.078970	5.808830	1.136354
C11	-0.580536	5.624142	2.516395
C12	-0.294203	4.217308	3.061600
C13	-0.137424	6.691551	3.528753
N14	-0.363435	7.837124	-1.914756
C15	0.182390	8.135257	-3.226083
C16	3.303166	-0.188091	8.923983
C17	2.099368	-0.691367	8.423185
C18	4.231062	0.353924	8.030654
C19	1.825807	-0.676332	7.052732
C20	3.970067	0.379676	6.661611
C21	2.768639	-0.143414	6.165217
O22	2.606197	-0.150415	4.804441
C23	2.198691	-5.731568	6.131950
C24	3.300245	-5.223355	5.193097
C25	2.914673	-3.882161	4.593454
N26	3.813590	-2.880438	4.680360
O27	1.812720	-3.746900	4.037508
C28	9.145533	0.627135	0.995245
C29	9.193905	1.470511	-0.258909
O30	9.288267	0.995207	-1.390510
C31	7.754397	0.562054	1.670085
C32	6.697820	-0.001993	0.710638
N33	9.120521	2.808336	-0.024042
C34	9.071965	3.774177	-1.092836
C35	7.839119	0.698216	-4.971701
C36	8.585989	-0.623264	-4.756246
C37	8.070685	-1.366679	-3.521509
S38	8.971417	-2.913847	-3.101147
C39	10.581501	-2.216453	-2.575619
C40	1.749347	3.404324	-4.647126
C41	1.655648	4.399604	-3.495044
C42	-5.702320	3.552113	-3.918544
C43	-5.485085	4.702927	-2.928539
C44	-6.708933	4.877055	-2.018098
C45	-4.203916	4.484232	-2.111145
C46	-6.916406	0.492245	1.195297
N47	-5.471771	0.355214	1.192361
C48	-4.763962	-0.167296	2.212424
N49	-5.337846	-0.458107	3.396112
N50	-3.453134	-0.342299	2.067313
C51	-3.722038	-0.415219	-4.059095
C52	-2.677181	-0.170536	-2.961291
C53	-3.244796	-0.002502	-1.549259
O54	-4.411365	0.407957	-1.388764
O55	-2.458311	-0.283264	-0.558285
C56	-1.920832	-0.157664	-7.416040
C57	-0.571966	0.022781	-6.749066
O58	-0.351087	0.903010	-5.922219
N59	0.360472	-0.891253	-7.142182
C60	1.756409	-0.836824	-6.750078
C61	2.259843	-2.089518	-5.998361
C62	2.209471	-2.032324	-4.455206
C63	3.216082	-1.015251	-3.895093
C64	0.799360	-1.771624	-3.907827
C65	-3.125371	-5.576956	-4.838316
C66	-4.315272	-5.459891	-3.895488
O67	-4.633946	-4.395894	-3.361255
C68	-1.804152	-5.186859	-4.149932
C69	-1.328089	-6.150790	-3.045140
C70	-0.973528	-7.543434	-3.588356
C71	-0.131798	-5.547497	-2.294522
N72	-5.026727	-6.602523	-3.696808
C73	-6.301717	-6.591442	-3.000140
C74	-6.237159	-6.776784	-1.477639
O75	-6.440498	-7.870133	-0.958304
N76	-5.994761	-5.631208	-0.794065
C77	-6.007642	-5.578646	0.659891
C78	-4.960028	-4.615299	1.235139
C79	-3.529091	-5.060264	0.876566
C80	-5.184290	-4.489646	2.747265
C81	-2.458729	-3.983269	1.081957

C82	-2.640440	-4.686064	6.028606	H142	-0.636109	-8.202581	-2.781843
C83	-1.862593	-3.393887	5.734921	H143	-1.824542	-8.034884	-4.071654
C84	-2.625206	-2.379177	4.864606	H144	-0.164081	-7.480723	-4.326320
O85	-3.861091	-2.268531	5.013481	H145	0.196601	-6.204107	-1.481692
O86	-1.937862	-1.679301	4.031637	H146	-0.384131	-4.573535	-1.862622
O87	0.540171	-1.414197	3.515027	H147	0.721537	-5.402248	-2.968606
H88	-5.694912	4.892525	0.866803	H148	-3.064313	-6.578670	-5.276867
H89	-4.055187	5.003890	1.525208	H149	-6.798867	-5.645893	-3.237273
H90	-4.796131	6.831484	3.066323	H150	-5.129874	-3.626385	0.779093
H91	-6.081107	9.846463	-0.799260	H151	-6.209602	-4.170149	2.967064
H92	-0.168622	4.944616	0.506909	H152	-4.517163	-3.766296	3.217247
H93	1.170389	5.806157	1.241771	H153	-5.031925	-5.457656	3.240698
H94	-1.668719	5.718949	2.382131	H154	-3.511507	-5.373953	-0.174026
H95	-0.782884	4.056478	4.028331	H155	-3.280176	-5.953292	1.467199
H96	-0.651902	3.443839	2.373138	H156	-2.653991	-3.109876	0.448304
H97	0.782702	4.064416	3.203443	H157	-1.469262	-4.374148	0.817483
H98	-0.616393	6.531258	4.500780	H158	-2.413243	-3.636212	2.118863
H99	-0.391187	7.704850	3.201452	H159	-5.839015	-6.598778	1.018316
H100	0.948005	6.653416	3.683448	H160	-1.638263	-2.883250	6.682568
H101	-0.089552	7.968357	1.004457	H161	-0.899100	-3.614533	5.263795
H102	-0.154828	7.418127	-3.984422	H162	-2.805897	-5.262779	5.113130
H103	1.360506	-1.107159	9.102583	H163	8.213360	1.226201	-5.855246
H104	5.167421	0.762774	8.400403	H164	9.654444	-0.412226	-4.632976
H105	0.892265	-1.079308	6.669573	H165	10.419806	-1.349411	-1.930531
H106	4.682875	0.802194	5.961368	H166	11.103971	-2.997293	-2.018184
H107	1.713785	-0.464815	4.517958	H167	-7.433300	-0.464458	1.357068
H108	3.427985	-5.924043	4.359458	H168	-7.256336	1.209680	1.952177
H109	4.262984	-5.159526	5.711651	H169	-7.213981	0.869970	0.216372
H110	1.247539	-5.787090	5.599024	H170	-2.918409	-0.801586	2.822404
H111	7.456106	1.587351	1.939724	C171	0.333994	-1.486342	1.343584
H112	6.643429	0.580218	-0.213493	C172	0.342220	-0.074466	0.931803
H113	9.462367	-0.381980	0.715834	O173	-0.029970	-0.619841	-0.348347
H114	8.064587	4.185596	-1.231117	C174	1.795734	0.365211	0.919692
H115	2.102972	5.357852	-3.769114	C175	1.655506	-2.115447	1.008262
H116	1.258616	2.456097	-4.414369	C176	2.572892	-0.933228	0.580320
H117	-5.366542	5.632169	-3.506079	H177	-0.395648	0.612595	1.347233
H118	-6.583636	5.726981	-1.337431	H178	1.965570	1.166680	0.195482
H119	-7.620342	5.048550	-2.602297	H179	3.540621	-0.972397	1.085644
H120	-6.871973	3.978055	-1.410275	H180	2.055717	0.734679	1.917602
H121	-4.032218	5.302075	-1.403068	H181	2.761566	-0.984354	-0.493376
H122	-3.328072	4.416232	-2.766352	H182	2.026344	-2.668598	1.873626
H123	-4.261189	3.543312	-1.549941	H183	1.518387	-2.850842	0.209558
H124	-5.789462	2.597780	-3.385268	H184	-0.584297	-2.029608	1.515700
H125	-2.104537	0.739085	-3.187896	H185	1.250240	3.810922	-5.533021
H126	-1.942638	-0.981612	-2.936138	H186	7.964489	1.355035	-4.104746
H127	-4.488476	0.363024	-4.041240	H187	8.493799	-1.268142	-5.638541
H128	-2.395084	-1.087306	-7.084951	H188	9.763221	4.601334	-0.901267
H129	1.688431	-2.959469	-6.350307	H189	9.368599	3.267241	-2.011005
H130	3.300067	-2.271664	-6.297393	H190	9.877941	1.012217	1.717206
H131	2.527074	-3.026816	-4.107883	H191	3.507091	-0.207331	9.989635
H132	3.234554	-1.049353	-2.801021	H192	2.446098	-6.726519	6.512075
H133	2.958490	0.011626	-4.176622	H193	2.071442	-5.062851	6.988834
H134	4.232461	-1.218066	-4.252143	H194	-2.093307	-5.320089	6.733634
H135	0.795766	-1.797048	-2.813215	H195	-3.618607	-4.447358	6.452090
H136	0.430044	-0.787299	-4.210881	H196	-7.006486	-5.279599	1.009156
H137	0.087876	-2.524238	-4.266209	H197	-6.907397	-7.416038	-3.380733
H138	1.867047	0.064801	-6.145097	H198	-3.319346	-4.879045	-5.660689
H139	-1.940201	-4.187635	-3.721804	H199	2.363940	-0.692159	-7.653192
H140	-1.019215	-5.102000	-4.913592	H200	-1.823720	-0.201732	-8.506438
H141	-2.148844	-6.267674	-2.322334	H201	-4.216416	-1.382280	-3.919891

H202	-3.058830	-0.259113	1.119432
H203	-5.000398	0.417130	0.265775
H204	-0.106895	9.142362	-3.543128
H205	1.268403	8.077745	-3.150996
H206	-5.119066	10.666570	0.455235
H207	-4.373454	9.408772	-0.559459
H208	-6.416313	6.768985	2.372632
H209	6.764599	0.533143	-5.113654
H210	11.195676	-1.924853	-3.431073
H211	8.104827	-0.711981	-2.646391
H212	7.031206	-1.681789	-3.668496
H213	-1.432459	7.106306	0.266741
H214	-2.564726	0.679303	-7.148581
H215	-6.613287	3.695646	-4.511031
H216	-4.860383	3.463612	-4.613850
H217	-3.241570	-0.411347	-5.038806
H218	2.793368	3.203656	-4.909827
H219	4.633760	-2.984803	5.256533
H220	3.521251	-1.929926	4.457102
H221	8.997095	3.124879	0.925386
H222	0.097935	-1.558117	-7.853007
H223	-1.346016	7.989777	-1.741985
H224	-6.457748	8.772458	1.456469
H225	-4.807194	-1.058429	4.056284
H226	-6.342335	-0.464466	3.459911
H227	-5.758488	-4.804386	-1.332071
H228	-4.709329	-7.465410	-4.109454
H229	-1.079515	-0.503329	-0.518132
H230	-0.434186	-1.523129	3.817278
H231	0.970488	-2.285230	3.700543
C232	7.861112	-0.245430	2.979090
H233	8.125436	-1.283571	2.734401
H234	8.699743	0.150342	3.567553
H235	6.936018	-1.036036	0.435111
S236	2.549596	3.775409	-1.999271
H237	2.380848	4.902498	-1.273025
H238	0.611517	4.576293	-3.224051
S239	-5.598373	4.029606	3.143888
H240	-5.145371	2.889006	2.585333
C241	6.599048	-0.224111	3.846014
H242	6.297289	0.803979	4.076686
H243	5.752343	-0.711569	3.354083
H244	6.771671	-0.738043	4.797656
H245	5.702514	0.009502	1.163067

4.4 Mutant *R3*

The following atoms were kept fixed in the geometry optimizations of reactant and transition state structures: 1, 6, 7, 15, 16, 23, 28, 34, 35, 37, 39, 43, 48, 53, 57, 62, 70, 74, 79.

Reactant:

C1	-5.439725	6.791634	1.225611
C2	-4.887551	7.631759	0.070964
O3	-3.797344	7.423285	-0.456159
C4	-4.555451	5.595343	1.559888
N5	-5.712540	8.645274	-0.320004
C6	-5.368688	9.578741	-1.377532
C7	-0.408660	7.091332	-0.508059
C8	0.318266	7.049621	-1.846525
O9	1.468077	6.626116	-1.974075
C10	0.216625	6.146585	0.524193
C11	-0.326668	6.306111	1.956875
C12	0.138272	5.136443	2.836610
C13	0.073611	7.646121	2.593624
N14	-0.384252	7.569323	-2.893551
C15	0.162298	7.647377	-4.236268
C16	3.179693	1.068841	8.964475
C17	1.983254	0.530241	8.485081
C18	4.151557	1.483764	8.051014
C19	1.758912	0.388278	7.115586
C20	3.940447	1.351476	6.679902
C21	2.744257	0.795867	6.202884
O22	2.608956	0.635260	4.857726
C23	2.163701	-4.811375	6.930690
C24	3.296427	-4.361105	6.000877
C25	2.879366	-3.138577	5.195335
N26	3.757590	-2.112033	5.134702
O27	1.787369	-3.124161	4.611856
C28	9.128447	0.870754	1.075507
C29	8.963186	1.536578	-0.272178
O30	8.588259	0.940951	-1.280940
C31	7.869284	0.976486	1.974303
C32	6.665501	0.266261	1.338750
N33	9.254579	2.867982	-0.262922
C34	9.057693	3.707088	-1.418397
C35	7.907609	0.124123	-4.863204
C36	8.730371	-1.116984	-4.476346
C37	1.790549	2.784979	-4.986150
C38	1.489240	3.852574	-3.940992
C39	-5.671846	2.950990	-4.381917
C40	-4.974844	4.022014	-3.533210
C41	-5.704414	4.208070	-2.194720
C42	-3.486936	3.668275	-3.343631
C43	-6.932931	0.594803	1.081253
N44	-5.495301	0.405517	1.145667
C45	-4.817011	0.046104	2.253733
N46	-5.445343	-0.182173	3.417405
N47	-3.488873	-0.048640	2.211253
C48	-3.655408	-0.978061	-3.960575
C49	-2.581872	-0.570819	-2.947347
C50	-3.155734	-0.068007	-1.637152
O51	-4.300380	0.348538	-1.516179
O52	-2.350637	-0.065504	-0.569018
C53	-1.808303	-1.155816	-7.297696
C54	-0.489346	-0.908556	-6.591586
O55	-0.316946	0.036709	-5.825096
N56	0.472384	-1.823625	-6.893383
C57	1.864707	-1.700357	-6.498120
C58	2.401205	-2.869438	-5.643061
C59	2.369358	-2.658581	-4.113897
C60	3.337706	-1.546866	-3.680109
C61	0.952050	-2.407939	-3.579955
C62	-3.002826	-6.191137	-4.029215
C63	-4.207857	-5.953887	-3.129749
O64	-4.542617	-4.827887	-2.753698
C65	-1.683980	-5.782675	-3.344180
C66	-1.268903	-6.652540	-2.140568
C67	-0.940073	-8.098005	-2.544282
C68	-0.077248	-6.014970	-1.411458
N69	-4.905974	-7.066051	-2.775464
C70	-6.196417	-6.982333	-2.112913
C71	-6.178922	-6.951730	-0.578035
O72	-6.473334	-7.944439	0.080780
N73	-5.870972	-5.741686	-0.049232
C74	-5.963923	-5.482986	1.380792
C75	-4.948490	-4.445921	1.874989
C76	-3.500620	-4.932810	1.673549
C77	-5.264555	-4.109307	3.337729
C78	-2.446186	-3.825681	1.754527
C79	-2.682326	-3.840344	6.623948
C80	-2.019794	-2.499043	6.274074
C81	-2.794023	-1.664459	5.240054
O82	-4.047714	-1.628265	5.320831
O83	-2.109509	-1.038928	4.358035
O84	0.529288	-0.710868	4.124376
H85	-4.429493	4.957065	0.684377
H86	-3.571452	5.933979	1.885230
H87	-5.550831	7.436303	2.107034
H88	-6.018763	9.455347	-2.251074
H89	0.051164	5.113322	0.193638
H90	1.302996	6.293307	0.526327
H91	-1.425337	6.274678	1.905443
H92	-0.254423	5.220078	3.855630
H93	-0.188277	4.173893	2.428481
H94	1.232417	5.110932	2.905123
H95	-0.330023	7.733916	3.608223
H96	-0.291133	8.505225	2.021495
H97	1.165000	7.730792	2.662731
H98	-0.346913	8.131043	-0.156611
H99	-0.253337	6.874234	-4.894807
H100	1.214144	0.210117	9.183129
H101	5.083216	1.915331	8.407498
H102	0.837415	-0.044299	6.739438
H103	4.687079	1.673943	5.961209

H104	1.707006	0.250900	4.624521	H164	-2.960226	-0.388051	3.048168
H105	3.524691	-5.157331	5.281585	C165	0.234579	-1.414404	1.012373
H106	4.216091	-4.166116	6.563721	C166	0.724674	-0.090657	0.633813
H107	1.252432	-4.978759	6.353253	O167	0.082072	-0.946305	-0.377267
H108	7.621615	2.044560	2.075739	C168	2.233550	-0.110716	0.567323
H109	6.491042	0.611929	0.317055	C169	1.401735	-2.361224	1.171873
H110	9.358746	-0.183743	0.893235	C170	2.589813	-1.618100	0.506616
H111	8.213061	4.394265	-1.286577	H171	0.180103	0.824143	0.850044
H112	2.158478	4.708630	-4.044098	H172	2.630535	0.461800	-0.276199
H113	1.106172	1.934226	-4.924339	H173	3.540911	-1.836222	0.999542
H114	-5.024786	4.977518	-4.078722	H174	2.601381	0.349201	1.492955
H115	-5.284506	5.040661	-1.623003	H175	2.684413	-1.930116	-0.535558
H116	-6.769415	4.413633	-2.353243	H176	1.563389	-2.519091	2.242174
H117	-5.625789	3.300580	-1.583816	H177	1.208838	-3.339285	0.718261
H118	-3.032989	3.542447	-4.335596	H178	-0.695267	-1.565719	1.550075
H119	-3.425698	2.689346	-2.850822	H179	1.687327	3.212752	-5.990241
H120	-5.670324	1.987812	-3.857551	H180	7.469146	0.583313	-3.972619
H121	-1.949496	0.226731	-3.360080	H181	9.231498	-1.517043	-5.366411
H122	-1.900779	-1.402469	-2.735174	H182	9.955696	4.295302	-1.636079
H123	-4.354474	-0.158653	-4.139777	H183	8.845072	3.053988	-2.265008
H124	-2.208960	-2.146849	-7.058842	H184	9.986031	1.306987	1.603342
H125	1.842444	-3.779329	-5.902825	H185	3.346869	1.173236	10.032082
H126	3.441483	-3.058316	-5.936054	H186	2.430308	-5.738387	7.446242
H127	2.738366	-3.595889	-3.672499	H187	1.950439	-4.050597	7.687997
H128	3.405590	-1.494167	-2.589545	H188	-2.135877	-4.349254	7.424599
H129	3.008308	-0.560976	-4.026588	H189	-3.713307	-3.678554	6.947383
H130	4.348140	-1.725548	-4.061842	H190	-6.980746	-5.144270	1.627993
H131	0.952786	-2.301258	-2.491031	H191	-6.782005	-7.860737	-2.389821
H132	0.532726	-1.484925	-3.992755	H192	-3.155156	-5.564384	-4.914705
H133	0.277852	-3.232472	-3.841130	H193	2.470494	-1.611897	-7.409540
H134	1.948570	-0.749559	-5.969564	H194	-1.682790	-1.101487	-8.384952
H135	-1.791624	-4.742600	-3.016600	H195	-4.221415	-1.844819	-3.605862
H136	-0.878808	-5.797148	-4.090840	H196	-3.017413	0.072924	1.322294
H137	-2.113122	-6.680979	-1.436239	H197	-4.973852	0.491470	0.270428
H138	-0.645452	-8.689304	-1.671309	H198	-0.039289	8.628396	-4.677671
H139	-1.790559	-8.608860	-3.008381	H199	1.239320	7.499189	-4.162347
H140	-0.108478	-8.123667	-3.259276	H200	-5.442888	10.613053	-1.026046
H141	0.211498	-6.605018	-0.535319	H201	-4.339553	9.374571	-1.672050
H142	-0.314789	-5.001533	-1.071981	H202	-6.449755	6.454108	0.959229
H143	0.797058	-5.948724	-2.070489	H203	7.095911	-0.137655	-5.550367
H144	-2.964734	-7.229806	-4.373965	H204	-1.476839	6.883985	-0.633588
H145	-6.700616	-6.086198	-2.488367	H205	-2.525897	-0.396569	-6.989690
H146	-5.085280	-3.531836	1.273386	H206	-6.714373	3.216658	-4.588669
H147	-6.298166	-3.759891	3.445861	H207	-5.165230	2.810143	-5.343613
H148	-4.618645	-3.332409	3.748512	H208	-3.183235	-1.245312	-4.906781
H149	-5.155020	-5.001001	3.967363	H209	2.815682	2.414886	-4.883093
H150	-3.425108	-5.419625	0.693399	H210	4.540542	-2.088270	5.769201
H151	-3.284778	-5.711505	2.418380	H211	3.441222	-1.212796	4.771118
H152	-2.623077	-3.067095	0.982452	H212	9.515978	3.295760	0.611909
H153	-1.441715	-4.233097	1.597555	H213	0.236770	-2.551229	-7.552305
H154	-2.447059	-3.314889	2.721474	H214	-1.350046	7.817285	-2.737211
H155	-5.820580	-6.441068	1.889702	H215	-6.593592	8.760025	0.158122
H156	-1.947658	-1.884109	7.182151	H216	-4.914909	-0.668971	4.180894
H157	-0.998301	-2.649558	5.912253	H217	-6.448903	-0.260850	3.417142
H158	-2.705394	-4.507087	5.755747	H218	-5.608068	-4.999442	-0.688863
H159	8.539803	0.872805	-5.353441	H219	-4.572793	-7.972332	-3.064414
H160	9.517057	-0.808970	-3.778228	H220	-1.419083	-0.434933	-0.688797
H161	-7.486458	-0.328733	1.300019	H221	-0.446717	-0.774344	4.297839
H162	-7.266399	1.382275	1.767233	H222	0.886027	-1.611416	4.267127
H163	-7.183440	0.900520	0.065177	C223	8.198191	0.437991	3.381358

H224	8.433912	-0.632754	3.302937
H225	9.115829	0.924691	3.738578
H226	6.838389	-0.816198	1.293810
S227	1.710400	3.190147	-2.226840
H228	1.749054	4.401958	-1.633732
H229	0.461778	4.212044	-4.045416
S230	-5.351100	4.630190	2.920232
H231	-4.316468	3.797845	3.149464
C232	7.891517	-2.205678	-3.839416
C233	7.540892	-2.130995	-2.482360
H234	7.890160	-1.287781	-1.891087
C235	7.427572	-3.293448	-4.590703
H236	7.697804	-3.368066	-5.641870
C237	6.748913	-3.120665	-1.899229
H238	6.487844	-3.048953	-0.846704
C239	6.290727	-4.199961	-2.658561
H240	5.675428	-4.969913	-2.201424
C241	6.631956	-4.283013	-4.008950
H242	6.284641	-5.120116	-4.608868
C243	-2.676385	4.691931	-2.545053
H244	-2.962376	4.719912	-1.490059
H245	-1.606057	4.465854	-2.588671
H246	-2.823985	5.705001	-2.936019
H247	5.751336	0.437818	1.913858
C248	7.094017	0.646536	4.422364
H249	6.182133	0.099402	4.166367
H250	7.420541	0.302931	5.409389
H251	6.828319	1.706069	4.513008

TS attack at C1:

C1	-5.115071	6.986177	1.592071
C2	-4.547851	7.827403	0.446376
O3	-3.529744	7.523240	-0.172228
C4	-4.464978	5.608735	1.686196
N5	-5.264073	8.957490	0.183103
C6	-4.895588	9.908971	-0.848928
C7	-0.065595	7.140317	-0.106776
C8	0.689115	7.188040	-1.430440
O9	1.864573	6.837267	-1.545883
C10	0.530654	6.117186	0.865892
C11	-0.073251	6.144827	2.283003
C12	0.414146	4.934572	3.092944
C13	0.238244	7.450102	3.030918
N14	-0.021273	7.705868	-2.473829
C15	0.552672	7.877543	-3.795809
C16	3.175631	0.427315	9.020407
C17	1.937820	-0.019097	8.551682
C18	4.162746	0.784703	8.098792
C19	1.686141	-0.127850	7.182533
C20	3.924796	0.684733	6.729462
C21	2.686820	0.220823	6.267331
O22	2.531689	0.087473	4.914212
C23	1.890481	-5.273045	6.654040
C24	2.872500	-5.009470	5.500336
C25	2.588208	-3.667857	4.844852
N26	3.537360	-2.714083	4.960218
O27	1.519890	-3.480848	4.242219
C28	9.153042	0.387906	1.150742
C29	9.028957	1.137098	-0.156622
O30	8.617706	0.619381	-1.194136
C31	7.870678	0.448308	2.020424
C32	6.675901	-0.200373	1.306540
N33	9.379442	2.450288	-0.071053
C34	9.232635	3.360465	-1.178831
C35	7.931773	0.037056	-4.824339
C36	8.641149	-1.291854	-4.511849
C37	1.950148	2.992349	-4.814813
C38	1.895823	4.093538	-3.761084
C39	-5.499032	3.483507	-4.224515
C40	-4.584145	4.377010	-3.379005
C41	-5.277491	4.741650	-2.058558
C42	-3.221619	3.691063	-3.160047
C43	-6.902861	0.886530	1.092735
N44	-5.489223	0.568341	1.104550
C45	-4.808177	0.161713	2.187223
N46	-5.392730	0.063568	3.400537
N47	-3.507882	-0.100845	2.074361
C48	-3.675763	-0.555665	-4.020190
C49	-2.629338	-0.425588	-2.904682
C50	-3.187551	-0.123303	-1.516659
O51	-4.320749	0.383509	-1.402228
O52	-2.433015	-0.397675	-0.498904
C53	-1.820266	-0.633783	-7.356476
C54	-0.477236	-0.455640	-6.675872
O55	-0.221141	0.508241	-5.960060
N56	0.412140	-1.455207	-6.940380
C57	1.817778	-1.398942	-6.577991
C58	2.299022	-2.548106	-5.666082
C59	2.234397	-2.288533	-4.144699
C60	3.244064	-1.214612	-3.710866
C61	0.820654	-1.948409	-3.654810
C62	-3.273533	-5.781962	-4.381822
C63	-4.464708	-5.542996	-3.463378
O64	-4.747594	-4.427379	-3.022837
C65	-1.949167	-5.329760	-3.740668
C66	-1.513502	-6.124646	-2.493595
C67	-1.237848	-7.604707	-2.798602
C68	-0.280560	-5.470005	-1.854115
N69	-5.220543	-6.638585	-3.179810
C70	-6.512290	-6.525109	-2.522951
C71	-6.507984	-6.585973	-0.988375
O72	-6.884773	-7.590882	-0.392602
N73	-6.117296	-5.434307	-0.387503
C74	-6.228146	-5.238111	1.050696
C75	-5.084519	-4.402097	1.636231
C76	-3.732108	-5.129162	1.507394
C77	-5.423474	-4.037324	3.087065
C78	-2.521086	-4.194438	1.562912
C79	-2.901574	-4.053819	6.388229
C80	-2.057081	-2.835430	5.987929
C81	-2.773463	-1.852985	5.045338
O82	-4.006847	-1.691632	5.165166
O83	-2.050326	-1.232526	4.181175
O84	0.433665	-1.028107	3.704656
H85	-4.635487	5.042455	0.770478
H86	-3.389542	5.712620	1.833620
H87	-4.962297	7.537249	2.529569

H88	-5.652471	9.955154	-1.639851
H89	0.397534	5.114576	0.440387
H90	1.613426	6.278976	0.925689
H91	-1.166743	6.065347	2.187000
H92	-0.028797	4.919394	4.094546
H93	0.156041	3.992778	2.596924
H94	1.504219	4.958485	3.212072
H95	-0.195075	7.438044	4.036961
H96	-0.158431	8.329763	2.514214
H97	1.321540	7.586423	3.137135
H98	-0.002510	8.151231	0.318881
H99	0.165393	7.140287	-4.509220
H100	1.156344	-0.291367	9.255562
H101	5.126751	1.145773	8.446530
H102	0.725533	-0.484246	6.820900
H103	4.680653	0.965382	6.003329
H104	1.617765	-0.201645	4.652647
H105	2.749308	-5.776969	4.729187
H106	3.908122	-5.061498	5.853721
H107	0.861236	-5.231083	6.290354
H108	7.633729	1.510306	2.188715
H109	6.530539	0.218251	0.308350
H110	9.380012	-0.655724	0.909564
H111	8.458450	4.112993	-0.987519
H112	2.613520	4.885711	-3.986868
H113	1.196910	2.217296	-4.655927
H114	-4.406212	5.310409	-3.936400
H115	-4.697888	5.462284	-1.475614
H116	-6.265133	5.180837	-2.243183
H117	-5.423844	3.842060	-1.446834
H118	-2.820753	3.398857	-4.140064
H119	-3.383224	2.754093	-2.610694
H120	-5.698162	2.538433	-3.705031
H121	-1.922797	0.378820	-3.150448
H122	-2.022475	-1.334385	-2.833877
H123	-4.274193	0.353275	-4.108520
H124	-2.265363	-1.605933	-7.120523
H125	1.722584	-3.451494	-5.910470
H126	3.341133	-2.774324	-5.925520
H127	2.543144	-3.230300	-3.667254
H128	3.249411	-1.107934	-2.621249
H129	2.993352	-0.230681	-4.122314
H130	4.261962	-1.469448	-4.023164
H131	0.804164	-1.814879	-2.568586
H132	0.461820	-1.014669	-4.097915
H133	0.107085	-2.740001	-3.912159
H134	1.971838	-0.426805	-6.106651
H135	-2.058050	-4.273770	-3.470365
H136	-1.154795	-5.384574	-4.497310
H137	-2.332911	-6.075140	-1.761909
H138	-0.914596	-8.134726	-1.896695
H139	-2.122881	-8.125748	-3.178240
H140	-0.442960	-7.708828	-3.547650
H141	0.014265	-5.994418	-0.938884
H142	-0.474542	-4.423574	-1.598481
H143	0.575195	-5.491783	-2.540200
H144	-3.223961	-6.828865	-4.699927
H145	-6.964640	-5.582430	-2.847188
H146	-5.023944	-3.468015	1.054887
H147	-6.369162	-3.486162	3.144228
H148	-4.662042	-3.411323	3.554143
H149	-5.535110	-4.943048	3.696170
H150	-3.711156	-5.672098	0.554535
H151	-3.660653	-5.891240	2.295816
H152	-2.564372	-3.477422	0.734269
H153	-1.584921	-4.756988	1.477148
H154	-2.479429	-3.617103	2.492212
H155	-6.259438	-6.232836	1.504911
H156	-1.790513	-2.264787	6.889374
H157	-1.113710	-3.143821	5.525188
H158	-3.113323	-4.688131	5.521735
H159	8.628659	0.753902	-5.272535
H160	9.450338	-1.096295	-3.798840
H161	-7.529695	0.035099	1.394263
H162	-7.135612	1.741527	1.739991
H163	-7.174466	1.151736	0.070300
H164	-2.996244	-0.488942	2.883752
C165	0.133153	-1.725455	0.890390
C166	0.408391	-0.451632	1.565628
O167	-0.036808	-0.881159	-0.262230
C168	1.851324	-0.084028	1.414752
C169	1.457774	-2.466646	0.815749
C170	2.525443	-1.338906	0.786257
H171	-0.379793	0.238068	1.835684
H172	1.942675	0.803237	0.780291
H173	3.428495	-1.626451	1.330602
H174	2.254500	0.184650	2.394454
H175	2.818092	-1.118877	-0.241799
H176	1.559847	-3.092404	1.708072
H177	1.500252	-3.114264	-0.064033
H178	-0.759783	-2.286170	1.159475
H179	1.763625	3.421689	-5.805654
H180	7.531792	0.481786	-3.908708
H181	9.108176	-1.681590	-5.424633
H182	10.174563	3.876047	-1.396226
H183	8.940023	2.772774	-2.048999
H184	9.998820	0.780373	1.729634
H185	3.362881	0.505870	10.086517
H186	2.065871	-6.261290	7.087802
H187	2.003330	-4.528283	7.448108
H188	-2.380733	-4.663385	7.133942
H189	-3.858553	-3.729090	6.802651
H190	-7.188035	-4.752924	1.281487
H191	-7.140773	-7.353415	-2.854594
H192	-3.457322	-5.184377	-5.282228
H193	2.410282	-1.403559	-7.502681
H194	-1.708612	-0.571480	-8.445049
H195	-4.349046	-1.396914	-3.830210
H196	-3.102799	-0.153408	1.128488
H197	-4.993246	0.549869	0.190246
H198	0.344466	8.881266	-4.180991
H199	1.630293	7.742408	-3.704371
H200	-4.760195	10.912259	-0.431071
H201	-3.953273	9.572933	-1.281105
H202	-6.200218	6.886692	1.463819
H203	7.102145	-0.112492	-5.523446
H204	-1.131184	6.950252	-0.274861
H205	-2.492857	0.156910	-7.027232
H206	-6.462773	3.966855	-4.422385
H207	-5.041806	3.241628	-5.190640

H208	-3.154467	-0.733397	-4.962840
H209	2.937409	2.518917	-4.836847
H210	4.327293	-2.857446	5.569492
H211	3.307752	-1.751538	4.714733
H212	9.682767	2.810441	0.820318
H213	0.119349	-2.191049	-7.566379
H214	-1.002662	7.894620	-2.330886
H215	-6.102328	9.127690	0.718189
H216	-4.904273	-0.509306	4.114832
H217	-6.397964	0.100552	3.449117
H218	-5.801649	-4.675916	-0.983811
H219	-4.936512	-7.540562	-3.528325
H220	-1.081304	-0.682952	-0.443429
H221	-0.551873	-1.123685	3.982121
H222	0.810573	-1.938118	3.792658
C223	8.153493	-0.191841	3.394396
H224	8.385193	-1.256213	3.247122
H225	9.062019	0.260210	3.814664
H226	6.838574	-1.279162	1.190145
S227	2.307501	3.431610	-2.082272
H228	2.319793	4.636195	-1.470428
H229	0.898467	4.538569	-3.720717
S230	-5.205445	4.699234	3.113721
H231	-4.478255	3.572478	2.979719
C232	7.709629	-2.339758	-3.938377
C233	7.345332	-2.303817	-2.583469
H234	7.752729	-1.522738	-1.945901
C235	7.171514	-3.348780	-4.747890
H236	7.448905	-3.392659	-5.798879
C237	6.468949	-3.254348	-2.059028
H238	6.198631	-3.213511	-1.006947
C239	5.940499	-4.257354	-2.875226
H240	5.262900	-4.999957	-2.462981
C241	6.294351	-4.301136	-4.224387
H242	5.894785	-5.080319	-4.868152
C243	-2.174369	4.542626	-2.436324
H244	-2.497413	4.827342	-1.430181
H245	-1.228773	3.999514	-2.337248
H246	-1.969194	5.469334	-2.986110
H247	5.749815	-0.057608	1.870838
C248	7.018176	-0.049978	4.412957
H249	6.107983	-0.563052	4.087762
H250	7.308342	-0.470486	5.381435
H251	6.763481	1.004229	4.572661

TS attack at C2:

C1	-5.255907	6.788452	1.801984
C2	-4.732674	7.667929	0.666163
O3	-3.693546	7.422248	0.055225
C4	-4.743753	5.353040	1.699729
N5	-5.496749	8.766252	0.409599
C6	-5.144331	9.757468	-0.590057
C7	-0.239017	7.092825	0.028514
C8	0.499477	7.198308	-1.300266
O9	1.689692	6.907618	-1.430993
C10	0.342986	5.999073	0.930921
C11	-0.288697	5.918890	2.333790
C12	0.165452	4.638932	3.050508
C13	0.024545	7.153240	3.193398
N14	-0.246894	7.691475	-2.330603
C15	0.303162	7.908373	-3.656001
C16	3.304251	0.302405	8.984572
C17	2.073594	-0.155495	8.506034
C18	4.271691	0.719375	8.066255
C19	1.811069	-0.219306	7.134661
C20	4.022910	0.665002	6.695687
C21	2.793307	0.186699	6.223257
O22	2.641165	0.095518	4.864377
C23	2.117093	-5.385286	6.537885
C24	3.255408	-4.931609	5.616239
C25	2.897284	-3.627606	4.925049
N26	3.812497	-2.637366	4.951414
O27	1.802761	-3.512717	4.349835
C28	9.155614	0.542319	1.024252
C29	8.997209	1.309595	-0.269250
O30	8.583730	0.800037	-1.309762
C31	7.900160	0.600176	1.931777
C32	6.681717	-0.034221	1.245916
N33	9.320649	2.628595	-0.166542
C34	9.127943	3.555802	-1.253358
C35	7.848574	0.265786	-4.936566
C36	8.581462	-1.056917	-4.653408
C37	1.799573	3.076405	-4.782620
C38	1.741192	4.172481	-3.723099
C39	-5.648869	3.378778	-4.069002
C40	-4.716017	4.353702	-3.337539
C41	-5.473375	5.062633	-2.205243
C42	-3.457240	3.612819	-2.844993
C43	-6.906500	0.657304	1.222510
N44	-5.480280	0.395107	1.206286
C45	-4.765607	0.002019	2.272627
N46	-5.330083	-0.150149	3.490305
N47	-3.453764	-0.185646	2.141089
C48	-3.727498	-0.618514	-3.964234
C49	-2.664909	-0.420197	-2.875873
C50	-3.223553	-0.152112	-1.476375
O51	-4.383809	0.287106	-1.344831
O52	-2.442753	-0.384186	-0.469629
C53	-1.923706	-0.594433	-7.329578
C54	-0.565217	-0.374785	-6.690335
O55	-0.295678	0.626614	-6.033555
N56	0.326589	-1.379380	-6.930274
C57	1.743290	-1.285629	-6.620899
C58	2.282361	-2.412886	-5.716581
C59	2.226270	-2.161208	-4.193596
C60	3.199981	-1.051979	-3.767046
C61	0.807140	-1.880511	-3.681220
C62	-3.207449	-5.826640	-4.424354
C63	-4.391162	-5.634434	-3.484819
O64	-4.698039	-4.533658	-3.023504
C65	-1.894974	-5.308646	-3.810216
C66	-1.364037	-6.119979	-2.610513
C67	-0.944654	-7.545054	-3.002134
C68	-0.194819	-5.380529	-1.944340
N69	-5.117473	-6.752605	-3.213003
C70	-6.397882	-6.679178	-2.529218
C71	-6.349504	-6.768102	-0.997560

O72	-6.608801	-7.814778	-0.410971
N73	-6.057327	-5.593909	-0.385808
C74	-6.087753	-5.447779	1.061816
C75	-5.021666	-4.476823	1.589009
C76	-3.599004	-4.985515	1.286312
C77	-5.265683	-4.243194	3.085608
C78	-2.502783	-3.923192	1.418629
C79	-2.706084	-4.276729	6.367551
C80	-1.895668	-3.027270	5.987355
C81	-2.635846	-2.044446	5.061665
O82	-3.868436	-1.897126	5.203813
O83	-1.932817	-1.405674	4.193042
O84	0.548262	-1.218395	3.659522
H85	-5.083024	4.890814	0.771920
H86	-3.653542	5.349561	1.707775
H87	-4.922447	7.232223	2.749205
H88	-5.882894	9.789668	-1.398881
H89	0.216766	5.031737	0.428431
H90	1.424665	6.154249	1.020886
H91	-1.380966	5.865229	2.209447
H92	-0.297899	4.548477	4.038787
H93	-0.095788	3.745200	2.473552
H94	1.253126	4.635828	3.191918
H95	-0.436655	7.068502	4.183487
H96	-0.342155	8.079430	2.739697
H97	1.106713	7.259089	3.338972
H98	-0.146037	8.074383	0.512260
H99	-0.059424	7.164803	-4.376032
H100	1.304157	-0.473386	9.204065
H101	5.230055	1.092104	8.417309
H102	0.855383	-0.586186	6.770156
H103	4.766889	0.991588	5.976456
H104	1.742819	-0.212460	4.591053
H105	3.415513	-5.679227	4.829948
H106	4.197626	-4.837582	6.167034
H107	1.187094	-5.468594	5.972232
H108	7.673223	1.661281	2.118242
H109	6.512430	0.392938	0.254984
H110	9.369328	-0.499130	0.763123
H111	8.324052	4.270806	-1.041543
H112	2.358725	5.026781	-4.010842
H113	1.152829	2.229177	-4.542319
H114	-4.393576	5.123040	-4.057302
H115	-4.847641	5.788672	-1.677635
H116	-6.348222	5.596200	-2.595865
H117	-5.837990	4.329308	-1.473612
H118	-3.014371	3.085863	-3.701041
H119	-3.760858	2.828587	-2.137781
H120	-5.949599	2.561754	-3.402003
H121	-2.015102	0.427785	-3.131619
H122	-2.001997	-1.289336	-2.813485
H123	-4.407554	0.235890	-4.000478
H124	-2.339776	-1.571887	-7.065309
H125	1.743296	-3.340476	-5.956549
H126	3.329949	-2.593697	-5.989371
H127	2.577550	-3.092485	-3.724902
H128	3.207051	-0.941240	-2.677897
H129	2.914119	-0.078761	-4.180780
H130	4.224479	-1.273285	-4.083231
H131	0.793355	-1.807978	-2.588916
H132	0.422139	-0.934444	-4.074039
H133	0.111709	-2.674585	-3.976617
H134	1.890297	-0.304962	-6.165050
H135	-2.068566	-4.273639	-3.496186
H136	-1.124338	-5.279942	-4.592285
H137	-2.173655	-6.195932	-1.869880
H138	-0.567080	-8.093889	-2.133115
H139	-1.774472	-8.127663	-3.415643
H140	-0.146121	-7.523664	-3.754231
H141	0.174485	-5.933132	-1.073456
H142	-0.493372	-4.381461	-1.611407
H143	0.643126	-5.261699	-2.642162
H144	-3.117015	-6.872498	-4.737152
H145	-6.877463	-5.742395	-2.828995
H146	-5.156387	-3.516699	1.065034
H147	-6.285302	-3.882642	3.265021
H148	-4.586250	-3.507863	3.518678
H149	-5.148543	-5.179927	3.644735
H150	-3.578180	-5.383941	0.264887
H151	-3.380416	-5.833942	1.950132
H152	-2.669679	-3.097580	0.716788
H153	-1.521115	-4.357861	1.197349
H154	-2.459699	-3.497682	2.425812
H155	-5.951410	-6.446640	1.487311
H156	-1.647681	-2.465670	6.899657
H157	-0.942489	-3.303340	5.524302
H158	-2.897382	-4.903701	5.491025
H159	8.528872	0.999809	-5.382415
H160	9.397035	-0.860055	-3.948079
H161	-7.494338	-0.222744	1.519967
H162	-7.160772	1.492544	1.886994
H163	-7.204091	0.926448	0.208340
H164	-2.915350	-0.581717	2.927212
C165	0.348944	-1.461400	1.517978
C166	0.379858	-0.089394	0.984293
O167	-0.011227	-0.720402	-0.248218
C168	1.843910	0.314069	0.933015
C169	1.657355	-2.139836	1.228016
C170	2.579540	-1.022374	0.661706
H171	-0.339257	0.644937	1.348944
H172	2.029283	1.069050	0.164281
H173	3.574121	-1.044200	1.113436
H174	2.122206	0.729206	1.907890
H175	2.703384	-1.162750	-0.413335
H176	2.035536	-2.601149	2.142947
H177	1.499866	-2.957564	0.517674
H178	-0.579628	-1.974536	1.725331
H179	1.459779	3.472346	-5.745781
H180	7.453528	0.690477	-4.009298
H181	9.041932	-1.426252	-5.577934
H182	10.046256	4.114678	-1.464025
H183	8.854355	2.975713	-2.134717
H184	10.020531	0.923729	1.581932
H185	3.499140	0.344837	10.051196
H186	2.345505	-6.357948	6.982138
H187	1.958598	-4.669824	7.350568
H188	-2.168795	-4.881036	7.105415
H189	-3.672666	-3.985001	6.784296
H190	-7.082465	-5.100790	1.376781
H191	-7.014109	-7.516398	-2.862289

H192	-3.436627	-5.244883	-5.325086
H193	2.303646	-1.286082	-7.565746
H194	-1.839831	-0.550040	-8.421546
H195	-4.322784	-1.515212	-3.764785
H196	-3.059282	-0.192339	1.189097
H197	-5.002730	0.404357	0.281203
H198	0.046164	8.907149	-4.023998
H199	1.386674	7.819914	-3.578304
H200	-5.064263	10.754890	-0.145419
H201	-4.177864	9.472807	-1.005250
H202	-6.352492	6.805939	1.816040
H203	7.012434	0.114292	-5.627443
H204	-1.310248	6.933201	-0.135682
H205	-2.604656	0.188018	-6.997835
H206	-6.557477	3.879091	-4.422296
H207	-5.153784	2.929469	-4.936649
H208	-3.256528	-0.733611	-4.942231
H209	2.822791	2.708486	-4.911999
H210	4.618931	-2.710927	5.551426
H211	3.535207	-1.701338	4.657376
H212	9.621542	2.981678	0.728566
H213	0.025219	-2.146628	-7.513141
H214	-1.234348	7.831612	-2.174854
H215	-6.350802	8.888952	0.932082
H216	-4.803080	-0.711983	4.185609
H217	-6.335280	-0.186453	3.544630
H218	-5.796273	-4.808865	-0.973145
H219	-4.809606	-7.644814	-3.566340
H220	-1.057635	-0.607159	-0.415022
H221	-0.428638	-1.298671	3.967089
H222	0.976295	-2.074494	3.909885
C223	8.219768	-0.055226	3.290269
H224	8.454788	-1.116110	3.124387
H225	9.135010	0.397998	3.694360
H226	6.832196	-1.113441	1.117767
S227	2.366374	3.555620	-2.093329
H228	2.308842	4.751319	-1.466224
H229	0.714582	4.522509	-3.588346
S230	-5.377983	4.383863	3.138627
H231	-4.682133	3.256604	2.890659
C232	7.672702	-2.126525	-4.083454
C233	7.325359	-2.114804	-2.723687
H234	7.729942	-1.336957	-2.080420
C235	7.138614	-3.131733	-4.900342
H236	7.403229	-3.157061	-5.955224
C237	6.469043	-3.084807	-2.201758
H238	6.211615	-3.062370	-1.145896
C239	5.944254	-4.083675	-3.025388
H240	5.282451	-4.841577	-2.615304
C241	6.281668	-4.103679	-4.379361
H242	5.885081	-4.879524	-5.028966
C243	-2.386034	4.505714	-2.211137
H244	-2.739847	4.990147	-1.295433
H245	-1.493985	3.923921	-1.955611
H246	-2.073589	5.299315	-2.901743
H247	5.772733	0.113149	1.836247
C248	7.107092	0.065630	4.336216
H249	6.199838	-0.464062	4.030210
H250	7.427084	-0.350079	5.297418
H251	6.836075	1.114661	4.503045

4.5 Mutant *S1*

The following atoms were kept fixed in the geometry optimizations of reactant and transition state structures: 1, 9, 10, 18, 19, 26, 31, 39, 40, 45, 50, 54, 59, 64, 68, 78, 82, 86.

Reactant:

C1	-6.475280	5.491149	1.257834
C2	-6.077078	6.512917	0.203644
O3	-4.967522	6.475093	-0.328592
C4	-7.962600	5.283908	1.561942
C5	-8.238389	4.280310	2.692211
S6	-7.680069	2.548018	2.403676
C7	-6.083249	2.503789	3.297574
N8	-6.992701	7.476932	-0.081962
C9	-6.709124	8.555684	-1.001215
C10	-1.461626	6.897523	0.120984
C11	-0.628327	7.133556	-1.131581
O12	0.574240	6.882673	-1.184298
C13	-1.168774	5.537514	0.769547
C14	-2.022514	5.238134	2.016354
C15	-1.820339	3.783334	2.465138
C16	-1.731557	6.202708	3.176369
N17	-1.316007	7.687543	-2.175826
C18	-0.687866	8.017217	-3.440237
C19	2.373250	0.448463	9.208279
C20	1.342705	-0.172453	8.498069
C21	3.407053	1.066872	8.500141
C22	1.342664	-0.192889	7.102622
C23	3.418634	1.057943	7.106581
C24	2.387291	0.423957	6.398654
O25	2.473732	0.400103	5.038998
C26	2.666162	-5.210313	6.444601
C27	3.794820	-4.528980	5.661963
C28	3.289061	-3.260364	4.992768
N29	4.024147	-2.139283	5.151541
O30	2.246877	-3.285895	4.323235
C31	8.907702	2.317438	2.033978
C32	9.011650	3.174721	0.792193
O33	9.512098	2.777916	-0.260887
C34	7.479105	2.141539	2.593245
C35	6.464497	1.483547	1.636028
C36	6.820129	0.021082	1.329525
C37	5.043350	1.591542	2.209111
N38	8.529683	4.435745	0.949291
C39	8.516462	5.390913	-0.127051
C40	8.365059	2.130126	-4.047686
C41	9.273718	0.950643	-3.681816
C42	8.782339	0.235018	-2.421507
S43	9.854408	-1.131960	-1.819100
C44	11.303315	-0.174904	-1.234639
C45	1.888809	3.656977	-4.538194
C46	1.293304	3.727966	-3.125161
C47	2.143859	2.903632	-2.138707
C48	-0.176007	3.280128	-3.150453
C49	1.752130	3.067759	-0.666021
C50	-5.494150	2.417791	-4.728873
C51	-5.708992	3.259122	-3.463036
C52	-6.570635	2.498863	-2.444770
C53	-4.369962	3.680297	-2.840882
C54	-6.751680	-0.802384	0.274853
N55	-5.314834	-0.714871	0.493217
C56	-4.675234	-1.042256	1.632931
N57	-5.344881	-1.451052	2.728230
N58	-3.352613	-0.906789	1.709458
C59	-2.814910	-1.112258	-4.525347
C60	-2.114727	-1.616972	-3.263041
C61	-2.667466	-0.998058	-1.995593
O62	-3.745622	-0.425132	-1.937476
O63	-1.939830	-1.128224	-0.879442
C64	-0.687683	-0.532102	-7.642732
C65	0.472601	-0.268454	-6.704022
O66	0.359315	0.413988	-5.688344
N67	1.649037	-0.828799	-7.106252
C68	2.939475	-0.513808	-6.515680
C69	3.686483	-1.730269	-5.924714
C70	3.480560	-1.995297	-4.416537
C71	4.160963	-0.921948	-3.552932
C72	2.001713	-2.153265	-4.037105
C73	-0.775547	-6.507158	-4.454675
C74	-2.207953	-6.409885	-3.933957
O75	-2.728167	-5.334526	-3.622416
C76	0.148351	-5.448323	-3.863066
N77	-2.895298	-7.579476	-3.918223
C78	-4.323868	-7.658019	-3.632084
C79	-4.686612	-7.737773	-2.140670
O80	-5.293258	-8.704324	-1.688709
N81	-4.316557	-6.665155	-1.400461
C82	-4.680227	-6.599527	0.009436
C83	-4.149863	-5.363868	0.737346
C84	-2.617348	-5.345666	0.805960
C85	-4.767811	-5.322718	2.142447
C86	-2.231431	-5.083455	5.724571
C87	-1.688907	-3.671017	5.457257
C88	-2.508919	-2.835842	4.457678
O89	-3.758962	-2.971837	4.444003
O90	-1.860564	-2.021576	3.712469
O91	0.712305	-1.062391	3.770508
H92	-8.428619	6.229318	1.879313
H93	-8.486049	4.959313	0.654970
H94	-7.789767	4.619595	3.632067
H95	-9.317482	4.215041	2.858870
H96	-6.212761	2.843472	4.328232
H97	-5.766995	1.458226	3.314512
H98	-5.314329	3.098674	2.801179
H99	-6.029599	4.545044	0.938391
H100	-7.544115	8.704317	-1.692947
H101	-1.352027	4.751916	0.025578

H102	-0.102709	5.481363	1.018784	H162	2.753188	0.248245	-5.756891
H103	-3.075901	5.362196	1.724033	H163	-0.351420	-4.480435	-3.895078
H104	-2.440369	3.542733	3.336249	H164	1.071021	-5.387943	-4.443782
H105	-2.076540	3.078599	1.666708	H165	-0.372923	-7.514773	-4.298223
H106	-0.776295	3.599953	2.745782	H166	-4.798180	-6.775915	-4.074340
H107	-2.344789	5.960449	4.051751	H167	-4.483513	-4.469896	0.186995
H108	-1.936810	7.244807	2.911191	H168	-5.861525	-5.265671	2.092772
H109	-0.680315	6.137408	3.482824	H169	-4.404486	-4.474442	2.727353
H110	-1.207057	7.707554	0.816766	H170	-4.514104	-6.233131	2.699454
H111	-1.033095	7.363363	-4.250304	H171	-2.153866	-5.352767	-0.186100
H112	0.528556	-0.653943	9.033265	H172	-2.247973	-6.224045	1.348863
H113	4.212128	1.562580	9.036265	H173	-4.312171	-7.505482	0.507752
H114	0.551745	-0.688706	6.548498	H174	-1.682506	-3.102050	6.398130
H115	4.214054	1.540448	6.546511	H175	-0.650806	-3.703260	5.111827
H116	1.667634	-0.027996	4.619993	H176	-2.177844	-5.703712	4.823745
H117	4.149761	-5.195540	4.866555	H177	8.720215	2.641903	-4.948410
H118	4.652980	-4.317294	6.309247	H178	10.291351	1.322215	-3.515631
H119	1.816866	-5.401111	5.785938	H179	10.969745	0.697877	-0.668074
H120	7.082724	3.116388	2.910554	H180	11.882747	-0.835733	-0.585832
H121	7.543375	1.542824	3.511835	H181	-7.127409	-1.825824	0.405106
H122	6.484555	2.042379	0.688912	H182	-7.306264	-0.128661	0.937297
H123	6.084685	-0.420630	0.648816	H183	-6.949225	-0.499411	-0.753339
H124	6.820366	-0.579174	2.248076	H184	-2.827511	-1.281920	2.535436
H125	7.800569	-0.084941	0.855934	C185	0.262044	-2.841753	0.944705
H126	4.755130	2.634626	2.379475	C186	1.035113	-1.685802	0.498739
H127	4.309328	1.154533	1.524030	O187	0.279658	-2.479862	-0.490205
H128	4.949978	1.064565	3.166263	C188	2.507703	-2.020380	0.543532
H129	9.343942	1.347869	1.785195	C189	1.201977	-3.981880	1.266393
H130	7.497721	5.604005	-0.473128	C190	2.545739	-3.567483	0.612357
H131	1.322387	4.772985	-2.785551	H191	0.679562	-0.666569	0.614472
H132	-0.644904	3.348195	-2.163793	H192	3.070060	-1.609306	-0.300862
H133	-0.761459	3.907437	-3.832366	H193	3.405680	-3.930731	1.180307
H134	-0.261722	2.245759	-3.504426	H194	2.904771	-1.572749	1.462315
H135	2.096273	1.841443	-2.422486	H195	2.620250	-3.985111	-0.394749
H136	3.194537	3.202476	-2.257446	H196	1.302237	-4.038512	2.354351
H137	1.737698	4.126559	-0.385799	H197	0.831731	-4.949536	0.912812
H138	0.757344	2.659978	-0.458565	H198	-0.701137	-2.740647	1.434838
H139	2.460147	2.552044	-0.007597	H199	1.348651	4.315143	-5.228780
H140	1.817113	2.638746	-4.938574	H200	8.337243	2.860395	-3.232232
H141	-6.251859	4.171850	-3.751637	H201	9.332423	0.236379	-4.511909
H142	-6.754727	3.097608	-1.545990	H202	8.983218	6.334545	0.176327
H143	-7.542410	2.219741	-2.868649	H203	9.085246	4.964672	-0.953314
H144	-6.061687	1.578915	-2.132319	H204	9.542409	2.760773	2.812639
H145	-4.515696	4.346903	-1.984074	H205	2.366162	0.457336	10.293678
H146	-3.742125	4.205819	-3.569278	H206	3.005700	-6.160969	6.865485
H147	-3.812410	2.800114	-2.498486	H207	2.321075	-4.577961	7.268465
H148	-4.957043	1.492621	-4.488063	H208	-1.655569	-5.580799	6.511704
H149	-1.035824	-1.431144	-3.310160	H209	-3.279214	-5.034065	6.030454
H150	-2.242331	-2.705170	-3.163059	H210	-5.774827	-6.637251	0.091443
H151	-2.546071	-0.072938	-4.729374	H211	-4.727488	-8.554639	-4.103173
H152	-0.647211	-1.529504	-8.091881	H212	-0.825774	-6.359906	-5.542757
H153	3.398689	-2.623983	-6.494828	H213	3.564115	-0.048258	-7.288797
H154	4.760526	-1.592278	-6.101786	H214	-0.670693	0.203629	-8.454919
H155	3.985847	-2.948749	-4.205659	H215	-3.899438	-1.176800	-4.415492
H156	4.088443	-1.176452	-2.490280	H216	-2.834999	-0.741017	0.853578
H157	3.695848	0.061855	-3.681385	H217	-4.732190	-0.459149	-0.306746
H158	5.224647	-0.826072	-3.798850	H218	-0.888352	9.057422	-3.720480
H159	1.896229	-2.437228	-2.984554	H219	0.386326	7.879206	-3.313107
H160	1.451683	-1.219113	-4.189911	H220	-6.516129	9.499985	-0.477296
H161	1.519509	-2.926774	-4.644532	H221	-5.818486	8.283082	-1.566988

H222	-5.943974	5.780125	2.175765
H223	7.336580	1.800297	-4.236207
H224	11.937508	0.145589	-2.064640
H225	8.665859	0.951753	-1.603722
H226	7.808219	-0.234005	-2.601843
H227	-2.533067	6.981827	-0.092593
H228	-1.625908	-0.414797	-7.102799
H229	-6.447304	2.140296	-5.193821
H230	-4.903319	2.962226	-5.473904
H231	-2.517455	-1.720384	-5.383175
H232	2.942398	3.959948	-4.546084
H233	4.768566	-2.119562	5.830585
H234	3.621053	-1.242763	4.876536
H235	8.094058	4.680694	1.824801
H236	1.650220	-1.338969	-7.977311
H237	-2.317341	7.772644	-2.087988
H238	-7.873500	7.476316	0.406410
H239	-4.785184	-1.966674	3.448422
H240	-6.312119	-1.713382	2.621721
H241	-3.858006	-5.887971	-1.863950
H242	-2.393493	-8.441571	-4.068067
H243	-1.100280	-1.673045	-0.940730
H244	-0.235415	-1.326609	3.852382
H245	1.219468	-1.890656	3.897769
S246	0.570253	-5.828101	-2.104981
H247	0.509357	-4.563302	-1.641783
H248	-2.272458	-4.455944	1.340253

TS attack at C1:

C1	-6.340443	5.553233	1.420646
C2	-5.918159	6.606645	0.408899
O3	-4.802543	6.569365	-0.110375
C4	-7.806615	5.483770	1.858554
C5	-8.081838	4.462909	2.973354
S6	-7.738933	2.697242	2.574995
C7	-6.077279	2.454526	3.305005
N8	-6.820003	7.588964	0.142719
C9	-6.520225	8.685548	-0.748603
C10	-1.303420	6.890391	0.303748
C11	-0.463688	7.169663	-0.935303
O12	0.743406	6.938594	-0.982977
C13	-1.053074	5.486341	0.872679
C14	-1.919400	5.138109	2.097713
C15	-1.743087	3.658879	2.471863
C16	-1.620282	6.036840	3.307348
N17	-1.151171	7.736290	-1.972310
C18	-0.519883	8.096201	-3.227089
C19	2.432486	0.106726	9.186340
C20	1.336083	-0.439787	8.513364
C21	3.479631	0.650062	8.436634
C22	1.283344	-0.462458	7.116664
C23	3.439540	0.637854	7.043090
C24	2.342564	0.075786	6.376554
O25	2.393402	0.049230	5.006395
C26	2.600775	-5.474996	6.260333
C27	3.705749	-4.846796	5.401539
C28	3.226792	-3.535754	4.804609
N29	3.990887	-2.443269	5.004294
O30	2.166963	-3.500662	4.159562
C31	8.977748	2.049153	2.041465
C32	9.082981	2.939838	0.824015
O33	9.594843	2.570019	-0.233231
C34	7.578283	1.932845	2.679196
C35	6.468122	1.382101	1.761713
C36	6.741051	-0.064097	1.322216
C37	5.103956	1.491920	2.458589
N38	8.602307	4.196347	1.010011
C39	8.641137	5.190756	-0.028894
C40	8.409656	2.047767	-4.040759
C41	9.261619	0.819861	-3.698531
C42	8.743292	0.110882	-2.445071
S43	9.755445	-1.308786	-1.861543
C44	11.250902	-0.425161	-1.279663
C45	1.963962	3.718295	-4.460337
C46	1.406561	3.809097	-3.032695
C47	2.307252	3.035629	-2.049314
C48	-0.049819	3.321422	-3.005284
C49	1.960207	3.240810	-0.570262
C50	-5.443227	2.634142	-4.655760
C51	-5.609103	3.350672	-3.308065
C52	-6.489575	2.523245	-2.360690
C53	-4.247688	3.649506	-2.663837
C54	-6.747834	-0.702585	0.258753
N55	-5.305104	-0.706403	0.422645
C56	-4.658198	-1.043980	1.548182
N57	-5.323390	-1.375843	2.680166
N58	-3.329039	-0.998729	1.574909
C59	-2.835358	-0.953576	-4.564692
C60	-2.158556	-1.490398	-3.298930
C61	-2.737401	-0.959966	-1.987511
O62	-3.866064	-0.438808	-1.963887
O63	-2.026573	-1.130931	-0.913332
C64	-0.707983	-0.327011	-7.672981
C65	0.481932	-0.021615	-6.787980
O66	0.427967	0.795895	-5.873818
N67	1.614502	-0.707068	-7.117943
C68	2.922810	-0.414232	-6.560966
C69	3.589176	-1.608197	-5.841107
C70	3.343451	-1.718883	-4.318955
C71	4.090135	-0.622295	-3.543727
C72	1.853290	-1.734111	-3.950566
C73	-0.963890	-6.247427	-4.710389
C74	-2.391917	-6.194598	-4.171267
O75	-2.934096	-5.139820	-3.834971
C76	-0.080231	-5.128173	-4.169670
N77	-3.052426	-7.381351	-4.164238
C78	-4.473441	-7.490578	-3.853234
C79	-4.812308	-7.630294	-2.360740
O80	-5.441417	-8.599884	-1.947624
N81	-4.393044	-6.603061	-1.579655
C82	-4.795242	-6.530265	-0.181408
C83	-3.927235	-5.601387	0.672529
C84	-2.479022	-6.097656	0.772090
C85	-4.574820	-5.464160	2.057546
C86	-2.295787	-5.228813	5.564616
C87	-1.669284	-3.849376	5.313793
C88	-2.480057	-2.931313	4.381394

O89	-3.726364	-3.033860	4.366151
O90	-1.820977	-2.079285	3.679983
O91	0.643429	-1.402488	3.384422
H92	-8.134718	6.457825	2.252530
H93	-8.443474	5.254294	0.995747
H94	-7.517486	4.714624	3.877793
H95	-9.142383	4.499448	3.238924
H96	-6.075998	2.747117	4.358115
H97	-5.861944	1.385160	3.242742
H98	-5.303571	3.002115	2.763761
H99	-6.035867	4.591254	0.997104
H100	-7.305135	8.803803	-1.502795
H101	-1.256422	4.751459	0.083571
H102	0.010030	5.385185	1.120958
H103	-2.968573	5.293146	1.806582
H104	-2.375075	3.383158	3.323836
H105	-2.002531	3.000536	1.635876
H106	-0.703805	3.447422	2.752227
H107	-2.240241	5.757626	4.166920
H108	-1.810847	7.093547	3.094999
H109	-0.570936	5.942368	3.613403
H110	-1.020234	7.650574	1.043165
H111	-0.848719	7.449554	-4.049497
H112	0.508052	-0.860359	9.077073
H113	4.336624	1.090481	8.938795
H114	0.431309	-0.897893	6.601507
H115	4.245199	1.066234	6.454541
H116	1.577619	-0.318781	4.591664
H117	3.951589	-5.513922	4.567160
H118	4.623432	-4.703346	5.982163
H119	1.696303	-5.614936	5.665238
H120	7.260764	2.910269	3.069808
H121	7.662437	1.285032	3.562462
H122	6.433520	2.010435	0.859947
H123	5.929515	-0.433811	0.686675
H124	6.807754	-0.729434	2.192624
H125	7.668866	-0.163939	0.751831
H126	4.869396	2.526849	2.730208
H127	4.298986	1.131077	1.810005
H128	5.078195	0.894914	3.378717
H129	9.342828	1.065059	1.740446
H130	7.635811	5.460984	-0.373430
H131	1.417859	4.862748	-2.719086
H132	-0.490179	3.388901	-2.005611
H133	-0.672776	3.922036	-3.677531
H134	-0.119478	2.281161	-3.343827
H135	2.274284	1.964082	-2.296166
H136	3.346660	3.352647	-2.212546
H137	1.931682	4.307738	-0.324322
H138	0.981376	2.819855	-0.318342
H139	2.700581	2.760681	0.080619
H140	1.910905	2.691100	-4.837374
H141	-6.115159	4.310029	-3.495305
H142	-6.628449	3.025997	-1.396753
H143	-7.481775	2.340698	-2.790216
H144	-6.018746	1.552103	-2.167787
H145	-4.358476	4.259350	-1.760362
H146	-3.593500	4.195498	-3.353087
H147	-3.740276	2.716546	-2.391361
H148	-4.953554	1.663082	-4.517917
H149	-1.081941	-1.288505	-3.311140
H150	-2.272680	-2.583187	-3.254863
H151	-2.590220	0.100216	-4.725318
H152	-0.681243	-1.337220	-8.093221
H153	3.260271	-2.535127	-6.330682
H154	4.672247	-1.548098	-6.007904
H155	3.774640	-2.682892	-4.012641
H156	3.963899	-0.756368	-2.463741
H157	3.715368	0.377513	-3.789164
H158	5.164946	-0.641304	-3.758064
H159	1.719961	-1.917306	-2.879144
H160	1.372708	-0.780493	-4.191026
H161	1.320219	-2.520736	-4.495057
H162	2.788307	0.433745	-5.887144
H163	-0.623628	-4.184984	-4.222611
H164	0.830129	-5.047888	-4.767332
H165	-0.515418	-7.230600	-4.525091
H166	-4.966821	-6.598308	-4.251978
H167	-3.920903	-4.606815	0.199680
H168	-5.612448	-5.119811	1.976247
H169	-4.044796	-4.752390	2.693848
H170	-4.591376	-6.432170	2.573518
H171	-2.005397	-6.176829	-0.211180
H172	-2.443967	-7.087981	1.242561
H173	-4.769797	-7.548973	0.218853
H174	-1.576329	-3.312171	6.268941
H175	-0.652305	-3.941959	4.917761
H176	-2.325370	-5.822117	4.645277
H177	8.784070	2.554437	-4.936463
H178	10.297405	1.138584	-3.534601
H179	10.963467	0.443400	-0.682377
H180	11.816000	-1.126991	-0.661921
H181	-7.192968	-1.688118	0.453625
H182	-7.235200	0.039622	0.902298
H183	-6.961789	-0.439404	-0.777236
H184	-2.812261	-1.326613	2.408716
C185	0.061117	-2.759145	0.852078
C186	0.698653	-1.474971	1.179223
O187	0.092285	-2.237929	-0.490932
C188	2.185403	-1.577782	1.009805
C189	1.134323	-3.821055	1.035623
C190	2.456483	-3.080190	0.710654
H191	0.144219	-0.547340	1.207378
H192	2.522789	-0.913408	0.208498
H193	3.289359	-3.465327	1.302469
H194	2.661916	-1.228686	1.929216
H195	2.710093	-3.205014	-0.343810
H196	1.125198	-4.157971	2.075973
H197	0.963108	-4.689440	0.394941
H198	-0.938596	-2.969355	1.231839
H199	1.383679	4.341681	-5.150424
H200	8.422900	2.766736	-3.214932
H201	9.279677	0.115756	-4.539021
H202	9.145785	6.101387	0.312879
H203	9.198132	4.771239	-0.866428
H204	9.681318	2.426004	2.796272
H205	2.464472	0.117108	10.270860
H206	2.919269	-6.447114	6.646693
H207	2.352185	-4.835913	7.113224
H208	-1.722412	-5.788603	6.310652

H209	-3.323303	-5.117670	5.918339
H210	-5.844103	-6.203707	-0.118797
H211	-4.876761	-8.373464	-4.350306
H212	-1.034644	-6.141055	-5.802306
H213	3.576768	-0.074884	-7.375057
H214	-0.728612	0.386370	-8.504666
H215	-3.920792	-1.043346	-4.481254
H216	-2.831763	-0.896687	0.681544
H217	-4.728262	-0.506178	-0.414834
H218	-0.736424	9.137224	-3.491852
H219	0.555600	7.974249	-3.094833
H220	-6.411068	9.632300	-0.205818
H221	-5.578349	8.456126	-1.246326
H222	-5.696807	5.710403	2.297018
H223	7.365144	1.771778	-4.227088
H224	11.881402	-0.105978	-2.113016
H225	8.660967	0.822916	-1.619132
H226	7.749000	-0.312756	-2.627336
H227	-2.371609	7.021227	0.098667
H228	-1.624877	-0.197436	-7.099558
H229	-6.412675	2.454056	-5.135148
H230	-4.831014	3.223060	-5.347987
H231	-2.506606	-1.521797	-5.439664
H232	3.006770	4.054792	-4.505569
H233	4.769860	-2.481047	5.642422
H234	3.603184	-1.526175	4.784216
H235	8.177471	4.427998	1.894130
H236	1.573757	-1.330364	-7.910507
H237	-2.154713	7.801060	-1.891851
H238	-7.714810	7.571235	0.604313
H239	-4.784555	-1.921984	3.379939
H240	-6.302982	-1.598519	2.595795
H241	-3.982124	-5.794390	-2.036028
H242	-2.535302	-8.230859	-4.332853
H243	-0.866422	-1.737816	-0.760609
H244	-0.318329	-1.670852	3.592035
H245	1.192631	-2.183175	3.643062
S246	0.396140	-5.420298	-2.407113
H247	0.188471	-4.165749	-1.956934
H248	-1.876954	-5.419433	1.386366

TS attack at C2:

C1	-6.328857	5.541830	1.422878
C2	-5.898596	6.601785	0.421527
O3	-4.776134	6.572051	-0.083115
C4	-7.798101	5.471700	1.850357
C5	-8.080285	4.447239	2.959717
S6	-7.733244	2.683805	2.556814
C7	-6.072936	2.446475	3.291225
N8	-6.800542	7.582849	0.150618
C9	-6.496816	8.682198	-0.735647
C10	-1.286630	6.867207	0.315468
C11	-0.444377	7.148974	-0.921268
O12	0.762143	6.914548	-0.968547
C13	-1.047799	5.456836	0.873911
C14	-1.912654	5.108786	2.100121
C15	-1.751357	3.624908	2.462203
C16	-1.597942	5.994576	3.315356
N17	-1.129127	7.720968	-1.957193
C18	-0.495928	8.082828	-3.210406
C19	2.419594	0.041185	9.178021
C20	1.339092	-0.523641	8.494255
C21	3.448002	0.632193	8.438314
C22	1.284690	-0.519091	7.096962
C23	3.405761	0.647921	7.044698
C24	2.325846	0.066055	6.367940
O25	2.380801	0.066722	4.995693
C26	2.573408	-5.530843	6.232805
C27	3.735508	-4.725616	5.651299
C28	3.236377	-3.436615	5.023372
N29	4.065281	-2.374492	5.023571
O30	2.109073	-3.398561	4.505579
C31	8.977782	1.988066	2.046222
C32	9.089825	2.883032	0.832255
O33	9.576037	2.506969	-0.234719
C34	7.572564	1.889604	2.675931
C35	6.459482	1.372356	1.742476
C36	6.690571	-0.084543	1.314619
C37	5.085593	1.534404	2.409258
N38	8.639720	4.149006	1.031495
C39	8.652925	5.137866	-0.013533
C40	8.415591	2.009555	-4.036513
C41	9.260992	0.775893	-3.698768
C42	8.734768	0.061129	-2.451845
S43	9.741032	-1.364186	-1.871620
C44	11.237691	-0.487569	-1.282061
C45	1.975519	3.701536	-4.456472
C46	1.422036	3.790931	-3.027314
C47	2.324234	3.016123	-2.046491
C48	-0.034846	3.304899	-2.996262
C49	1.976499	3.217656	-0.567103
C50	-5.434804	2.641074	-4.662768
C51	-5.604508	3.333580	-3.303013
C52	-6.429549	2.457035	-2.349902
C53	-4.243243	3.680507	-2.682953
C54	-6.754523	-0.708621	0.238870
N55	-5.312681	-0.710469	0.404789
C56	-4.672087	-1.045836	1.533184
N57	-5.344874	-1.378300	2.661369
N58	-3.342932	-0.997966	1.571776
C59	-2.838166	-0.955019	-4.581661
C60	-2.181713	-1.480607	-3.302217
C61	-2.768871	-0.932558	-2.000972
O62	-3.906663	-0.427403	-1.988312
O63	-2.055277	-1.081866	-0.928846
C64	-0.705842	-0.324276	-7.685712
C65	0.487156	-0.023190	-6.804216
O66	0.436506	0.787703	-5.883804
N67	1.620249	-0.700643	-7.145932
C68	2.923583	-0.426633	-6.570522
C69	3.587297	-1.651559	-5.904683
C70	3.322985	-1.832282	-4.393124
C71	4.071437	-0.780132	-3.560090
C72	1.826790	-1.846073	-4.049597
C73	-0.923254	-6.290976	-4.563066
C74	-2.384764	-6.220129	-4.121536
O75	-2.926291	-5.159332	-3.803115

C76	-0.063816	-5.187329	-3.952360
N77	-3.066624	-7.393965	-4.174221
C78	-4.497208	-7.489330	-3.894489
C79	-4.853556	-7.621748	-2.404648
O80	-5.501509	-8.580216	-1.994925
N81	-4.420693	-6.604129	-1.618696
C82	-4.819591	-6.540775	-0.219659
C83	-4.023699	-5.531581	0.613891
C84	-2.547804	-5.927998	0.746720
C85	-4.693530	-5.393545	1.988292
C86	-2.321690	-5.267043	5.533246
C87	-1.738793	-3.856679	5.358578
C88	-2.535737	-2.946048	4.407227
O89	-3.780581	-3.052244	4.371451
O90	-1.868704	-2.089935	3.715601
O91	0.602645	-1.517816	3.341434
H92	-8.128476	6.444257	2.245972
H93	-8.429394	5.245463	0.982515
H94	-7.521244	4.696285	3.868151
H95	-9.142412	4.482559	3.219243
H96	-6.072022	2.751797	4.340722
H97	-5.860195	1.376156	3.242128
H98	-5.297571	2.985803	2.743925
H99	-6.022344	4.581693	0.996391
H100	-7.227776	8.750093	-1.548672
H101	-1.262271	4.729651	0.080591
H102	0.015314	5.343071	1.116597
H103	-2.960950	5.278531	1.814425
H104	-2.383333	3.349653	3.314375
H105	-2.020626	2.975291	1.622578
H106	-0.713569	3.399783	2.737334
H107	-2.217428	5.715756	4.175408
H108	-1.777177	7.055071	3.112201
H109	-0.548453	5.885449	3.616015
H110	-0.997578	7.619521	1.060566
H111	-0.827303	7.440647	-4.035287
H112	0.525011	-0.981105	9.049399
H113	4.292015	1.088384	8.948255
H114	0.446287	-0.971913	6.574641
H115	4.197104	1.112778	6.464528
H116	1.571167	-0.297831	4.576714
H117	4.238471	-5.300608	4.862393
H118	4.494749	-4.509543	6.411561
H119	1.826892	-5.731249	5.463035
H120	7.272050	2.869014	3.074563
H121	7.638226	1.230779	3.552611
H122	6.463387	1.996095	0.836916
H123	5.882281	-0.427222	0.660138
H124	6.711318	-0.748045	2.188822
H125	7.628346	-0.219639	0.768142
H126	4.889002	2.576711	2.683004
H127	4.282437	1.213539	1.737891
H128	5.008428	0.932798	3.323416
H129	9.330139	1.000807	1.740367
H130	7.640630	5.388978	-0.352630
H131	1.435509	4.844089	-2.712056
H132	-0.474432	3.379419	-1.996765
H133	-0.657692	3.901588	-3.671995
H134	-0.106009	2.262294	-3.327650
H135	2.293029	1.944921	-2.296394
H136	3.363054	3.335335	-2.209043
H137	1.941336	4.284411	-0.321112
H138	1.000027	2.791102	-0.315053
H139	2.719679	2.741875	0.083474
H140	1.906518	2.677849	-4.840233
H141	-6.153842	4.273185	-3.467412
H142	-6.571506	2.942186	-1.377300
H143	-7.421396	2.238316	-2.763312
H144	-5.913146	1.505344	-2.179507
H145	-4.358152	4.266683	-1.764497
H146	-3.629215	4.267492	-3.375531
H147	-3.689985	2.765704	-2.439836
H148	-4.893706	1.694965	-4.545939
H149	-1.103187	-1.289253	-3.303766
H150	-2.305873	-2.571865	-3.246568
H151	-2.579676	0.093663	-4.755467
H152	-0.679361	-1.331581	-8.112543
H153	3.264312	-2.554293	-6.441362
H154	4.671852	-1.582549	-6.057191
H155	3.741479	-2.813585	-4.126406
H156	3.957810	-0.980167	-2.489376
H157	3.690218	0.230761	-3.742540
H158	5.144367	-0.781223	-3.784428
H159	1.665593	-2.073588	-2.990736
H160	1.361889	-0.876706	-4.255648
H161	1.294348	-2.598835	-4.640236
H162	2.782158	0.389245	-5.859905
H163	-0.607662	-4.244403	-4.002812
H164	0.871555	-5.089475	-4.506556
H165	-0.503391	-7.283130	-4.359264
H166	-4.973554	-6.593660	-4.305560
H167	-4.074649	-4.554069	0.109554
H168	-5.743533	-5.094274	1.889329
H169	-4.196585	-4.651546	2.617208
H170	-4.675881	-6.351022	2.523552
H171	-2.061192	-6.029657	-0.227568
H172	-2.453611	-6.886227	1.272475
H173	-4.715145	-7.546342	0.202024
H174	-1.727721	-3.346814	6.332819
H175	-0.697247	-3.898052	5.023128
H176	-2.261322	-5.837149	4.600569
H177	8.796058	2.520606	-4.927155
H178	10.297346	1.089412	-3.528521
H179	10.950737	0.382398	-0.686642
H180	11.796374	-1.191580	-0.660989
H181	-7.199376	-1.693806	0.436622
H182	-7.243913	0.035758	0.878817
H183	-6.967144	-0.449782	-0.798502
H184	-2.845789	-1.331652	2.412640
C185	0.327413	-2.373311	1.332155
C186	0.743881	-1.268828	0.454880
O187	0.161727	-2.048724	-0.606357
C188	2.261159	-1.331285	0.404076
C189	1.375143	-3.446065	1.325507
C190	2.573598	-2.842370	0.539203
H191	0.280628	-0.289206	0.573569
H192	2.654883	-0.887553	-0.513659
H193	3.525310	-3.023208	1.044716
H194	2.653120	-0.766384	1.256906
H195	2.632058	-3.303768	-0.448756

H196	1.628612	-3.710632	2.354967
H197	0.977207	-4.357098	0.867014
H198	-0.702156	-2.523009	1.631763
H199	1.402427	4.337562	-5.141049
H200	8.428214	2.722752	-3.205697
H201	9.279853	0.077129	-4.543704
H202	9.144090	6.058427	0.320357
H203	9.210756	4.723718	-0.853199
H204	9.683107	2.352539	2.805210
H205	2.453047	0.029671	10.262466
H206	2.928990	-6.483915	6.634425
H207	2.081480	-4.983081	7.041607
H208	-1.778565	-5.821956	6.304821
H209	-3.375052	-5.205355	5.815757
H210	-5.890251	-6.295910	-0.157622
H211	-4.898475	-8.370381	-4.396025
H212	-0.916169	-6.171211	-5.655603
H213	3.581719	-0.047908	-7.363908
H214	-0.736491	0.395559	-8.511431
H215	-3.925561	-1.031508	-4.509362
H216	-2.836748	-0.889500	0.683011
H217	-4.735510	-0.504936	-0.433061
H218	-0.707848	9.125764	-3.471426
H219	0.578992	7.955816	-3.078228
H220	-6.479854	9.638755	-0.200102
H221	-5.510115	8.497291	-1.159383
H222	-5.690958	5.693577	2.304321
H223	7.370803	1.739168	-4.229347
H224	11.873997	-0.171329	-2.112141
H225	8.650108	0.768805	-1.622412
H226	7.740524	-0.359470	-2.641391
H227	-2.353510	7.007530	0.110807
H228	-1.616332	-0.202307	-7.100552
H229	-6.404649	2.417358	-5.122389
H230	-4.868583	3.267178	-5.361619
H231	-2.506582	-1.539475	-5.444981
H232	3.022807	4.023749	-4.502058
H233	4.905340	-2.387696	5.580282
H234	3.681682	-1.468394	4.758481
H235	8.222398	4.381452	1.919024
H236	1.580267	-1.318644	-7.942609
H237	-2.132549	7.788205	-1.877408
H238	-7.706311	7.550801	0.589444
H239	-4.811930	-1.922217	3.366411
H240	-6.322838	-1.604937	2.569425
H241	-4.005217	-5.794563	-2.069520
H242	-2.552148	-8.251231	-4.309487
H243	-0.835958	-1.663576	-0.824449
H244	-0.351138	-1.731079	3.626221
H245	1.167979	-2.201501	3.774521
S246	0.333595	-5.527588	-2.177709
H247	0.212559	-4.260774	-1.729474
H248	-1.997248	-5.177549	1.324925

4.6 Mutant *S2*

The following atoms were kept fixed in the geometry optimizations of reactant and transition state structures: 1, 9, 10, 18, 19, 26, 31, 39, 40, 45, 47, 51, 56, 61, 65, 75, 79, 83.

Reactant:

C1	-6.397227	5.667616	1.203139
C2	-5.980071	6.667019	0.134300
O3	-4.884050	6.584089	-0.420145
C4	-7.890672	5.466445	1.481493
C5	-8.187447	4.473638	2.615987
S6	-7.638379	2.735353	2.346314
C7	-6.046137	2.687366	3.248227
N8	-6.864963	7.663983	-0.136114
C9	-6.564834	8.729110	-1.065896
C10	-1.353691	6.977244	0.083025
C11	-0.506766	7.223256	-1.158245
O12	0.705264	7.011383	-1.193080
C13	-1.050283	5.633109	0.757143
C14	-1.950291	5.324571	1.969032
C15	-1.719260	3.885621	2.453205
C16	-1.748053	6.318201	3.123413
N17	-1.190612	7.741515	-2.221930
C18	-0.544649	8.071597	-3.478233
C19	2.323391	0.485661	9.205195
C20	1.272216	-0.109868	8.503466
C21	3.381285	1.051276	8.488531
C22	1.274538	-0.156700	7.108470
C23	3.395822	1.015271	7.095410
C24	2.342977	0.407367	6.396107
O25	2.430942	0.357630	5.037333
C26	2.522503	-5.185918	6.459470
C27	3.644447	-4.606391	5.587232
C28	3.175681	-3.329134	4.908678
N29	3.894281	-2.210481	5.136707
O30	2.169915	-3.345037	4.183197
C31	8.920839	2.211303	2.052673
C32	9.048389	3.067972	0.812682
O33	9.544999	2.660813	-0.238196
C34	7.498941	2.109491	2.644650
C35	6.415925	1.569062	1.689506
C36	6.682444	0.115732	1.270140
C37	5.023927	1.706201	2.323206
N38	8.600557	4.341820	0.968229
C39	8.595629	5.284953	-0.119026
C40	8.399794	2.015683	-4.030618
C41	9.315190	0.838900	-3.672855
C42	8.825605	0.110744	-2.419127
S43	9.905126	-1.252893	-1.823459
C44	11.341231	-0.290057	-1.218129
C45	1.955088	3.660980	-4.552584
C46	0.960987	3.976964	-3.423525
C47	-5.448767	2.558466	-4.770337
C48	-5.580703	3.412953	-3.502054
C49	-6.387966	2.669538	-2.428392
C50	-4.205191	3.828859	-2.961167

C51	-6.786428	-0.622650	0.237592
N52	-5.348812	-0.589561	0.468232
C53	-4.728522	-0.933654	1.613984
N54	-5.417873	-1.307391	2.709502
N55	-3.402462	-0.848425	1.695972
C56	-2.836410	-1.020070	-4.545244
C57	-2.185298	-1.648852	-3.311861
C58	-2.739236	-1.101902	-2.014004
O59	-3.743197	-0.408699	-1.946709
O60	-2.106339	-1.430938	-0.880824
C61	-0.685989	-0.488937	-7.655445
C62	0.476606	-0.242390	-6.715487
O63	0.369915	0.448138	-5.703542
N64	1.643100	-0.824671	-7.110104
C65	2.936223	-0.534555	-6.513363
C66	3.669489	-1.765069	-5.937917
C67	3.385523	-2.115157	-4.459836
C68	3.968344	-1.067581	-3.498630
C69	1.895165	-2.350642	-4.177127
C70	-0.876586	-6.587067	-4.506181
C71	-2.293704	-6.409299	-3.969435
O72	-2.750821	-5.305985	-3.657093
C73	0.118850	-5.620367	-3.875287
N74	-3.042236	-7.539513	-3.927744
C75	-4.470336	-7.533962	-3.638959
C76	-4.861839	-7.603776	-2.154000
O77	-5.629210	-8.474208	-1.755686
N78	-4.335405	-6.629883	-1.366496
C79	-4.821928	-6.458018	-0.002093
C80	-3.881951	-5.688978	0.928169
C81	-2.559217	-6.432786	1.149336
C82	-4.620447	-5.435817	2.250975
C83	-2.368891	-4.970362	5.718688
C84	-1.812203	-3.558801	5.484229
C85	-2.606925	-2.717224	4.470844
O86	-3.854197	-2.863280	4.420188
O87	-1.943573	-1.896240	3.748219
O88	0.656275	-1.062754	3.747254
H89	-8.362335	6.414791	1.781252
H90	-8.397884	5.134726	0.568096
H91	-7.747908	4.816880	3.558742
H92	-9.268910	4.416975	2.770013
H93	-6.176438	3.043435	4.273217
H94	-5.741652	1.638760	3.281609
H95	-5.268556	3.266210	2.746416
H96	-5.946750	4.715739	0.909734
H97	-7.404592	8.896825	-1.747569
H98	-1.170808	4.831823	0.018779
H99	0.003877	5.612133	1.057237
H100	-2.992839	5.407176	1.626648

H101	-2.378656	3.635400	3.292253	H161	-2.742376	-7.413961	1.603755
H102	-1.899531	3.159484	1.654031	H162	-5.011817	-7.459188	0.396710
H103	-0.686735	3.746909	2.794900	H163	-1.829615	-2.999757	6.430682
H104	-2.389186	6.065880	3.975737	H164	-0.765233	-3.591134	5.165856
H105	-1.980607	7.347599	2.832060	H165	-2.287554	-5.583295	4.814975
H106	-0.709534	6.297966	3.476221	H166	8.752505	2.536357	-4.927251
H107	-1.128724	7.801641	0.772583	H167	10.330413	1.214962	-3.501851
H108	-0.734851	7.316306	-4.251027	H168	10.996891	0.571339	-0.640618
H109	0.439125	-0.550025	9.045025	H169	11.923604	-0.954681	-0.575799
H110	4.203215	1.526624	9.017432	H170	-7.205024	-1.627907	0.379095
H111	0.465104	-0.631138	6.562065	H171	-7.318865	0.082729	0.885238
H112	4.209954	1.457264	6.528802	H172	-6.961714	-0.326343	-0.796510
H113	1.618433	-0.061395	4.620643	H173	-2.891748	-1.207021	2.539073
H114	3.904031	-5.319269	4.796435	C174	-0.080106	-3.366346	1.001997
H115	4.550485	-4.432081	6.177802	C175	0.709621	-2.183564	0.670863
H116	1.628188	-5.354117	5.855787	O176	0.024840	-2.928970	-0.407283
H117	7.179633	3.092887	3.018655	C177	2.180982	-2.506481	0.777768
H118	7.546232	1.465930	3.533558	C178	0.855679	-4.504801	1.346531
H119	6.429021	2.190552	0.782151	C179	2.232472	-4.054552	0.793962
H120	5.889930	-0.244291	0.606043	H180	0.334113	-1.177847	0.821521
H121	6.702610	-0.544023	2.146815	H181	2.775699	-2.060736	-0.025892
H122	7.631318	-0.001561	0.738714	H182	3.054691	-4.428497	1.408127
H123	4.818804	2.740501	2.622196	H183	2.529711	-2.091643	1.728816
H124	4.242576	1.409426	1.616882	H184	2.378331	-4.437799	-0.218893
H125	4.920830	1.078514	3.216228	H185	0.892388	-4.573952	2.439100
H126	9.296990	1.220897	1.788214	H186	0.526131	-5.471638	0.954630
H127	7.590395	5.433083	-0.533324	H187	-1.077488	-3.291903	1.424963
H128	1.066560	5.020938	-3.111571	H188	1.835552	4.364686	-5.384355
H129	1.795535	2.652198	-4.945066	H189	8.367070	2.739409	-3.209561
H130	-6.131480	4.328137	-3.766788	H190	9.379585	0.131358	-4.508267
H131	-6.519481	3.281131	-1.529235	H191	8.982564	6.256347	0.205992
H132	-7.383019	2.391016	-2.794946	H192	9.239663	4.889574	-0.904447
H133	-5.867300	1.750982	-2.131593	H193	9.598120	2.612617	2.818519
H134	-4.300741	4.493780	-2.096295	H194	2.313992	0.515503	10.290276
H135	-3.621057	4.354189	-3.725608	H195	2.829097	-6.137374	6.903182
H136	-3.630147	2.947703	-2.652036	H196	2.260900	-4.500785	7.271846
H137	-4.923861	1.621493	-4.548910	H197	-1.822920	-5.479023	6.519964
H138	-1.099317	-1.497997	-3.321432	H198	-3.426098	-4.918307	5.988936
H139	-2.344665	-2.737083	-3.296147	H199	-5.798089	-5.949867	-0.026940
H140	-2.514384	0.016248	-4.671368	H200	-4.931068	-8.397151	-4.120637
H141	-0.658317	-1.485592	-8.107181	H201	-0.922581	-6.389901	-5.586691
H142	3.440611	-2.633938	-6.570619	H202	3.569151	-0.066905	-7.278576
H143	4.747982	-1.592665	-6.044161	H203	-0.658996	0.248805	-8.465402
H144	3.914685	-3.060073	-4.270066	H204	-3.924896	-1.038226	-4.458817
H145	3.851836	-1.390221	-2.458615	H205	-2.876492	-0.739984	0.837281
H146	3.462055	-0.100579	-3.588758	H206	-4.750441	-0.375116	-0.332215
H147	5.037699	-0.910393	-3.679693	H207	-0.890480	9.043451	-3.845252
H148	1.747116	-2.712392	-3.153457	H208	0.529157	8.114805	-3.293915
H149	1.322006	-1.425702	-4.294143	H209	-6.338475	9.670795	-0.550770
H150	1.474641	-3.093680	-4.863265	H210	-5.689877	8.428409	-1.641949
H151	2.758905	0.218087	-5.743101	H211	-5.884922	5.974065	2.126415
H152	-0.302144	-4.615523	-3.886590	H212	7.373399	1.681152	-4.222166
H153	1.051571	-5.615328	-4.442869	H213	11.976598	0.048745	-2.039995
H154	-0.543563	-7.625139	-4.393437	H214	8.701676	0.821212	-1.597009
H155	-4.890776	-6.619643	-4.070871	H215	7.854924	-0.363168	-2.604953
H156	-3.661656	-4.712329	0.467993	H216	-2.422186	7.043501	-0.152569
H157	-5.588593	-4.952638	2.074379	H217	-1.622469	-0.361115	-7.115153
H158	-4.060906	-4.788897	2.928919	H218	-6.430748	2.300413	-5.183780
H159	-4.817864	-6.383103	2.767651	H219	-4.884245	3.082620	-5.549585
H160	-2.022671	-6.595789	0.209271	H220	-2.551147	-1.580670	-5.438733

H221	2.988085	3.734120	-4.196092
H222	4.613592	-2.212043	5.842574
H223	3.510215	-1.302830	4.868794
H224	8.144225	4.590874	1.831965
H225	1.637024	-1.346454	-7.974241
H226	-2.196537	7.787617	-2.160060
H227	-7.730958	7.700031	0.377030
H228	-4.880756	-1.843366	3.430763
H229	-6.398527	-1.515078	2.607106
H230	-3.826100	-5.879304	-1.822459
H231	-2.592696	-8.428331	-4.086209
H232	-1.311178	-2.033348	-0.937912
H233	-0.299649	-1.301099	3.835004
H234	1.142076	-1.908386	3.837425
S235	0.493759	-6.071142	-2.122784
H236	0.360972	-4.834497	-1.600251
H237	-1.904974	-5.873196	1.825361
C238	1.137384	3.083601	-2.213176
C239	1.526158	3.621025	-0.978626
H240	1.666880	4.694740	-0.890221
C241	1.695534	2.797801	0.136798
H242	1.980601	3.235544	1.089589
C243	1.486806	1.421153	0.036787
H244	1.614329	0.792297	0.913811
C245	0.928315	1.699451	-2.305823
H246	0.624594	1.265661	-3.255601
C247	1.104635	0.876989	-1.192542
H248	-0.059905	3.865458	-3.813565
H249	0.933941	-0.191162	-1.290708

TS attack at C1:

C1	-6.316277	5.647773	1.346586
C2	-5.883638	6.685771	0.323243
O3	-4.772941	6.625560	-0.204600
C4	-7.786533	5.583071	1.770300
C5	-8.071516	4.570741	2.890665
S6	-7.722433	2.802655	2.508518
C7	-6.051929	2.577240	3.224028
N8	-6.768570	7.682976	0.056816
C9	-6.452653	8.766884	-0.844738
C10	-1.260263	6.922378	0.242441
C11	-0.404616	7.223249	-0.981115
O12	0.817285	7.075920	-0.995408
C13	-0.931238	5.567500	0.883504
C14	-1.835539	5.209322	2.078272
C15	-1.580135	3.763471	2.528454
C16	-1.665559	6.177248	3.259482
N17	-1.096092	7.720879	-2.050181
C18	-0.448587	8.095318	-3.293109
C19	2.363578	0.159664	9.187217
C20	1.268506	-0.399918	8.522406
C21	3.409429	0.692349	8.427885
C22	1.215645	-0.446184	7.125900
C23	3.369301	0.656589	7.034654
C24	2.273675	0.081404	6.376628
O25	2.328439	0.033080	5.007021
C26	2.483468	-5.443524	6.300112
C27	3.640736	-4.823703	5.508133
C28	3.188131	-3.529420	4.858135
N29	3.945922	-2.432144	5.052448
O30	2.151043	-3.513997	4.176058
C31	8.960014	1.981686	2.057701
C32	9.089452	2.865912	0.837222
O33	9.556632	2.471160	-0.231378
C34	7.544301	1.903283	2.667551
C35	6.433467	1.426316	1.710431
C36	6.641314	-0.024743	1.252508
C37	5.053473	1.601795	2.360313
N38	8.673320	4.145069	1.030274
C39	8.666500	5.112468	-0.035481
C40	8.417861	1.944700	-4.026778
C41	9.293093	0.731122	-3.692101
C42	8.777448	-0.006010	-2.453969
S43	9.816068	-1.409545	-1.877542
C44	11.283700	-0.498832	-1.267230
C45	1.992610	3.682294	-4.485684
C46	1.044288	4.121510	-3.358143
C47	-5.425059	2.677429	-4.705817
C48	-5.544813	3.445400	-3.382115
C49	-5.991986	2.508048	-2.251255
C50	-4.228573	4.151243	-3.025463
C51	-6.786896	-0.611078	0.225643
N52	-5.347096	-0.695876	0.384078
C53	-4.712108	-1.028283	1.516680
N54	-5.384511	-1.276614	2.666448
N55	-3.382952	-1.064375	1.532478
C56	-2.856872	-0.937723	-4.578972
C57	-2.210066	-1.593430	-3.352164
C58	-2.764583	-1.109573	-2.012980
O59	-3.878046	-0.557238	-1.969764
O60	-2.059437	-1.340579	-0.946949
C61	-0.709569	-0.355689	-7.682209
C62	0.478258	-0.065682	-6.788231
O63	0.426941	0.750963	-5.871504
N64	1.606614	-0.756943	-7.116407
C65	2.915288	-0.474745	-6.553894
C66	3.584091	-1.677649	-5.856724
C67	3.282987	-1.864757	-4.352398
C68	3.931560	-0.764655	-3.497684
C69	1.782362	-1.979476	-4.050304
C70	-0.990464	-6.388970	-4.645859
C71	-2.415371	-6.271249	-4.111383
O72	-2.899634	-5.194317	-3.755629
C73	-0.039544	-5.365308	-4.033078
N74	-3.141483	-7.417969	-4.128825
C75	-4.569147	-7.451483	-3.830045
C76	-4.934027	-7.573173	-2.341379
O77	-5.638748	-8.495836	-1.943514
N78	-4.459498	-6.576476	-1.551400
C79	-4.896100	-6.462532	-0.166284
C80	-3.955664	-5.646391	0.726150
C81	-2.584639	-6.317374	0.877740
C82	-4.636269	-5.432923	2.085478
C83	-2.407111	-5.148898	5.581510
C84	-1.800610	-3.750658	5.398801
C85	-2.572328	-2.857909	4.411606
O86	-3.821325	-2.921566	4.397842

O87	-1.882315	-2.075408	3.661486
O88	0.603308	-1.463217	3.355999
H89	-8.118998	6.559949	2.153569
H90	-8.414914	5.346934	0.903191
H91	-7.515539	4.830029	3.798133
H92	-9.134459	4.608316	3.146316
H93	-6.038969	2.889684	4.271328
H94	-5.835232	1.507278	3.178836
H95	-5.285735	3.115923	2.663464
H96	-6.006911	4.680263	0.939897
H97	-7.245907	8.902456	-1.587127
H98	-1.029741	4.784387	0.123000
H99	0.121274	5.560100	1.189857
H100	-2.876243	5.279728	1.728138
H101	-2.246841	3.477430	3.350203
H102	-1.732222	3.055543	1.707504
H103	-0.549369	3.639988	2.881946
H104	-2.308980	5.889559	4.098883
H105	-1.917787	7.208101	2.991111
H106	-0.629910	6.170968	3.621650
H107	-1.064313	7.732876	0.956545
H108	-0.603027	7.347048	-4.080387
H109	0.440797	-0.812386	9.092572
H110	4.265733	1.143051	8.922057
H111	0.363461	-0.890859	6.618888
H112	4.174266	1.076845	6.439317
H113	1.518597	-0.345267	4.591256
H114	3.949032	-5.503462	4.704973
H115	4.515597	-4.660271	6.146620
H116	1.622441	-5.598648	5.647507
H117	7.260165	2.883832	3.075561
H118	7.584399	1.231343	3.535773
H119	6.461077	2.069009	0.818634
H120	5.836142	-0.334570	0.578219
H121	6.635704	-0.708643	2.110917
H122	7.584716	-0.165405	0.717307
H123	4.892283	2.635649	2.686235
H124	4.253182	1.352394	1.656749
H125	4.936699	0.957305	3.240445
H126	9.305490	0.988884	1.762229
H127	7.653446	5.307593	-0.408737
H128	1.234628	5.165349	-3.087755
H129	1.745768	2.676528	-4.837762
H130	-6.317240	4.219323	-3.509764
H131	-6.095369	3.046329	-1.302181
H132	-6.960796	2.047562	-2.480191
H133	-5.262169	1.702883	-2.107935
H134	-4.329025	4.751729	-2.114996
H135	-3.906087	4.817539	-3.834479
H136	-3.428481	3.419477	-2.860840
H137	-4.670369	1.886359	-4.625741
H138	-1.123808	-1.452689	-3.354457
H139	-2.383045	-2.678888	-3.375145
H140	-2.541973	0.105141	-4.679934
H141	-0.697815	-1.370419	-8.091687
H142	3.307291	-2.592259	-6.399641
H143	4.670408	-1.575368	-5.975371
H144	3.755734	-2.816638	-4.070144
H145	3.800055	-0.975329	-2.431014
H146	3.481986	0.216567	-3.683903
H147	5.007715	-0.688605	-3.692106
H148	1.615606	-2.234469	-2.998116
H149	1.265118	-1.036267	-4.252204
H150	1.312667	-2.756852	-4.662319
H151	2.780416	0.360207	-5.864035
H152	-0.515166	-4.385060	-4.033487
H153	0.880623	-5.310669	-4.618191
H154	-0.609365	-7.408744	-4.518247
H155	-5.011748	-6.534420	-4.232574
H156	-3.809758	-4.659235	0.260256
H157	-5.620693	-4.966297	1.965572
H158	-4.053777	-4.787865	2.746639
H159	-4.788035	-6.392076	2.596123
H160	-2.089932	-6.453976	-0.088842
H161	-2.688129	-7.305061	1.343563
H162	-5.002869	-7.479690	0.223364
H163	-1.805632	-3.224597	6.364087
H164	-0.753780	-3.811758	5.082984
H165	-2.342623	-5.731424	4.656748
H166	8.790109	2.471704	-4.911648
H167	10.319756	1.070261	-3.512496
H168	10.968599	0.363067	-0.674194
H169	11.850808	-1.190272	-0.639714
H170	-7.288678	-1.566042	0.435811
H171	-7.227837	0.167212	0.860169
H172	-6.989201	-0.349228	-0.813059
H173	-2.880244	-1.380894	2.379223
C174	-0.119886	-3.031449	0.939484
C175	0.639130	-1.789417	1.144223
O176	-0.056782	-2.625857	-0.447256
C177	2.107698	-2.048096	0.985276
C178	0.855226	-4.168409	1.201980
C179	2.229084	-3.585427	0.786467
H180	0.169717	-0.817739	1.103047
H181	2.510241	-1.466782	0.150500
H182	3.040775	-4.005919	1.382963
H183	2.616871	-1.697032	1.885901
H184	2.431797	-3.807008	-0.263201
H185	0.843806	-4.399394	2.271251
H186	0.586453	-5.074967	0.654505
H187	-1.129399	-3.112641	1.339630
H188	1.917194	4.361283	-5.342844
H189	8.407048	2.651926	-3.190871
H190	9.335762	0.039035	-4.541668
H191	9.102439	6.061066	0.294936
H192	9.266372	4.710740	-0.851930
H193	9.658747	2.344753	2.823225
H194	2.395182	0.188135	10.271378
H195	2.778825	-6.406836	6.725114
H196	2.174453	-4.790862	7.122411
H197	-1.884992	-5.704696	6.367098
H198	-3.463073	-5.066396	5.848348
H199	-5.900867	-6.014622	-0.135142
H200	-5.013324	-8.311626	-4.331890
H201	-1.046071	-6.211316	-5.729497
H202	3.569682	-0.120072	-7.361339
H203	-0.709347	0.349652	-8.520970
H204	-3.945012	-0.958696	-4.488807
H205	-2.881116	-1.013398	0.637896
H206	-4.759321	-0.562554	-0.459963

H207	-0.824153	9.060663	-3.648287
H208	0.620728	8.173841	-3.094538
H209	-6.309612	9.713086	-0.308858
H210	-5.524907	8.509882	-1.355247
H211	-5.681384	5.817808	2.227095
H212	7.381548	1.648543	-4.227629
H213	11.923155	-0.165729	-2.088276
H214	8.669194	0.692779	-1.619793
H215	7.795387	-0.450105	-2.653004
H216	-2.326793	6.966871	-0.004631
H217	-1.627508	-0.203030	-7.116422
H218	-6.374316	2.203009	-4.980151
H219	-5.131327	3.339852	-5.528125
H220	-2.571266	-1.472530	-5.489348
H221	3.033366	3.687560	-4.143686
H222	4.699595	-2.451968	5.721261
H223	3.561314	-1.521192	4.802378
H224	8.248188	4.387568	1.911542
H225	1.564181	-1.377475	-7.911177
H226	-2.104063	7.713754	-2.005084
H227	-7.659006	7.687236	0.527219
H228	-4.865011	-1.818233	3.383180
H229	-6.374673	-1.452855	2.597752
H230	-3.991717	-5.796581	-2.003797
H231	-2.672025	-8.292107	-4.310178
H232	-0.951984	-2.053621	-0.755112
H233	-0.356675	-1.718244	3.577096
H234	1.158196	-2.224102	3.654747
S235	0.407932	-5.788542	-2.289592
H236	0.127142	-4.591902	-1.732818
H237	-1.926445	-5.719194	1.516946
C238	1.177252	3.261747	-2.119971
C239	1.739180	3.778058	-0.945012
H240	2.026909	4.824876	-0.910107
C241	1.881847	2.976054	0.188659
H242	2.308921	3.395814	1.095358
C243	1.459549	1.646262	0.166734
H244	1.560306	1.037526	1.061508
C245	0.757218	1.923620	-2.132221
H246	0.316183	1.511380	-3.036710
C247	0.893717	1.121739	-0.999559
H248	0.011571	4.072574	-3.727722
H249	0.535482	0.097487	-1.031002

TS attack at C2:

C1	-6.307911	5.638989	1.266003
C2	-5.875428	6.663792	0.228754
O3	-4.764975	6.597157	-0.298005
C4	-7.774043	5.590838	1.705421
C5	-8.054404	4.594778	2.841460
S6	-7.713381	2.820422	2.481466
C7	-6.044218	2.597051	3.200809
N8	-6.759999	7.659003	-0.047874
C9	-6.442542	8.734682	-0.958388
C10	-1.251326	6.896530	0.145036
C11	-0.387461	7.198225	-1.073738
O12	0.840274	7.120568	-1.061913
C13	-0.766914	5.690195	0.956038
C14	-1.668549	5.348046	2.157803
C15	-1.224117	4.026691	2.802021
C16	-1.711702	6.468564	3.208540
N17	-1.086881	7.626316	-2.168790
C18	-0.440786	8.030846	-3.403353
C19	2.371500	0.225900	9.159102
C20	1.298171	-0.371431	8.491882
C21	3.394577	0.804039	8.401692
C22	1.245491	-0.411552	7.094911
C23	3.354194	0.775038	7.008297
C24	2.281344	0.160845	6.348493
O25	2.339710	0.121379	4.977559
C26	2.483747	-5.407891	6.331868
C27	3.689776	-4.594026	5.865737
C28	3.235739	-3.337908	5.145182
N29	4.077199	-2.285850	5.113689
O30	2.129144	-3.317155	4.586308
C31	8.965086	1.964956	2.006268
C32	9.096573	2.835644	0.776275
O33	9.562178	2.428074	-0.288157
C34	7.545944	1.894742	2.611414
C35	6.435413	1.412857	1.655414
C36	6.640524	-0.042638	1.210557
C37	5.054421	1.596896	2.302110
N38	8.680931	4.116943	0.955420
C39	8.673394	5.073530	-0.120009
C40	8.418877	1.863617	-4.077117
C41	9.292285	0.652156	-3.730275
C42	8.776825	-0.070027	-2.483273
S43	9.812282	-1.470107	-1.893138
C44	11.282684	-0.557016	-1.293100
C45	1.995105	3.602836	-4.550362
C46	1.150940	4.241549	-3.438316
C47	-5.423732	2.603326	-4.754943
C48	-5.541071	3.371500	-3.431183
C49	-5.989482	2.435454	-2.299651
C50	-4.223729	4.075994	-3.075743
C51	-6.785677	-0.630977	0.212208
N52	-5.347989	-0.738912	0.372722
C53	-4.719169	-1.041419	1.515976
N54	-5.396758	-1.240757	2.673089
N55	-3.390669	-1.099393	1.534827
C56	-2.859165	-1.012912	-4.591201
C57	-2.236810	-1.687998	-3.363009
C58	-2.793336	-1.198888	-2.026491
O59	-3.905794	-0.642783	-1.987928
O60	-2.091111	-1.432869	-0.960411
C61	-0.713315	-0.466233	-7.701866
C62	0.464908	-0.185814	-6.793984
O63	0.388435	0.597215	-5.850058
N64	1.603549	-0.850747	-7.140831
C65	2.912161	-0.576979	-6.574700
C66	3.579261	-1.787379	-5.889259
C67	3.277339	-1.987607	-4.387038
C68	3.909995	-0.881613	-3.527576
C69	1.777530	-2.126117	-4.088837
C70	-0.932535	-6.567891	-4.447020
C71	-2.380333	-6.400924	-3.991728
O72	-2.847321	-5.304980	-3.672676

C73	0.012397	-5.552907	-3.809160
N74	-3.141160	-7.524727	-4.034479
C75	-4.577610	-7.516544	-3.771705
C76	-4.970259	-7.604393	-2.287709
O77	-5.693613	-8.508304	-1.879972
N78	-4.481034	-6.608824	-1.507588
C79	-4.901131	-6.488224	-0.118493
C80	-4.028941	-5.538158	0.708614
C81	-2.601361	-6.073726	0.879050
C82	-4.704581	-5.297162	2.065370
C83	-2.407001	-5.115908	5.613341
C84	-1.879396	-3.677743	5.513834
C85	-2.647127	-2.804235	4.506659
O86	-3.894699	-2.877851	4.480120
O87	-1.953256	-2.027043	3.753215
O88	0.553939	-1.569941	3.337591
H89	-8.095107	6.574986	2.079546
H90	-8.413207	5.348131	0.847962
H91	-7.490605	4.864664	3.740969
H92	-9.115261	4.639247	3.104583
H93	-6.033759	2.910698	4.247779
H94	-5.826236	1.527208	3.156880
H95	-5.277643	3.136310	2.641315
H96	-6.011043	4.664595	0.866315
H97	-7.192630	8.816228	-1.752451
H98	-0.716318	4.817608	0.296337
H99	0.260157	5.873112	1.294247
H100	-2.689373	5.210817	1.769737
H101	-1.892040	3.737146	3.621423
H102	-1.208875	3.211155	2.072235
H103	-0.212772	4.116368	3.217697
H104	-2.340981	6.184074	4.059774
H105	-2.111877	7.402922	2.802435
H106	-0.707156	6.677069	3.597655
H107	-1.217362	7.802568	0.765607
H108	-0.563639	7.283120	-4.196511
H109	0.487620	-0.819119	9.060049
H110	4.233024	1.285360	8.897565
H111	0.411077	-0.887355	6.586817
H112	4.141329	1.229815	6.414541
H113	1.541637	-0.274174	4.567504
H114	4.300919	-5.178834	5.165295
H115	4.345717	-4.330958	6.703720
H116	1.847595	-5.670940	5.485818
H117	7.263396	2.879378	3.010487
H118	7.581405	1.229847	3.485315
H119	6.466731	2.048069	0.758465
H120	5.834354	-0.357602	0.539879
H121	6.633896	-0.718511	2.075378
H122	7.583746	-0.189868	0.676773
H123	4.894345	2.633717	2.618842
H124	4.251491	1.340707	1.603373
H125	4.938926	0.960660	3.188473
H126	9.310527	0.968703	1.722614
H127	7.658255	5.277185	-0.482648
H128	1.533627	5.236981	-3.190874
H129	1.590470	2.635671	-4.862696
H130	-6.312413	4.146566	-3.558607
H131	-6.089721	2.974392	-1.350558
H132	-6.960303	1.978485	-2.527165
H133	-5.262778	1.627494	-2.156923
H134	-4.324253	4.679366	-2.167297
H135	-3.900083	4.739765	-3.886429
H136	-3.424046	3.344547	-2.907989
H137	-4.668322	1.812889	-4.676776
H138	-1.147722	-1.572016	-3.354817
H139	-2.433027	-2.769225	-3.393345
H140	-2.495990	0.012701	-4.705463
H141	-0.670881	-1.455568	-8.167797
H142	3.302091	-2.696253	-6.441314
H143	4.665749	-1.684239	-6.005895
H144	3.764603	-2.933535	-4.109191
H145	3.802165	-1.108478	-2.461776
H146	3.432787	0.089826	-3.695597
H147	4.981013	-0.775520	-3.735937
H148	1.608555	-2.391869	-3.039693
H149	1.247016	-1.188924	-4.283858
H150	1.320708	-2.905821	-4.708280
H151	2.779124	0.252143	-5.876957
H152	-0.440413	-4.562727	-3.851706
H153	0.959334	-5.530423	-4.352093
H154	-0.586157	-7.593762	-4.276255
H155	-4.986514	-6.595392	-4.199335
H156	-3.975092	-4.573670	0.180194
H157	-5.719795	-4.904410	1.938147
H158	-4.152033	-4.581828	2.678419
H159	-4.783570	-6.233458	2.631728
H160	-2.107736	-6.238309	-0.083401
H161	-2.610094	-7.028263	1.419615
H162	-4.893549	-7.491477	0.321204
H163	-1.979513	-3.188643	6.492807
H164	-0.813524	-3.667424	5.263119
H165	-2.256659	-5.658613	4.674123
H166	8.790816	2.380092	-4.968281
H167	10.319841	0.991309	-3.555846
H168	10.970245	0.311543	-0.708430
H169	11.848547	-1.243605	-0.659166
H170	-7.305941	-1.565747	0.463569
H171	-7.210353	0.182637	0.812686
H172	-6.983633	-0.411071	-0.837079
H173	-2.899121	-1.368985	2.402646
C174	0.142357	-2.795945	1.460033
C175	0.661003	-1.842333	0.477835
O176	-0.024872	-2.654350	-0.498189
C177	2.162481	-2.064498	0.423906
C178	1.081714	-3.946827	1.606797
C179	2.329700	-3.568551	0.759448
H180	0.295354	-0.820884	0.518305
H181	2.579031	-1.787699	-0.547331
H182	3.258645	-3.765911	1.299654
H183	2.626869	-1.432979	1.188419
H184	2.348313	-4.162338	-0.156966
H185	1.314865	-4.075313	2.668432
H186	0.599076	-4.875544	1.284362
H187	-0.883660	-2.786854	1.807147
H188	2.027966	4.249679	-5.435028
H189	8.410549	2.580109	-3.249118
H190	9.332382	-0.049343	-4.572197
H191	9.124997	6.020245	0.194670
H192	9.258629	4.655985	-0.939128

H193	9.660742	2.336184	2.770493
H194	2.403320	0.249240	10.243372
H195	2.809297	-6.328692	6.824321
H196	1.876567	-4.838518	7.040826
H197	-1.895342	-5.668938	6.407536
H198	-3.478988	-5.106367	5.823409
H199	-5.946411	-6.147756	-0.081464
H200	-5.031827	-8.373428	-4.269920
H201	-0.925135	-6.414985	-5.535749
H202	3.566237	-0.216037	-7.379358
H203	-0.736582	0.284629	-8.499712
H204	-3.946585	-0.983302	-4.494013
H205	-2.886354	-1.078675	0.639913
H206	-4.760097	-0.624436	-0.475504
H207	-0.842743	8.986411	-3.757013
H208	0.623323	8.142112	-3.192723
H209	-6.378234	9.697803	-0.438214
H210	-5.474266	8.509811	-1.404661
H211	-5.662244	5.812376	2.137899
H212	7.381735	1.567173	-4.273276
H213	11.922292	-0.233799	-2.117944
H214	8.672209	0.638373	-1.656767
H215	7.793117	-0.513392	-2.675746
H216	-2.300800	6.759798	-0.139915
H217	-1.636283	-0.374526	-7.131020
H218	-6.373402	2.128471	-5.027304
H219	-5.132214	3.265967	-5.577970
H220	-2.605500	-1.568961	-5.498616
H221	3.026297	3.448980	-4.212623
H222	4.890907	-2.270037	5.708417
H223	3.705423	-1.394158	4.790992
H224	8.260484	4.370368	1.835859
H225	1.565308	-1.448596	-7.952913
H226	-2.094191	7.576658	-2.134790
H227	-7.653567	7.663950	0.416500
H228	-4.892358	-1.772863	3.407225
H229	-6.389933	-1.400820	2.607288
H230	-3.991445	-5.843554	-1.961641
H231	-2.688408	-8.415786	-4.171337
H232	-0.951100	-2.159975	-0.780371
H233	-0.391598	-1.724512	3.657548
H234	1.127864	-2.205125	3.823901
S235	0.370029	-5.945512	-2.036224
H236	0.117713	-4.720056	-1.531345
H237	-1.992623	-5.370801	1.458407
C238	1.119676	3.395627	-2.185703
C239	1.728993	3.826713	-1.001386
H240	2.183618	4.812305	-0.966373
C241	1.706609	3.026397	0.143086
H242	2.171319	3.382433	1.058564
C243	1.058380	1.791051	0.123826
H244	1.024976	1.185700	1.026086
C245	0.485241	2.145439	-2.199060
H246	0.029346	1.788634	-3.119199
C247	0.442902	1.354334	-1.053702
H248	0.126065	4.383412	-3.806512
H249	-0.094756	0.412088	-1.085815

4.7 Mutant *S3*

The following atoms were kept fixed in the geometry optimizations of reactant and transition state structures: 1, 6, 7, 15, 16, 23, 28, 36, 37, 42, 44, 48, 53, 58, 62, 72, 76, 80.

Reactant:

C1	-5.292504	6.821363	1.827821
C2	-4.773852	7.715610	0.702270
O3	-3.832627	7.394619	-0.020768
C4	-5.098490	5.339134	1.519701
N5	-5.413656	8.914476	0.593388
C6	-5.003727	9.941923	-0.346591
C7	-0.167224	7.189265	0.412476
C8	0.642780	7.314300	-0.875597
O9	1.802859	6.920305	-0.984317
C10	0.388959	6.127911	1.363653
C11	-0.389812	5.965752	2.682777
C12	0.156558	4.767168	3.473255
C13	-0.369908	7.233943	3.550045
N14	-0.007396	7.961027	-1.889314
C15	0.623244	8.242836	-3.165213
C16	2.731222	-0.221113	9.105053
C17	1.541552	-0.572020	8.462070
C18	3.832665	0.156252	8.332490
C19	1.447158	-0.561474	7.069652
C20	3.752475	0.174790	6.941262
C21	2.558722	-0.187464	6.300514
O22	2.548245	-0.196193	4.937835
C23	1.672869	-5.719846	6.208627
C24	2.835162	-5.317944	5.291001
C25	2.572428	-3.953018	4.675049
N26	3.504024	-2.997416	4.871096
O27	1.535657	-3.755377	4.023735
C28	9.098964	0.486097	1.580796
C29	9.294902	1.343403	0.349823
O30	9.582944	0.881113	-0.754494
C31	7.730520	0.645706	2.277375
C32	6.497869	0.349933	1.399467
C33	6.430879	-1.122152	0.966549
C34	5.209104	0.766118	2.123252
N35	9.152164	2.676591	0.575211
C36	9.241498	3.644893	-0.485858
C37	8.188341	0.621814	-4.458122
C38	8.987387	-0.663230	-4.212069
C39	8.454631	-1.434462	-3.002273
S40	9.392288	-2.952876	-2.560532
C41	10.950176	-2.207739	-1.949403
C42	2.160394	3.480918	-4.513459
C43	1.302166	3.916486	-3.315381
C44	-5.316910	3.815259	-4.272305
C45	-5.342658	4.514654	-2.910980
C46	-6.309964	3.787214	-1.967846
C47	-3.931707	4.604442	-2.312991
C48	-6.939891	0.760653	0.730044
N49	-5.498949	0.680315	0.938957
C50	-4.910440	0.052017	1.989424
N51	-5.606817	-0.342223	3.061448
N52	-3.590755	-0.117111	1.983598
C53	-3.432106	-0.200590	-4.310547
C54	-2.861573	-0.966387	-3.116097
C55	-3.256287	-0.364897	-1.783228
O56	-4.124173	0.486438	-1.652138
O57	-2.636175	-0.828210	-0.691354
C58	-1.409008	0.028444	-7.540775
C59	-0.177918	-0.020123	-6.657995
O60	-0.105927	0.592928	-5.594199
N61	0.844601	-0.760264	-7.170323
C62	2.198471	-0.747945	-6.640679
C63	2.738367	-2.133919	-6.231119
C64	2.488200	-2.567275	-4.769295
C65	3.314494	-1.738677	-3.773106
C66	1.000806	-2.556004	-4.390492
C67	-2.567993	-6.064012	-4.510456
C68	-3.892543	-5.628242	-3.891036
O69	-4.103853	-4.468361	-3.524763
C70	-1.371319	-5.321293	-3.928050
N71	-4.848912	-6.588323	-3.838022
C72	-6.230152	-6.314341	-3.465059
C73	-6.547994	-6.360617	-1.961674
O74	-7.458303	-7.067828	-1.541864
N75	-5.791454	-5.544062	-1.183029
C76	-6.151024	-5.328588	0.213269
C77	-5.013729	-4.802236	1.092243
C78	-3.867811	-5.814311	1.212866
C79	-5.594083	-4.432554	2.464785
C80	-3.121270	-4.550497	5.796548
C81	-2.341396	-3.245640	5.583571
C82	-3.021452	-2.252109	4.628226
O83	-4.274772	-2.155237	4.664389
O84	-2.262703	-1.566564	3.859760
O85	0.448855	-1.218885	3.759814
H86	-5.719829	5.038488	0.675021
H87	-4.054818	5.150590	1.266802
H88	-4.728586	7.081837	2.733559
H89	-5.844286	10.256392	-0.973669
H90	0.401641	5.163851	0.842657
H91	1.437980	6.363624	1.581651
H92	-1.439131	5.747847	2.430764
H93	-0.420260	4.595778	4.388233
H94	0.124086	3.848315	2.878585
H95	1.200171	4.936940	3.765623
H96	-0.904136	7.069915	4.492237
H97	-0.840190	8.086814	3.049970
H98	0.659778	7.519434	3.799115
H99	-0.149575	8.179471	0.888550
H100	0.246148	7.591799	-3.963538
H101	0.673645	-0.862912	9.047870

H102	4.763519	0.440949	8.815920
H103	0.527701	-0.846895	6.568002
H104	4.600539	0.473255	6.332234
H105	1.645126	-0.437267	4.571358
H106	2.922145	-6.035434	4.466991
H107	3.786473	-5.330969	5.833983
H108	0.732806	-5.699296	5.653429
H109	7.637316	1.663576	2.681962
H110	7.714970	-0.015274	3.154609
H111	6.578692	0.964552	0.491006
H112	5.547541	-1.300193	0.344743
H113	6.357268	-1.781944	1.840279
H114	7.305809	-1.427960	0.385357
H115	5.237844	1.821761	2.416244
H116	4.335805	0.628657	1.478374
H117	5.042387	0.176967	3.032913
H118	9.253637	-0.548997	1.269630
H119	8.262607	4.073049	-0.734657
H120	1.598882	4.918139	-2.986846
H121	1.816603	2.523694	-4.916322
H122	-5.715926	5.538715	-3.060434
H123	-6.401710	4.303370	-1.007033
H124	-7.313737	3.715113	-2.402500
H125	-5.948106	2.769914	-1.774277
H126	-3.914180	5.209597	-1.402253
H127	-3.235049	5.064944	-3.022703
H128	-3.549409	3.604302	-2.075603
H129	-4.948056	2.787870	-4.169155
H130	-1.767412	-1.013896	-3.167986
H131	-3.215887	-2.007825	-3.114378
H132	-2.933919	0.764214	-4.431011
H133	-1.584623	-0.913733	-8.069601
H134	2.318386	-2.886761	-6.912478
H135	3.821440	-2.140391	-6.406822
H136	2.842600	-3.605719	-4.699449
H137	3.208378	-2.137485	-2.758626
H138	2.990903	-0.692657	-3.739623
H139	4.380667	-1.755669	-4.025701
H140	0.855859	-2.966096	-3.385022
H141	0.598065	-1.538015	-4.400091
H142	0.408400	-3.156764	-5.088934
H143	2.191450	-0.059150	-5.794177
H144	-1.589432	-4.254230	-3.902171
H145	-0.488197	-5.483293	-4.548861
H146	-2.436634	-7.148855	-4.425934
H147	-6.485316	-5.320021	-3.846962
H148	-4.619416	-3.882733	0.630303
H149	-6.453883	-3.760509	2.359399
H150	-4.871150	-3.920828	3.102709
H151	-5.941600	-5.329156	2.992201
H152	-3.438117	-6.058968	0.236372
H153	-4.226318	-6.747113	1.664729
H154	-6.518467	-6.283925	0.599988
H155	-2.234741	-2.726184	6.546426
H156	-1.327013	-3.442227	5.222395
H157	-3.177511	-5.134617	4.871925
H158	8.574923	1.171120	-5.323174
H159	10.038368	-0.403054	-4.041898
H160	10.728061	-1.365223	-1.290139
H161	11.477829	-2.983840	-1.390208
H162	-7.416051	-0.226657	0.662628
H163	-7.421738	1.334411	1.529757
H164	-7.111735	1.292439	-0.205549
H165	-3.113233	-0.637005	2.760907
C166	-0.905751	-3.162671	1.015763
C167	0.077166	-2.145479	0.652742
O168	-0.803330	-2.714593	-0.389947
C169	1.462028	-2.748807	0.660443
C170	-0.189577	-4.469616	1.279118
C171	1.213186	-4.277445	0.648023
H172	-0.085660	-1.090468	0.842528
H173	2.085099	-2.404226	-0.170973
H174	1.982029	-4.819889	1.202492
H175	1.938649	-2.436477	1.594976
H176	1.221301	-4.650999	-0.378594
H177	-0.103950	-4.581500	2.365378
H178	-0.723774	-5.341219	0.889348
H179	-1.841319	-2.909731	1.505659
H180	2.105467	4.219584	-5.321254
H181	8.243487	1.281538	-3.585853
H182	8.963433	-1.308217	-5.098794
H183	9.918839	4.462666	-0.216849
H184	9.633184	3.134788	-1.365800
H185	9.895456	0.723914	2.298485
H186	2.795803	-0.233601	10.188559
H187	1.822944	-6.727980	6.605338
H188	1.583066	-5.031899	7.055150
H189	-2.641627	-5.173572	6.558427
H190	-4.143565	-4.330442	6.112527
H191	-6.999944	-4.630139	0.267034
H192	-6.877410	-7.051908	-3.940794
H193	-2.633757	-5.840838	-5.584994
H194	2.861083	-0.313244	-7.400502
H195	-1.286618	0.817776	-8.291241
H196	-4.502731	-0.027271	-4.181028
H197	-3.103514	-0.038087	1.099609
H198	-4.925348	0.748999	0.094576
H199	0.459748	9.285707	-3.457119
H200	1.692615	8.065243	-3.048074
H201	-4.599331	10.820280	0.168761
H202	-4.227952	9.512649	-0.980377
H203	-6.345925	7.037979	2.039872
H204	7.130206	0.405547	-4.647169
H205	11.587778	-1.871924	-2.770880
H206	8.433799	-0.785270	-2.122416
H207	7.432785	-1.784040	-3.189477
H208	-1.219665	6.989592	0.174562
H209	-2.279628	0.272210	-6.934687
H210	-6.315765	3.768561	-4.720704
H211	-4.657772	4.335130	-4.976710
H212	-3.284262	-0.779902	-5.224922
H213	3.211469	3.375234	-4.224789
H214	4.254536	-3.153606	5.525257
H215	3.281591	-2.027277	4.643322
H216	8.858879	2.981791	1.490161
H217	0.703505	-1.192868	-8.071457
H218	-0.985634	8.176262	-1.764581
H219	-6.166140	9.117324	1.233669
H220	-5.134182	-0.989030	3.739913
H221	-6.608690	-0.252308	3.070974

H222	-5.168754	-4.892638	-1.650628
H223	-4.593248	-7.542018	-4.044238
H224	-1.966873	-1.561600	-0.808628
H225	-0.528620	-1.274887	3.893538
H226	0.766938	-2.144319	3.799880
S227	-0.992070	-5.876426	-2.206656
H228	-0.885462	-4.651265	-1.652951
H229	-3.068096	-5.424757	1.850683
C230	1.400868	2.966366	-2.139740
C231	1.923178	3.398037	-0.913582
H232	2.237300	4.433065	-0.805348
C233	2.020276	2.520885	0.169939
H234	2.419913	2.875057	1.116067
C235	1.605036	1.195001	0.041823
H236	1.679682	0.520129	0.890258
C237	0.983664	1.631826	-2.260202
H238	0.574038	1.280989	-3.204634
C239	1.089506	0.754456	-1.180901
H240	0.254969	3.987648	-3.639756
H241	0.763583	-0.275283	-1.297628
S242	-5.578851	4.326726	2.986787
H243	-5.217186	3.139196	2.458931

TS attack at C1:

C1	-5.235652	6.795804	1.969309
C2	-4.674371	7.720152	0.886911
O3	-3.684623	7.438032	0.215047
C4	-4.862398	5.336258	1.728969
N5	-5.348991	8.898233	0.755527
C6	-4.929649	9.954960	-0.146217
C7	-0.113755	7.146992	0.537564
C8	0.703434	7.320837	-0.739499
O9	1.878986	6.971631	-0.840909
C10	0.454539	6.069283	1.464281
C11	-0.324855	5.875516	2.778992
C12	0.212623	4.652998	3.537485
C13	-0.295000	7.120774	3.678199
N14	0.039012	7.952045	-1.753236
C15	0.669801	8.262272	-3.022900
C16	2.759370	-0.453441	9.073028
C17	1.531282	-0.748895	8.473904
C18	3.846696	-0.135640	8.254040
C19	1.384316	-0.744857	7.083372
C20	3.714715	-0.124351	6.866110
C21	2.483083	-0.434897	6.273749
O22	2.442863	-0.454032	4.902707
C23	1.640655	-5.886294	6.076692
C24	2.821193	-5.491506	5.180912
C25	2.583914	-4.109849	4.601401
N26	3.547491	-3.183535	4.773048
O27	1.525968	-3.871057	3.997353
C28	9.099328	0.343664	1.534309
C29	9.290657	1.221944	0.317371
O30	9.544838	0.775754	-0.801537
C31	7.732851	0.497718	2.236228
C32	6.495860	0.243322	1.351620
C33	6.419275	-1.208874	0.857776
C34	5.213793	0.633517	2.101346
N35	9.177303	2.552498	0.572017
C36	9.259906	3.540228	-0.472071
C37	8.162960	0.603095	-4.496619
C38	8.914455	-0.713018	-4.265630
C39	8.348537	-1.479898	-3.068239
S40	9.227152	-3.036949	-2.639419
C41	10.811000	-2.359138	-2.016628
C42	2.159740	3.514157	-4.469252
C43	1.322868	4.004121	-3.277363
C44	-5.313248	3.907708	-4.187124
C45	-5.296409	4.488251	-2.769211
C46	-6.304040	3.741670	-1.883759
C47	-3.882450	4.437951	-2.172236
C48	-6.940197	0.771594	0.763230
N49	-5.504411	0.572626	0.857716
C50	-4.887205	-0.007440	1.905499
N51	-5.540114	-0.285853	3.049507
N52	-3.582678	-0.257268	1.830467
C53	-3.463381	-0.122630	-4.311040
C54	-2.874990	-0.885091	-3.117491
C55	-3.224183	-0.305476	-1.745079
O56	-4.170848	0.493484	-1.622614
O57	-2.525161	-0.719060	-0.733137
C58	-1.452821	0.151087	-7.545616
C59	-0.184303	0.162140	-6.717785
O60	-0.048223	0.884151	-5.732094
N61	0.788461	-0.674923	-7.176031
C62	2.151801	-0.673250	-6.677273
C63	2.643069	-2.039740	-6.157811
C64	2.351146	-2.366340	-4.675571
C65	3.192841	-1.503531	-3.722484
C66	0.860641	-2.276130	-4.320674
C67	-2.617481	-6.069813	-4.527633
C68	-3.942132	-5.605225	-3.928992
O69	-4.126566	-4.451630	-3.534651
C70	-1.424679	-5.226407	-4.089561
N71	-4.931190	-6.536286	-3.930172
C72	-6.310313	-6.227348	-3.569921
C73	-6.632287	-6.300674	-2.068721
O74	-7.516849	-7.042032	-1.651997
N75	-5.887834	-5.478578	-1.285206
C76	-6.206255	-5.313121	0.126216
C77	-5.037193	-4.813321	0.980592
C78	-3.878763	-5.818312	1.012759
C79	-5.559309	-4.502407	2.389930
C80	-3.145000	-4.668311	5.709294
C81	-2.312093	-3.395810	5.504020
C82	-2.971848	-2.356930	4.581256
O83	-4.213399	-2.213998	4.636035
O84	-2.200245	-1.673680	3.813957
O85	0.335018	-1.536611	3.361667
H86	-5.335085	4.965456	0.818606
H87	-3.781385	5.243587	1.621968
H88	-4.820739	7.129528	2.930408
H89	-5.746821	10.248060	-0.813228
H90	0.473637	5.118370	0.920977
H91	1.501546	6.309073	1.686683
H92	-1.375516	5.672753	2.520337
H93	-0.362959	4.464511	4.450166

H94	0.169887	3.750391	2.919218
H95	1.258863	4.806018	3.830667
H96	-0.836397	6.940963	4.613506
H97	-0.752126	7.991281	3.197057
H98	0.737042	7.387218	3.938641
H99	-0.112871	8.123653	1.040840
H100	0.319860	7.604296	-3.827826
H101	0.670711	-0.990287	9.091493
H102	4.808178	0.109074	8.697089
H103	0.427072	-0.982780	6.627225
H104	4.552957	0.131405	6.225059
H105	1.548583	-0.645804	4.535890
H106	2.901474	-6.190990	4.340761
H107	3.767483	-5.534024	5.730708
H108	0.708054	-5.837274	5.511628
H109	7.652366	1.504633	2.669664
H110	7.710416	-0.189090	3.093460
H111	6.574163	0.895903	0.470236
H112	5.530746	-1.357483	0.235489
H113	6.350916	-1.904649	1.703882
H114	7.287832	-1.493872	0.257289
H115	5.255614	1.670043	2.454562
H116	4.335857	0.545505	1.453924
H117	5.049478	-0.006374	2.977093
H118	9.248587	-0.686447	1.204824
H119	8.279860	3.973837	-0.706479
H120	1.649389	5.004888	-2.975075
H121	1.781133	2.560568	-4.848688
H122	-5.606629	5.542350	-2.829179
H123	-6.341897	4.158494	-0.871278
H124	-7.316880	3.791410	-2.300480
H125	-6.017326	2.686715	-1.804927
H126	-3.823089	4.984476	-1.225718
H127	-3.153042	4.890914	-2.853450
H128	-3.576429	3.400432	-1.995164
H129	-5.001289	2.856897	-4.175929
H130	-1.784192	-0.950703	-3.195053
H131	-3.242791	-1.921362	-3.115538
H132	-2.961652	0.839187	-4.449663
H133	-1.628112	-0.806510	-8.044963
H134	2.219685	-2.828010	-6.796130
H135	3.729423	-2.084285	-6.307423
H136	2.666411	-3.409889	-4.531492
H137	3.050871	-1.825146	-2.685144
H138	2.910282	-0.446328	-3.768219
H139	4.262066	-1.580151	-3.950900
H140	0.685525	-2.630486	-3.299320
H141	0.500232	-1.244935	-4.385182
H142	0.252855	-2.886580	-4.997207
H143	2.194819	0.088550	-5.896689
H144	-1.658843	-4.168216	-4.198513
H145	-0.560596	-5.455984	-4.716395
H146	-2.448355	-7.129726	-4.305462
H147	-6.528518	-5.217792	-3.933210
H148	-4.668749	-3.873368	0.539962
H149	-6.407543	-3.809593	2.353863
H150	-4.798939	-4.039577	3.021655
H151	-5.902475	-5.418438	2.886512
H152	-3.486646	-6.018688	0.010929
H153	-4.208338	-6.772946	1.440646
H154	-6.559109	-6.282621	0.491546
H155	-2.163536	-2.899255	6.473542
H156	-1.313745	-3.632419	5.120846
H157	-3.243677	-5.232167	4.776069
H158	8.569528	1.147422	-5.355577
H159	9.973471	-0.492911	-4.088567
H160	10.620009	-1.509197	-1.357120
H161	11.302006	-3.157588	-1.455487
H162	-7.503693	-0.168446	0.843600
H163	-7.303189	1.467586	1.529435
H164	-7.151900	1.211799	-0.211170
H165	-3.106291	-0.728593	2.618139
C166	-0.879859	-2.858254	0.970390
C167	0.144383	-1.814760	1.140689
O168	-0.831148	-2.438221	-0.413387
C169	1.506338	-2.382798	0.873749
C170	-0.159617	-4.186083	1.142347
C171	1.275049	-3.900966	0.634853
H172	-0.104923	-0.763684	1.138677
H173	1.979023	-1.874557	0.028146
H174	2.014751	-4.507909	1.160137
H175	2.133180	-2.186718	1.746979
H176	1.349564	-4.124103	-0.431236
H177	-0.146242	-4.440530	2.206499
H178	-0.657068	-4.995718	0.602839
H179	-1.852408	-2.731903	1.443639
H180	2.122619	4.235989	-5.293247
H181	8.242410	1.250684	-3.617171
H182	8.870387	-1.344922	-5.160893
H183	9.939820	4.351666	-0.191650
H184	9.644881	3.046736	-1.364337
H185	9.897149	0.566385	2.255092
H186	2.862336	-0.461227	10.153043
H187	1.766993	-6.904112	6.456051
H188	1.555115	-5.212961	6.935210
H189	-2.681708	-5.325816	6.452149
H190	-4.150609	-4.407286	6.046313
H191	-7.052565	-4.617762	0.232636
H192	-6.972586	-6.935924	-4.068303
H193	-2.720421	-5.993765	-5.620212
H194	2.815675	-0.340088	-7.486383
H195	-1.387483	0.929993	-8.313596
H196	-4.528145	0.065540	-4.154612
H197	-3.139989	-0.260423	0.902960
H198	-4.958091	0.639575	-0.019998
H199	0.474096	9.300999	-3.310022
H200	1.743249	8.118583	-2.897991
H201	-4.585916	10.840025	0.401568
H202	-4.104785	9.566139	-0.743118
H203	-6.323737	6.909050	2.041363
H204	7.097735	0.428028	-4.687993
H205	11.467557	-2.049244	-2.833103
H206	8.345926	-0.840580	-2.180983
H207	7.315862	-1.790117	-3.265190
H208	-1.162956	6.939715	0.293092
H209	-2.298974	0.387062	-6.901879
H210	-6.315198	3.954781	-4.629717
H211	-4.629189	4.450687	-4.849079
H212	-3.345607	-0.708470	-5.227353
H213	3.208881	3.383956	-4.183047

H214	4.325392	-3.373269	5.385089
H215	3.325400	-2.206317	4.582312
H216	8.913273	2.845028	1.499962
H217	0.599356	-1.201459	-8.016026
H218	-0.948675	8.125380	-1.636718
H219	-6.147623	9.060289	1.350004
H220	-5.085043	-0.946849	3.709399
H221	-6.541585	-0.192058	3.078239
H222	-5.270976	-4.816955	-1.747290
H223	-4.699943	-7.493941	-4.147270
H224	-1.593831	-1.679187	-0.634547
H225	-0.639735	-1.604296	3.644796
H226	0.751270	-2.398339	3.604667
S227	-0.919438	-5.526700	-2.336680
H228	-1.250906	-4.319175	-1.834350
H229	-3.055664	-5.445684	1.631878
C230	1.411767	3.076326	-2.084941
C231	2.025253	3.494522	-0.896994
H232	2.395022	4.513393	-0.820614
C233	2.127455	2.629757	0.194732
H234	2.598908	2.972990	1.111487
C235	1.616607	1.333903	0.113897
H236	1.697326	0.672906	0.972596
C237	0.895954	1.773716	-2.154778
H238	0.410319	1.440183	-3.069001
C239	0.995911	0.908753	-1.065067
H240	0.275505	4.093143	-3.594800
H241	0.568360	-0.087049	-1.137609
S242	-5.443521	4.315699	3.154916
H243	-5.059531	3.124851	2.653164

TS attack at C2:

C1	-5.057344	6.905727	1.981686
C2	-4.445416	7.837178	0.932720
O3	-3.433149	7.554553	0.296057
C4	-4.550173	5.473189	1.839554
N5	-5.109337	9.019844	0.786004
C6	-4.660702	10.076086	-0.101787
C7	0.077292	7.136090	0.571193
C8	0.912249	7.316118	-0.693966
O9	2.094504	6.984843	-0.773853
C10	0.655728	6.077114	1.513342
C11	-0.115104	5.895795	2.834742
C12	0.434293	4.686092	3.605260
C13	-0.088506	7.152373	3.718000
N14	0.254670	7.934625	-1.719927
C15	0.903076	8.264819	-2.975463
C16	2.718269	-0.619469	9.041857
C17	1.488812	-0.911246	8.444333
C18	3.792828	-0.256041	8.224825
C19	1.328426	-0.859659	7.056104
C20	3.647300	-0.196988	6.839445
C21	2.414704	-0.505012	6.249115
O22	2.360653	-0.475714	4.876833
C23	1.468564	-5.991798	5.988697
C24	2.757390	-5.410208	5.410617
C25	2.505606	-4.034259	4.820556
N26	3.551441	-3.192043	4.708284
O27	1.367103	-3.730052	4.435089
C28	9.104952	0.083760	1.533295
C29	9.313964	0.967264	0.322928
O30	9.536794	0.524029	-0.803763
C31	7.739905	0.259546	2.232426
C32	6.500900	0.007542	1.350350
C33	6.408566	-1.449885	0.874813
C34	5.223334	0.419182	2.096148
N35	9.252849	2.298157	0.593319
C36	9.356797	3.294276	-0.441172
C37	8.198152	0.426071	-4.498015
C38	8.869181	-0.933941	-4.273731
C39	8.257494	-1.671244	-3.080227
S40	9.049190	-3.273970	-2.650642
C41	10.666663	-2.682843	-2.025673
C42	2.273307	3.493427	-4.462990
C43	1.616217	4.098140	-3.213121
C44	-5.187781	4.080402	-4.202918
C45	-5.097465	4.617974	-2.769779
C46	-6.082499	3.872874	-1.858224
C47	-3.663351	4.514696	-2.230648
C48	-6.914846	0.940332	0.710878
N49	-5.504520	0.613974	0.793279
C50	-4.909444	0.090299	1.875771
N51	-5.574986	-0.101763	3.033564
N52	-3.609496	-0.198129	1.824971
C53	-3.443835	0.004249	-4.359816
C54	-2.927750	-0.822251	-3.175038
C55	-3.293722	-0.276499	-1.792626
O56	-4.191825	0.579433	-1.673886
O57	-2.666995	-0.777761	-0.775867
C58	-1.414769	0.256357	-7.584578
C59	-0.148561	0.226578	-6.755699
O60	0.003416	0.935382	-5.762980
N61	0.806035	-0.626068	-7.222950
C62	2.163731	-0.670755	-6.711852
C63	2.606917	-2.058654	-6.204577
C64	2.309732	-2.383990	-4.723093
C65	3.174029	-1.543993	-3.769759
C66	0.821169	-2.259958	-4.367460
C67	-2.727766	-5.885063	-4.546465
C68	-4.077262	-5.421142	-4.005212
O69	-4.270052	-4.264105	-3.624595
C70	-1.550612	-5.167676	-3.891641
N71	-5.071447	-6.345355	-4.038409
C72	-6.452677	-6.030465	-3.688275
C73	-6.784819	-6.112685	-2.189408
O74	-7.692159	-6.833829	-1.786452
N75	-6.022465	-5.318721	-1.394363
C76	-6.338421	-5.155111	0.016961
C77	-5.144232	-4.733316	0.880580
C78	-4.068270	-5.824975	0.937034
C79	-5.654513	-4.366490	2.280432
C80	-3.282091	-4.645022	5.616648
C81	-2.527603	-3.308271	5.590279
C82	-3.158957	-2.250783	4.667461
O83	-4.402076	-2.128248	4.658475
O84	-2.358403	-1.528882	3.965426
O85	0.118091	-1.666409	3.304738

H86	-4.830254	5.063146	0.868203
H87	-3.462997	5.455870	1.919162
H88	-4.795818	7.296026	2.974221
H89	-5.436578	10.336431	-0.829547
H90	0.681714	5.117805	0.984804
H91	1.701286	6.329405	1.728403
H92	-1.165914	5.682643	2.585141
H93	-0.131348	4.508396	4.526390
H94	0.389319	3.774619	3.000173
H95	1.482638	4.847564	3.885942
H96	-0.625074	6.981650	4.657804
H97	-0.552456	8.013864	3.227524
H98	0.943192	7.427499	3.970532
H99	0.050688	8.116307	1.066857
H100	0.557417	7.625134	-3.796588
H101	0.637887	-1.187450	9.060569
H102	4.754751	-0.012725	8.667556
H103	0.370373	-1.097156	6.601482
H104	4.475374	0.093671	6.200126
H105	1.463762	-0.654188	4.527450
H106	3.142902	-6.051995	4.607082
H107	3.548007	-5.355444	6.168067
H108	0.693372	-6.045484	5.223266
H109	7.670017	1.272182	2.653950
H110	7.709740	-0.417744	3.097090
H111	6.584940	0.648085	0.460759
H112	5.516608	-1.597491	0.257340
H113	6.335458	-2.134313	1.729964
H114	7.272033	-1.751710	0.275268
H115	5.270087	1.461900	2.429435
H116	4.342688	0.319924	1.454265
H117	5.060985	-0.203690	2.984839
H118	9.235136	-0.946847	1.197423
H119	8.386170	3.749541	-0.673914
H120	2.127078	5.019827	-2.916044
H121	1.724655	2.613626	-4.808859
H122	-5.381192	5.681151	-2.787613
H123	-6.059679	4.264808	-0.834932
H124	-7.112272	3.960265	-2.224540
H125	-5.820567	2.809433	-1.818545
H126	-3.562961	5.016562	-1.262414
H127	-2.948157	4.983234	-2.916417
H128	-3.373949	3.464753	-2.106958
H129	-4.905087	3.021760	-4.235185
H130	-1.838653	-0.935706	-3.219592
H131	-3.340801	-1.839757	-3.221268
H132	-2.887235	0.940136	-4.461580
H133	-1.598843	-0.680759	-8.118736
H134	2.149991	-2.826282	-6.844840
H135	3.689879	-2.144333	-6.360130
H136	2.601148	-3.435291	-4.584254
H137	3.041726	-1.874141	-2.733917
H138	2.906348	-0.482457	-3.800461
H139	4.239321	-1.633249	-4.011503
H140	0.635367	-2.605306	-3.344378
H141	0.482849	-1.221510	-4.435319
H142	0.198969	-2.858871	-5.041447
H143	2.219544	0.080595	-5.922093
H144	-1.745744	-4.095984	-3.871026
H145	-0.637385	-5.346265	-4.462535
H146	-2.624834	-6.972799	-4.459827
H147	-6.660461	-5.017111	-4.046709
H148	-4.699363	-3.830454	0.433569
H149	-6.424983	-3.589276	2.232056
H150	-4.859906	-3.983965	2.923740
H151	-6.096601	-5.240706	2.774026
H152	-3.689815	-6.074140	-0.058728
H153	-4.471333	-6.741501	1.384953
H154	-6.746899	-6.107688	0.367786
H155	-2.524627	-2.875901	6.600488
H156	-1.479217	-3.455773	5.310434
H157	-3.238652	-5.144900	4.643244
H158	8.638224	0.950353	-5.352909
H159	9.939323	-0.778082	-4.095217
H160	10.521346	-1.819200	-1.372294
H161	11.110106	-3.503788	-1.457470
H162	-7.557015	0.072868	0.917717
H163	-7.186292	1.753083	1.396247
H164	-7.121074	1.274166	-0.306058
H165	-3.157945	-0.615830	2.653809
C166	-0.740110	-2.700728	1.503994
C167	-0.041934	-1.937200	0.466744
O168	-0.980579	-2.525278	-0.459496
C169	1.332445	-2.574097	0.330574
C170	-0.158150	-4.071680	1.614848
C171	1.098551	-4.061059	0.699905
H172	-0.104497	-0.852518	0.498269
H173	1.744159	-2.441903	-0.672945
H174	1.964898	-4.497743	1.202371
H175	2.007540	-2.089996	1.043658
H176	0.908494	-4.649103	-0.200381
H177	0.085411	-4.269066	2.662856
H178	-0.900907	-4.822360	1.324468
H179	-1.702494	-2.407883	1.904953
H180	2.290715	4.218383	-5.284912
H181	8.314896	1.061516	-3.613988
H182	8.788333	-1.557829	-5.172037
H183	10.051779	4.088824	-0.150208
H184	9.734051	2.802587	-1.337748
H185	9.904509	0.287005	2.257897
H186	2.832235	-0.664153	10.119934
H187	1.643549	-6.997760	6.380802
H188	1.088639	-5.369374	6.803318
H189	-2.853849	-5.323418	6.361531
H190	-4.334464	-4.476412	5.856314
H191	-7.143484	-4.413166	0.129721
H192	-7.115392	-6.731107	-4.197172
H193	-2.723192	-5.655470	-5.621714
H194	2.846121	-0.347362	-7.509271
H195	-1.340327	1.061421	-8.324325
H196	-4.499871	0.247600	-4.220904
H197	-3.171881	-0.269471	0.897325
H198	-4.942374	0.679052	-0.077022
H199	0.719945	9.310571	-3.245701
H200	1.973680	8.110102	-2.839808
H201	-4.382476	10.977781	0.455479
H202	-3.785254	9.704160	-0.633814
H203	-6.151642	6.936574	1.911975
H204	7.124658	0.316533	-4.692056
H205	11.342563	-2.415659	-2.841363

H206	8.285724	-1.034635	-2.191537
H207	7.210318	-1.924520	-3.281952
H208	-0.963631	6.907571	0.312447
H209	-2.261833	0.476934	-6.936712
H210	-6.205400	4.167529	-4.601070
H211	-4.516599	4.625130	-4.876567
H212	-3.338211	-0.562761	-5.289440
H213	3.307174	3.197108	-4.254665
H214	4.425052	-3.402064	5.164988
H215	3.353584	-2.221299	4.472018
H216	9.010136	2.589587	1.527451
H217	0.604635	-1.145708	-8.064234
H218	-0.734114	8.107992	-1.612809
H219	-5.933262	9.177863	1.346221
H220	-5.171568	-0.783099	3.705052
H221	-6.573934	0.023346	3.040698
H222	-5.393607	-4.662577	-1.848158
H223	-4.836495	-7.308162	-4.226794
H224	-1.746053	-1.793941	-0.682830
H225	-0.803945	-1.603808	3.719785
H226	0.582591	-2.415367	3.744117
S227	-1.260945	-5.731108	-2.153051
H228	-1.299664	-4.516211	-1.567317
H229	-3.220805	-5.504163	1.553602
C230	1.614584	3.130502	-2.050346
C231	2.212715	3.460179	-0.827812
H232	2.643694	4.449193	-0.697710
C233	2.231312	2.542593	0.226075
H234	2.692419	2.816545	1.171076
C235	1.658865	1.279593	0.069786
H236	1.672010	0.573882	0.896158
C237	1.032277	1.863342	-2.194152
H238	0.557024	1.593176	-3.133544
C239	1.059663	0.945225	-1.148116
H240	0.582140	4.378840	-3.456861
H241	0.600875	-0.026092	-1.297660
S242	-5.290378	4.435035	3.176111
H243	-4.598080	3.310475	2.906520
